# BZW2 promotes malignant progression in lung adenocarcinoma through enhancing the ubiquitination and degradation of GSK3β

**DOI:** 10.1038/s41420-024-01879-7

**Published:** 2024-02-29

**Authors:** Kai Jin, Yongmeng Li, Ruyuan Wei, Yanfei Liu, Shuai Wang, Hui Tian

**Affiliations:** 1 Department of Thoracic Surgery, Qilu Hospital of Shandong University, Jinan, Shandong China; 2 Department of Thoracic Surgery, the First Affiliated Hospital of Shandong First Medical University & Shandong Provincial Qianfoshan Hospital, Jinan, Shandong China; 3 Department of Cardiovascular Surgery, Qilu Hospital of Shandong University, Jinan, Shandong China; 4 Department of Anesthesiology, Qilu Children’s Hospital of Shandong University, Jinan, China

**Keywords:** Non-small-cell lung cancer, Oncogenes

## Abstract

The role of Basic leucine zipper and W2 domains 2 (BZW2) in the advancement of different types of tumors is noteworthy, but its involvement and molecular mechanisms in lung adenocarcinoma (LUAD) remain uncertain. Through this investigation, it was found that the upregulation of BZW2 was observed in LUAD tissues, which was associated with an unfavorable prognosis for individuals diagnosed with LUAD, as indicated by data from Gene Expression Omnibus and The Cancer Genome Atlas databases. Based on the clinicopathologic characteristics of LUAD patients from the tissue microarray, both univariate and multivariate analyses indicated that BZW2 functioned as an independent prognostic factor for LUAD. In terms of mechanism, BZW2 interacted with glycogen synthase kinase-3 beta (GSK3β) and enhanced the ubiquitination-mediated degradation of GSK3β through slowing down of the dissociation of the ubiquitin ligase complex, which consists of GSK3β and TNF receptor-associated factor 6. Moreover, BZW2 stimulated Wnt/β-catenin signaling pathway through GSK3β, thereby facilitating the advancement of LUAD. In conclusion, BZW2 was a significant promoter of LUAD. The research we conducted identified a promising diagnostic and therapeutic target for LUAD.

## Introduction

Non-small cell lung cancer (NSCLC) accounts for 80–85% of lung cancers which is the main cause of cancer deaths and the second most common cancer globally [1–4]. Lung adenocarcinoma (LUAD), which has substantial morbidity and mortality, is the prevailing pathological form of NSCLC [5]. Although there are several anti-cancer therapies that have been used to treat LUAD, including surgery, chemotherapy and irradiation, LUAD has a restricted range of treatments and continues to be a deadly disease due to the lack of an early-stage diagnostic platform, delayed symptom manifestation, genetic diversity, metastatic nature, and poor response to late-stage chemotherapy [6, 7]. Over the past few years, the molecular targeting diagnosis and treatment of LUAD, which significantly improve the prognosis of lung cancer patients, have become increasingly important, but only a specific subset of patients will benefit [8–10]. In view of this, it is vital to discover more biomarkers and therapeutic molecular targets for LUAD.

Basic leucine zipper and W2 domain 2 (BZW2), also referred to as eIF5-mimic protein 1, belongs to the basic-region leucine zipper superfamily of transcription factors [11]. BZW2 functions as a regulator of translation initiation and serves as a competitive inhibitor of the function of eukaryotic translation initiation factor 5 (EIF5), resulting in a reduction in translation that is initiated by non-AUG codons and repeat-associated non-AUG (RAN) codons [12–15]. Accumulating evidence suggests that BZW2 is an oncogene and promotes the progression of various kinds of cancers. In fibrosarcoma, BZW2 promotes tumorigenesis by stimulating the expression of cyclic AMP-dependent transcription factor ATF-4 [16]. In colorectal cancer, BZW2 promotes cell growth and metastasis by specifically activating the extracellular-signal-regulated kinase/mitogen-activated protein kinase signaling pathway [17]. In hepatocellular carcinoma and osteosarcoma, BZW2 enhances the malignant progression of tumors by the phosphoinositide 3-kinase/protein kinase B signaling pathway [18, 19]. Nevertheless, the involvement and molecular mechanisms of BZW2 in LUAD remain unreported.

We found that BZW2 promoted the ubiquitination and degradation of glycogen synthase kinase-3 beta (GSK3β), and ultimately activated the Wnt/β-catenin signaling pathway in LUAD. GSK3β is a serine/threonine protein kinase whose ubiquitination can be regulated by TNF receptor-associated factor 6 (TRAF6), which is a direct E3 ligase for GSK3β [20]. GSK3β is closely related to LUAD which has an impact on the survival, autophagy, metastasis, epithelial-mesenchymal transition (EMT), apoptosis and proliferation of LUAD cells [21–23]. The initial connection of GSK3β with cancer was established due to its participation in the Wnt/β-catenin signaling pathway, characterized by the nuclear accumulation of β-catenin. GSK3β is an element of the β-catenin destruction complex [24, 25]. The accumulation of β-catenin resulted from the degradation of GSK3β leads to aberrant activation of the Wnt/β-catenin signaling pathway. Dysfunctions in the activation of the Wnt/β-catenin signaling pathway and the consequent creation of nuclear lymphoid enhancer-binding factor (LEF)/transcription factor (TCF)/β-catenin complexes cause unregulated activation of target genes downstream, ultimately leading to the development and metastasis of tumors and cells malignant transformation [24, 26].

Through this investigation, BZW2 expression was higher in LUAD tissues compared to the normal lung tissues and was a valuable prognostic predictor in LUAD. The in vitro and in vivo experiments confirmed that BZW2 enhanced cell growth, migration and invasion of LUAD. Furthermore, we revealed for the first time that BZW2 decelerated the dissociation of the ubiquitin ligase complex consisting of GSK3β and TRAF6 and enhanced the ubiquitination-mediated degradation of GSK3β. In conclusion, our study indicates that BZW2 has the potential to be a target for the molecular diagnosis and targeted therapy of LUAD.

## Results

### The upregulation of BZW2 was related to a poorer prognosis of LUAD patients

**Fig. 1:**
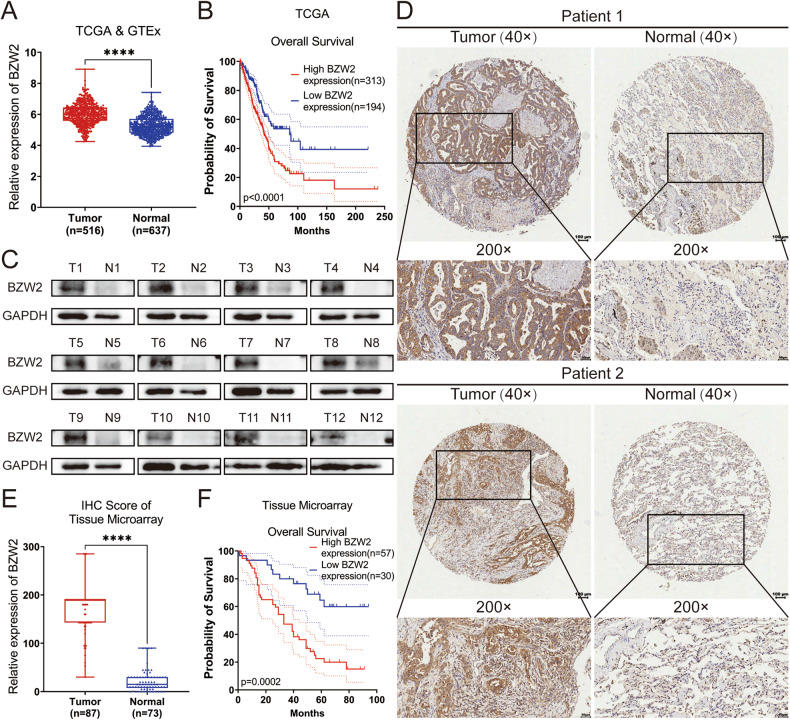
BZW2 is upregulated in LUAD samples and is correlated with a poorer prognosis in LUAD patients. **A** The expression of BZW2 was higher in tumor tissues than in normal lung tissues according to TCGA database. **B** Kaplan–Meier analysis for LUAD samples based on the expression of BZW2 according to TCGA database. **C** Western blot analyses showing the expression of BZW2 in 12 pairs of LUAD patients. **D** Representative tissue microarray immunohistochemical images of BZW2 expression in LUAD tissues and normal tissues. **E** IHC score of the BZW2 expression in the tissue microarray. **F** Kaplan–Meier analysis for LUAD samples based on the expression of BZW2 according to the tissue microarray. (****P < 0.0001).

According to the TCGA database, BZW2 is highly expressed in many different types of cancers, including LUAD, liver hepatocellular carcinoma and so on (Supplementary Fig. S1A). We compared the mRNA level of BZW2 in LUAD tissues and normal lung tissues in several datasets. According to the TCGA, GTEx and GEO databases, the mRNA level of BZW2 in LUAD tissues was elevated to compared to that in normal lung tissues (Fig. 1A, Supplementary Fig. S1B). The Kaplan-Meier analysis, using information from the TCGA and GEO databases, demonstrated a connection between increased expression of BZW2 and poorer overall survival in individuals with LUAD tissues (Fig. 1B, Supplementary Fig. S1C). For the purpose of the Western blot (WB) assay, we gathered a total of 12 sets of clinical samples to measure the protein expression of BZW2. The findings indicated that the levels of BZW2 protein expression in LUAD tissues were elevated compared to those in normal lung tissue (Fig. 1C). Subsequently, we performed IHC on a tissue microarray comprising 87 LUAD tissues and 73 neighboring lung tissues to evaluate the extent of BZW2 protein expression (Fig. 1D). According to the IHC score, BZW2 expression was elevated in LUAD tissues compared to normal lung tissues (Fig. 1E). Furthermore, the Kaplan-Meier analysis conducted on a tissue microarray demonstrated that LUAD patients with elevated BZW2 expression had a significantly poorer prognosis (Fig. 1F). The results of the correlation between BZW2 expression and clinicopathological characteristics showed that BZW2 expression was significantly related to N stage and TNM stage (Table 1). Crucially, the univariate and multivariate analyses based on the tissue microarray suggested that BZW2 expression was an independent prognostic factor for LUAD patients (Table 2). In conclusion, these findings suggested that BZW2 was elevated in LUAD tissues and served as a valuable prognostic predictor in LUAD.

**Table 1:** Correlation between BZW2 expression and clinicopathological characteristics.

	Variables	BZW2 expression		Total	*p* value
		low	high		
Age					0.372
	≤60	17	26	43	
	>60	13	31	44	
Sex					0.648
	male	19	32	51	
	Female	11	25	36	
T stage					0.080
	T1/T2	21	34	55	
	T3/T4	5	23	28	
N stage					**0.040**
	N0	19	23	42	
	N1-N3	10	34	44	
TNM stage					**<0.001**
	I/II	27	31	58	
	III/IV	2	26	28	

Bold values imply a *p*-value less than 0.05, indicating a statistical difference.

**Table 2:** Univariate and multivariate analyses of the factors correlated with overall survival of cancer patients.

variables	Univariate analysis				Multivariate analysis			
	HR	95%CI		*p* value	HR	95%CI		*p* value
		Low	High			Low	High	
BZW2 Expression (Low vs. High)	0.301	0.154	0.587	**<0.001**	0.348	0.165	0.735	**0.006**
Age (≤60 vs. >60)	0.517	0.300	0.892	**0.018**	0.744	0.422	1.312	0.307
Sex (Female vs. Male)	1.019	0.596	1.744	0.944				
T Stage (1-2 vs. 3-4)	0.636	0.365	1.108	0.110				
N Stage (0 vs. 1-3)	0.313	0.176	0.557	**<0.001**	0.416	0.210	0.825	**0.012**
TNM Stage (1-2 vs. 3-4)	0.363	0.210	0.628	**<0.001**	0.758	0.390	1.473	0.413

*HR* hazard ratio, *CI* confidence interval.

Bold values imply a *p*-value less than 0.05, indicating a statistical difference.

### BZW2 enhanced the proliferation, migration and invasion of LUAD cells in vitro

**Fig. 2:**
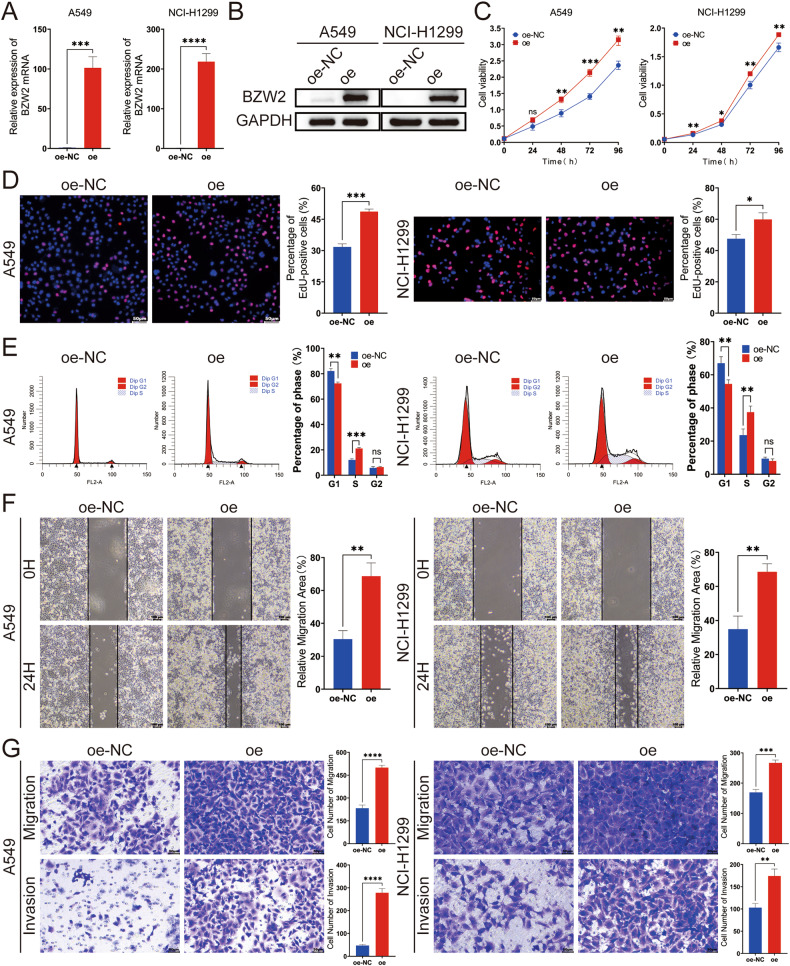
BZW2 promotes the proliferation, migration and invasion of LUAD in vitro. The overexpression efficiencies of BZW2 were confirmed by qRT-PCR (**A**) and western blot (**B**) in LUAD cells. CCK8 (**C**), EdU incorporation assays (**D**) and flow cytometry analyses (**E**) detected the proliferation ability in BZW2-overexpressing A549 cells, BZW2-overexpressing NCI-H1299 cells, and control cells. Wound healing assays (**F**) and transwell assays (**G**) detected the migration and invasion abilities in BZW2-overexpressing A549 cells, BZW2-overexpressing NCI-H1299 cells, and control cells. (ns, no significance, *P < 0.05, **P < 0.01, ***P < 0.001, ****P < 0.0001).

To examine the involvement of BZW2 in LUAD, we transfected LUAD cells with plasmid or siRNA to perform a range of experiments in vitro. Initially, we assessed the BZW2 expression level in LUAD cells by qRT-PCR and WB assays (Supplementary Fig. S2A, B). The results indicated that BZW2 expression was comparatively lower in A549 cells and NCI-H1299 cells, whereas the expression of BZW2 was comparatively higher in PC-9 cells. Consequently, we introduced plasmid into A549 and NCI-H1299 cells to enhance the expression of BZW2. In the meantime, we introduced siRNAs, specifically si-1 and si-2, into PC-9 cells to decrease the level of BZW2 expression. The qRT-PCR and WB assays demonstrated that BZW2 expression in LUAD cells transfected with BZW2 plasmid or siRNA exhibited a greater than twofold alteration compared to the control cells (Fig. 2A, B, Supplementary Fig. S3A, B). The results of CCK-8 and EdU assays indicated that the overexpression of BZW2 promoted LUAD cells proliferation, while the knockdown of BZW2 inhibited LUAD cells proliferation (Fig. 2C, D, Supplementary Fig. S3C, D). Flow cytometry results indicated that the overexpression of BZW2 caused an increasing proportion of cells in the S phase and a decreasing proportion of cells in the G1 phase, while the knockdown of BZW2 caused the opposite result of the overexpression (Fig. 2E, Supplementary Fig. S3E). The wound healing and Transwell assay results showed that the overexpression of BZW2 enhanced the migration and invasion abilities of LUAD cells, and the knockdown of BZW2 caused the opposite result of the overexpression (Fig. 2F, G, Supplementary Fig. S3F, G). Altogether, these findings suggested that BZW2 served as a tumor stimulator to promote the proliferation, migration and invasion of LUAD cells in vitro.

### BZW2 promoted tumor growth and metastasis in vivo

**Fig. 3:**
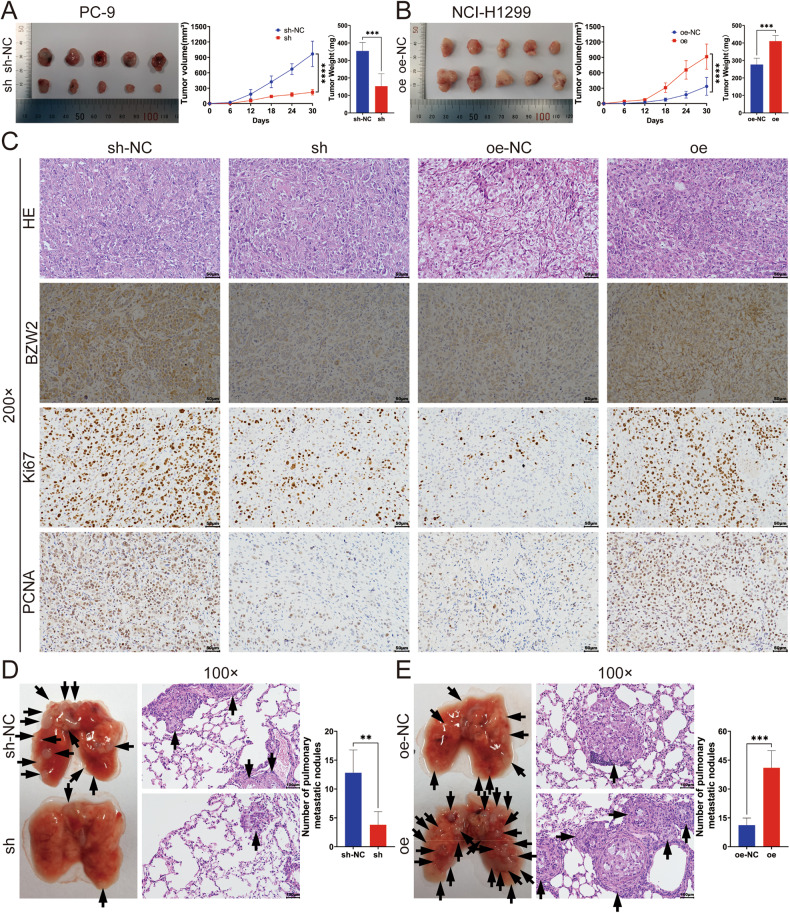
The overexpression of BZW2 promotes the proliferation, migration and invasion of LUAD in vivo. **A**, **B** Tumor pictures, tumor growth curves and tumor weights of different groups. **C** Representative pictures of H&E staining and immunohistochemical staining of BZW2, Ki67 and PCNA in each group. **D**, **E** Pictures of lung metastatic nodules(left) and H&E-stained images (right) of the lungs in each group; the black arrowheads denote lung metastasis nodules. (**P < 0.01, ***P < 0.001, ****P < 0.0001).

To investigate the involvement of BZW2 in vivo, we created a mouse model with subcutaneous xenografts and another mouse model with pulmonary metastasis. The sh-BZW2 lentivirus was transfected into PC-9 cells, while the oe-BZW2 lentivirus was transfected into NCI-H1299 cells. Then, the transduction efficiencies were assessed using qRT-PCR and WB assays (Supplementary Fig. S4A, B). Nude mice were injected with these cells and their corresponding control cells. In the sh cohort, the xenograft tumors had a significantly smaller average size, growth rate, and weight compared to the sh-NC cohort. Conversely, the oe cohort exhibited a larger average tumor size, growth rate, and weight compared to the oe-NC cohort (Fig. 3A, B). We used HE staining to evaluate tumor morphology and IHC analysis to evaluate BZW2 expression, Ki67 expression and PCNA expression (Fig. 3C). Confirmation of BZW2 knockdown through IHC staining revealed a decrease in the expression of BZW2, Ki67 and PCNA. Conversely, the oe cohort exhibited higher levels of BZW2, Ki67 and PCNA expression compared to the oe-NC cohort. In addition, we injected stable BZW2-knockdown PC-9 cells, control PC-9 cells, BZW2-overexpressing NCI-H1299 cells or control NCI-H1299 cells into BALB/c nude mice via the tail vein to establish a pulmonary metastasis mouse model in order to examine the involvement of BZW2 on LUAD metastasis. In the sh cohort, there were significantly fewer pulmonary metastatic nodules compared to the sh-NC cohort, whereas the oe cohort had significantly more pulmonary metastatic nodules than the oe-NC cohort (Fig. 3D, E). In vivo, BZW2 promoted tumor growth and metastasis in LUAD.

### BZW2 interacted with GSK3β

**Fig. 4:**
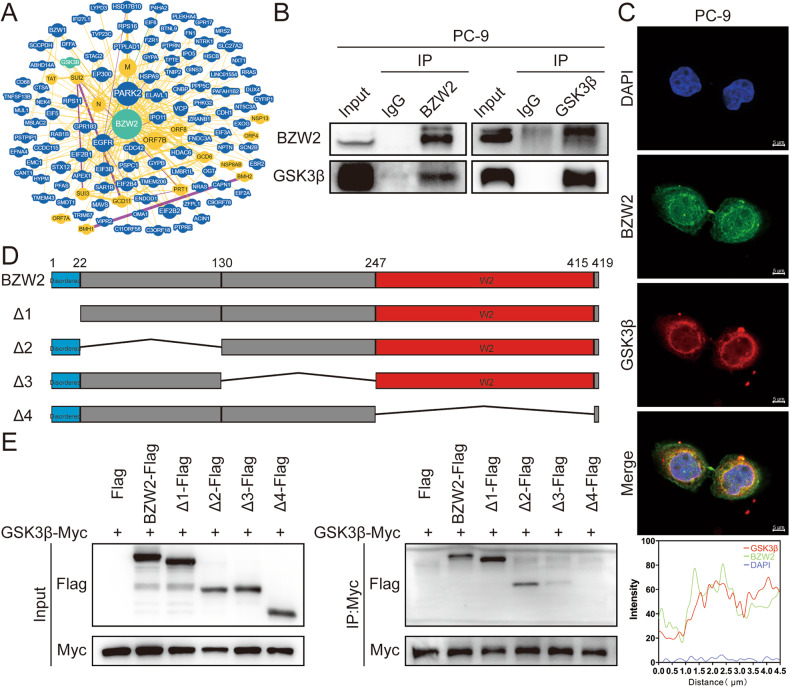
BZW2 interacts with GSK3β. **A** BZW2–protein interactions obtained by BioGRID. **B** Co-immunoprecipitation analyses of the interaction between BZW2 and GSK3β in PC-9 cells. **C** Immunofluorescent staining of the colocalization of BZW2 and GSK3β.The colocalization of BZW2 and GSK3β was shown by calculating the fluorescence intensities along the red arrow crossing the cytoplasm. **D** Truncated forms of BZW2 were constructed according to its functional domains. **E** Co-immunoprecipitation assay of truncated forms of BZW2-Flag and GSK3β-Myc in the whole cell lysates of 293FT cells.

We focused on the proteins that interact with BZW2 to investigate the mechanisms of BZW2 in LUAD. We found that BZW2 might interact with GSK3β based on the BioGRID and IntAct databases (Fig. 4A, Supplementary Fig. S5). To confirm this, we carried out co-IP analysis, and the results showed that BZW2 interacted with GSK3β (Fig. 4B). Subsequently, we also employed IF to identify the colocalization of BZW2 and GSK3β and found that BZW2 and GSK3β were predominantly colocalized in the cytoplasm (Fig. 4C). To determine the interacting domains, we used truncation and/or deletion analysis for co-IP analysis (Fig. 4D, E). The results showed that BZW2 interacted with GSK3β through amino acids 248-415, which was the W2 domain of BZW2. The above results demonstrated that BZW2 interacted with GSK3β.

### BZW2 promoted the ubiquitination-mediated degradation of GSK3β and activated the Wnt/β-catenin signaling pathway

**Fig. 5:**
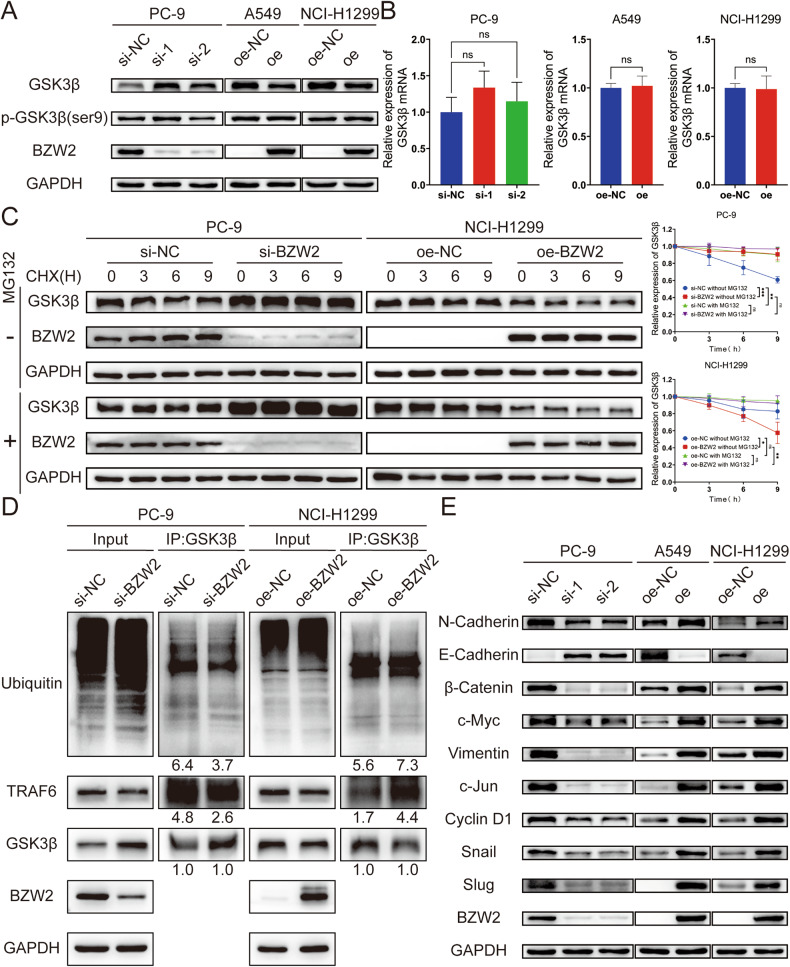
BZW2 activates Wnt/β-catenin signaling pathway by promoting ubiquitination-mediated degradation of GSK3β. Western blotting (**A**) and qRT-PCR (**B**) of GSK3β expression in BZW2-depleted PC-9 cells, BZW2-overexpressing A549 cells, BZW2-overexpressing NCI-H1299 cells, and control cells. **C** Western blotting of the effect of BZW2 on GSK3β stability in PC-9 and NCI-H1299 cells incubated with CHX or MG132 at the indicated time points. **D** Coimmunoprecipitation analyses were conducted to identify the function of BZW2 on the interplay between GSK3β, TRAF6 and ubiquitin in PC-9 and NCI-H1299 cells incubated with MG132. **E** Western blotting confirmed the effects of BZW2 OE and KD on the Wnt/β-catenin signaling pathway. (ns, no significance, *P < 0.05, **P < 0.01, ***P < 0.001).

BZW2 knockdown upregulated the expression of GSK3β, and BZW2 overexpression downregulated its expression (Fig. 5A). But there was no significant change on phosphorylated GSK3β, nor on the mRNA level of GSK3β (Fig. 5A, B). In addition to being phosphorylated, there was also ubiquitination and degradation of GSK3β [27, 28]. To investigate whether BZW2 affected the degradation of GSK3β, we employed a Cycloheximide (CHX) chasing assay (Fig. 5C). We found that BZW2 knockdown decelerated the degradation of GSK3β and that BZW2 overexpression accelerated this process. In addition, MG132 attenuated the degradation of GSK3β caused by BZW2. To go a step, we employed co-IP analysis to investigate whether BZW2 regulated the ubiquitination of GSK3β in LUAD cells (Fig. 5D). The results of co-IP showed that knockdown of BZW2 inhibited the ubiquitination-mediated degradation of GSK3β by accelerating the dissociation of the ubiquitin ligase complex consisting of GSK3β and TRAF6. Nevertheless, overexpression of BZW2 led to the opposite trend. GSK3β is a member of the β-catenin destruction complex, which is an important part of the Wnt/β-catenin signaling pathway. It is worth mentioning that KEGG pathway enrichment analysis and IPA of the transcriptome sequencing data also indicated that the knockdown of BZW2 had an impact on the Wnt/β-catenin signaling pathway (Supplementary Fig. S6A, B). Consequently, we conducted additional research on the impact of BZW2 on the Wnt/β-catenin signaling pathway. The WB assay results revealed that BZW2 knockdown led to an increase in E-cadherin expression and a decrease in the expression of N-cadherin, vimentin, Snail, as well as the Wnt/β-catenin signaling pathway targets, including β-catenin, c-Jun, c-Myc, Cyclin D1 and Slug (Fig. 5E). The overexpression of BZW2 led to the opposite result. To sum up, BZW2 promoted the ubiquitination-mediated degradation of GSK3β and subsequently activated the Wnt/β-catenin signaling pathway.

### Silencing GSK3β partly rescued the suppressive effect caused by BZW2 knockdown in LUAD cells

**Fig. 6:**
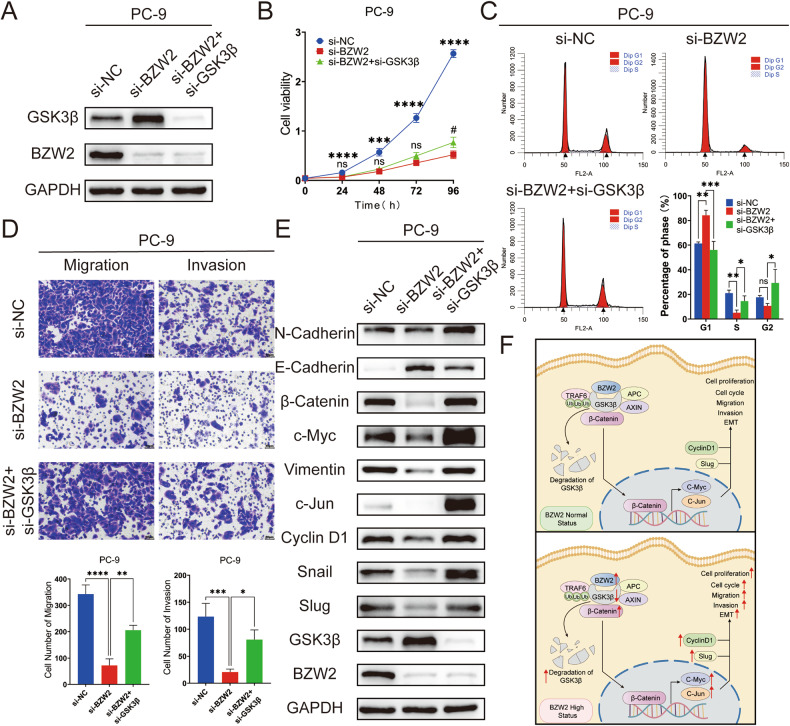
BZW2 promotes the proliferation, migration, invasion and Wnt/β-catenin signaling pathway of LUAD partly through regulating GSK3β. **A** Western blotting confirmed the transfection efficiency of BZW2 and GSK3β. CCK8 (**B**), flow cytometry analyses (**C**) and transwell assays (**D**) in different transfected group. **E** Western blotting confirmed the effects of BZW2 KD with GSK3β KD on the Wnt/β-catenin signaling pathway. **F** The working model of the role of BZW2 in LUAD. (ns, no significance, *P < 0.05, **P < 0.01, ***P < 0.001, ****P < 0.0001).

We conducted additional research to determine whether the influence of BZW2 on LUAD advancement occurs via GSK3β. The upregulated expression of GSK3β caused by the knockdown of BZW2 was rescued by the knockdown of GSK3β (Fig. 6A). Moreover, the suppressive effects of BZW2 knockdown on PC-9 cell growth, cell cycle, migration and invasion were partly restored by GSK3β knockdown (Fig. 6B–D). As mentioned previously, the targets of the Wnt/β-catenin signaling pathway and EMT were altered when BZW2 was knocked down. The WB assays showed that the inhibition of GSK3β in PC-9 cells that was depleted of BZW2 reversed the alterations in target protein expression related to the Wnt/β-catenin signaling pathway and EMT caused by BZW2 knockdown (Fig. 6E). In conclusion, BZW2 promoted the malignant progression of LUAD through the degradation and ubiquitination of GSK3β, thus activating the Wnt/β-catenin signaling pathway (Fig. 6F).

## Discussion

In this study, we confirmed that BZW2 expression was higher in LUAD patients and associated with a poorer prognosis. Meanwhile, BZW2 activated Wnt/β-catenin signaling pathway by enhancing the ubiquitination and degradation of GSK3β, which promoted the growth and metastasis of LUAD. Specifically, this study indicated that BZW2 could be a diagnostic molecule and therapeutic target for LUAD.

BZW2 has been reported to be implicated in different types of cancers, such as colorectal cancer, muscle-invasive bladder cancer, hepatocellular cancer and osteosarcoma [18, 19, 29, 30]. The present investigation offers crucial proof of the cancer-causing role of BZW2 in vitro and in vivo, while also explaining the underlying mechanism in LUAD. In the development of LUAD, BZW2 was confirmed to activate Wnt/β-catenin signaling pathway which was related with EMT, cell cycle and c-Myc as favorable downstream signaling pathways according to mechanistic analysis. The abnormal activation of Wnt/β-catenin signaling pathway, which impacts on a number of signaling pathways, such as embryogenesis, cell growth, invasion and organogenesis, has been confirmed to have association with approximately half of all human malignancies, including LUAD [31, 32]. The abnormal activation of Wnt/β-catenin signaling pathway results in an increase of prevalence, progression of malignancy, and the development of poor prognostics [33–37]. Metastasis is the primary reason for death in LUAD patients, and Wnt/β-catenin signaling pathway has strongly association with EMT [38]. Furthermore, the creation of β-catenin/LEF/TCF complex to the *CD274* promoter region caused by the accumulation of β-catenin stimulates the PD-L1 expression, further promoting tumor immune evasion and weakening the impact of PD-1/PD-L1 inhibitors [39, 40]. To sum up, the activation of Wnt/β-catenin signaling pathway via BZW2 is crucial for the molecular therapy of LUAD.

The aberrant buildup of β-catenin protein in the nucleus is the crucial event for the activation of Wnt/β-catenin signaling pathway [41]. After synthesized in the cytoplasm, large amounts of β-catenin are transported to the nucleus to interact with the TCF/LEF, resulting in transcriptional activation of various downstream target genes, including c-Jun, Cyclin D1 c-Myc and so on. In the cytoplasm, the phosphorylation-dependent degradation of β-catenin is usually caused by the activation of destruction complex, which consists of GSK3β, CK1α, AXIN and APC [25, 42].GSK3β plays a crucial role as a key element in the destruction complex. In this study, we found a new GSK3β-associated protein, BZW2, whose W2 domain interacted with GSK3β in the cytoplasm. BZW2 enhanced the ubiquitination and degradation of GSK3β and subsequently activated Wnt/β-catenin signaling pathway. The identification of the finding offered a fresh mechanism through which BZW2 regulated the protein expression linked to proliferation, cell cycle and metastasis. Furthermore, we found that GSK3β could also be ubiquitinated rather than phosphorylated in LUAD. It has been reported that TRAF6 interacted with GSK3β and affected the ubiquitination and activity of GSK3β [20]. According to our study, BZW2 overexpression resulted in the stabilization of the interaction between the E3 ubiquitin ligase TRAF6 and GSK3β, consequently leading to the ubiquitination of GSK3β. In addition, we confirmed that BZW2 activated Wnt/β-catenin signaling pathway mediated by GSK3β, potentiating the growth and metastasis of LUAD cells. The positive correlation between BZW2 and the Wnt/β-catenin signaling pathway was confirmed by the results of KEGG pathway enrichment analysis and the IPA of the transcriptome sequencing data.

In addition, here is an interesting conjecture. c-Myc, as well known as a transcription factor, is one of the downstream drivers of Wnt/β-catenin signaling pathway [43]. However, it has been reported that c-Myc may be an upstream transcription factor for BZW2 [29, 44]. That means that there may be a positive regulation loop BZW2/GSK3β/β-catenin/c-Myc in LUAD progression here. We will explore this question in the future.

The results of our research contribute to the comprehension of the fundamental function of BZW2 in controlling networks and emphasize the biological and medical foundation for the possible use of BZW2 as a novel indicator for LUAD diagnosis and an effective target for LUAD therapy. To summarize, our investigation validated that the involvement of BZW2 in malignant progression of LUAD. Both in vitro and in vivo, BZW2 stimulated the growth and metastasis of LUAD cells. Furthermore, our research revealed a novel mechanism in which BZW2 triggers the Wnt/β-catenin signaling pathway through promoting the ubiquitination-mediated degradation of GSK3β, ultimately enhancing the progression of LUAD (Fig. 6F). Our research findings enhance the comprehension of the essential role of BZW2 in regulating networks and underscore its significance in both the biological and medical aspects. This highlights the potential of BZW2 as a new marker for diagnosing LUAD and as a promising target for therapeutic interventions.

## Materials and methods

### Data Processing

These gene expression data were downloaded from The Cancer Genome Atlas Program (TCGA, https://tcga-data.nci.nih.gov/tcga/), Genome–Tissue Expression (GTEx, https://www.genome.gov/Funded-Programs-Projects/Genotype-Tissue-Expression-Project) and Gene Expression Omnibus (GEO, https://www.ncbi.nlm.nih.gov/geo/) databases. The protein-protein interaction (PPI) network of BZW2 Homo sapiens for Fig. 4A and S5 was downloaded from BioGRID (https://thebiogrid.org/) and IntAct (https://www.ebi.ac.uk/intact/home) databases, respectively. The above two websites are online bioinformatics analysis databases, and the corresponding results will appear when the gene name is entered.

### Clinical tissue specimen collection

From 2020 to 2023, the Department of Thoracic Surgery, Qilu Hospital of Shandong University (KYLL‐2021[KS]‐1053) acquired 12 sets of recently harvested LUAD tissues along with their corresponding normal lung tissues through surgical resection.

### Tissue microarray, immunohistochemistry (IHC), and hematoxylin/eosin staining (HE)

The tissue microarray (Shanghai Outdo Biotech Co., HLugA180Su00, Shanghai, China) included 87 LUAD tissues, 73 corresponding normal lung tissues and clinical information about these patients. The Ethics Committee of Shanghai Outdo Biotech Company has approved the project (No. SHYJS-CP-1904014). The techniques of IHC and HE staining were carried out according to the previously mentioned protocol [45]. Two pathologists scored the IHC results separately. The standard for IHC score: Hscore (histochemistry score) = staining intensity (0, none; 1, weak; 2, moderate; and 3, strong) × percentage of positive cells (0–100%).

### Cell culture and transfection

There are human bronchial epithelioid cells (Beas-2B), three LUAD cell lines (A549, NCI-H1299 and PC-9) and human embryonic kidney cells (293FT) from the Shanghai Academy of Science (Shanghai, China). STR analysis and mycoplasma testing have been performed. A549 and NCI-H1299 cells were grown in RPMI-1640 medium containing 10% fetal bovine serum (FBS; Gibco, NY, USA). Beas-2B, PC-9, and 293FT cells were grown in high-glucose DMEM containing 10% FBS. A humidified incubator containing 5% CO_2_ at 37 °C was used to incubate these cells. The siRNA including si-NC, si-BZW2 (si-1, si-2) and si-GSK3β (GenePharma, Suzhou, China), oe-NC and oe of BZW2 (Research Cloud Biology, Jinan, China) were transfected into the respective cell. Supplementary Table S1 shows the siRNA sequences.

### Coimmunoprecipitation (co-IP)

The entire cellular lysates were collected and subjected to centrifugation at a speed of 1.2 × 104 rpm for a duration of 15 min at a temperature of 4 °C. Incubation was performed with 1 µg of corresponding antibody separately for each 500 µL supernatant. After incubating for 5 h at a temperature of 4 °C, the mixture was enriched with 50 µL of Protein A/G Magnetic Beads (MedChemExpress, HY-K0202, Shanghai, China) and allowed to incubate overnight at 4 °C. Finally, specimens were gathered and separated through SDS-PAGE before conducting WB examination. Supplementary Table S3 contains a list of the antibodies.

### Immunofluorescence (IF) and confocal microscopy

The PC-9 cells were seeded on coverslips in a 24-well plate. The techniques of IF were carried out according to the previously mentioned protocol [46]. The photographs were taken using a Carl Zeiss LSM980 confocal laser scanning microscope.

### CHX chase assay

Following transfection for 24 h, the cells were incubated with or without MG132 (20 µmol/L) for 6 h. Subsequently, the cells were harvested at specified time intervals after being treated with CHX (100 µg/ml). Preparation of the samples was conducted for the purpose of WB analysis.

### In vivo experiments

20 four-week-old BALB/c nude mice (GemPharmatech Co., Ltd., Nanjing, China) were divided into four groups. Every mouse was implanted with 2 × 10^7^ transfected cells (LV-sh-NC, LV-sh-BZW2, LV-oe-NC and LV-oe-BZW2) at the homolateral armpit area. The tumor volumes were assessed every 6 days. V = (length × width^2^)/2. Following a period of 30 days, the nude mice were euthanized, and the tumors were gathered, measured in weight, and captured in photographs. In the mouse model of pulmonary metastasis, a total of 4 groups were formed by dividing 20 nude mice. 2 × 10^6^ transfected cells mentioned above were implanted into the tail vein of every mouse. Following a period of 8 weeks, the mice without fur were euthanized and their lungs were collected. These samples were stained using either HE or IHC. Approval for the animal experiments (DWLL‐2022‐074) has been granted.

### Statistical analysis

Analysis was conducted using GraphPad Prism 8.0 Software and IBM SPSS Statistics 20.0. The mean ± standard deviation (SD) values are reported based on three separate experiments. *P* values for comparison of two or more groups were calculated using either the student’s *t*-test or ANOVA test. The Kaplan-Meier method was utilized to assess survival curves and compare them using the log-rank test. A statistical significance was determined when the value of *P* < 0.05.

### Supplementary information

Supplementary methods and figure legends

Original Data File

## Data Availability

To obtain the data and material, one can contact the corresponding author and make a reasonable request.
