# Supplementary material for: BZW2 promotes malignant progression in lung adenocarcinoma through enhancing the ubiquitination and degradation of GSK3β
Source: Cell Death Discov. 2024 Feb 29;10:105. doi: 10.1038/s41420-024-01879-7 (PMC10904796; doi:10.1038/s41420-024-01879-7)
Supplement: Supplementary file 1 — Supplementary methods and figure legends [file 41420_2024_1879_MOESM1_ESM.pdf]

Supplementary Materials and Methods

Data Processing

These gene expression data were downloaded from Gene Expression Omnibus (GEO, https://www.ncbi.nlm.nih.gov/geo/) databases and IntAct (https://www.ebi.ac.uk/intact/home) databases.

RNA isolation, cDNA preparation, and real-time quantitative polymerase chain reaction

Total RNA was extracted by the RNAfast200 kit (Fastagen, 220011, Shanghai, China). Complementary DNA (cDNA) was synthesised using a reverse transcription kit (Toyobo, Osaka, Japan) according to the manufacturer's instructions. Real-time quantitative-PCR (qRT-PCR) using the SYBR Green Supermix (Toyobo, Osaka, Japan) was executed in Bio-Rad IQ 5 system (Bio-Rad, California, America). The primer sequences used for qRT-PCR are listed in Supplementary Table S2.

Western blotting assay

The radioimmunoprecipitation assay (RIPA) reagent (Beyotime, P0013, Shanghai, China) with Protease and Phosphatase Inhibitor Cocktail (NCM Biotech, P002, Suzhou) was used to extracte the total proteins of LUAD tissues or cells. The concentration of protein was quantified by a BCA kit (Beyotime, P0010, Shanghai, China). Subsequently, the protein samples were separated using 10% sodium dodecyl sulfate-polyacrylamide gel electrophoresis (SDS-PAGE) gels and transferred to 0.22μm polyvinylidene

difluoride (PVDF) membranes (Millipore, Billerica, Massachusetts, USA). Then we used 5% non-fat milk to block the membranes at room temperature for 1 h and subsequently incubated the PVDF membranes with primary antibodies at 4°C overnight after washed by TBST (thrice for 10 min each). The second day, a super chemiluminescence (ECL) kit (Yeasen, 36222ES, Shanghai, China) was used to visualize the target protein after the secondary antibody for 1 h at room temperature. The antibodies are listed in Supplementary Table S3.

Cell Counting Kit-8 Cell Proliferation Assay

After transfected for 24 hours, the cells were seeded into 96-well plates (2×10^3cells/well). At indicated time after incubation (24, 48, 72, and 96 h), 10 μL of CCK-8 solution (APExBIO, #K1018, Houston, America) was added to each well. The absorbance was recorded at 450 nm using a Microplate Reader (Bio-Rad, Hercules, California, USA) after incubated for 2 h in dark.

EdU incorporation assay

The cells that transfected for 24 hours were seeded into 96-well plates (8×10^3 cells/well). After the cells were cultured to the normal growth stage, the medium in the 96-well plate was replaced with fresh medium containing 5-ethynyl-2-′deoxyuridine (EdU; 1000:1) for 2 h. The measurement was performed using EdU incorporation assay kit (RiboBio, C10310-1, Guangzhou, China) according to the manufacturer’s protocol. A fluorescence microscope (Nikon, Japan) was used to photograph the stained cells.

Flow cytometry analysis

A Cell Cycle Staining Kit (Multi Sciences, C10310-1, Hangzhou, China) was used to detect the cell cycle distribution of the cells that transfected for 24 hours through a flow cytometer according to the manufacturer's protocol. ModFit software was used to determine the percentage of cells in various phases of the cell cycle.

Wound healing assay

The cells that transfected for 24 hours were seeded into 6-well plates. When the confluence of these cells reaches to 90%, the tip of a 200-μL pipette was used to scratch the cell monolayer. After washed with PBS, the cells were cultured with serum-free media. The width of the wound was visualised under a light microscope at the indicated time.

Transwell assay

The upper Transwell chamber with or without Matrigel was seeded with the cells (3×10^4 cells/well) that transfected for 24 hours and the lower Transwell chamber was added with 700 μL of a medium supplemented with 20% FBS. After 24 h, the cells in the upper chamber moved to the lower chamber and were fixed with methanol for 30 min. Subsequently, the cells were stained with 0.2% crystal violet for 20 min and were photographed by a light microscope.

Lentivirus production and infection

The sh-NC, sh-BZW2, oe-NC and oe-BZW2 lentiviruses (Syngentech, Beijing, China) were transfected into the corresponding cells. The sh-RNA sequences are listed in Supplementary Table S1.

Data processing and transcriptome sequencing

Transcriptome sequencing (RNA-seq) was performed by Lianchuan Biotechnology using the Illumina HiSeq4000 platform. The samples were PC-9 cells transfected with siRNA including si-NC and si-1. We further performed computational simulation by using Ingenuity Pathway Analysis (IPA; QIAGEN, Valencia, CA, USA) online tools to canonical pathways.

Supplementary Table S1. siRNA and sh-RNA used for transfection.

Name	Sense (5’-3’)	Antisense (3’-5’)
si-BZW2		
si-1	GGAGAAGGCAUUUGAAGAUTT	AUCUUCAAAUGCCUUCUCCTT
si-2	GGAGCUCCAGGAGCGUCUUTT	AAGACGCUCCUGGAGCUCCTT
si-GSK3β	GACGCUCCCUGUGAUUUAUTT	AUAAAUCACAGGGAGCGUCTT
si-NC	UUCUCCGAACGUGUCACGUTT	ACGUGACACGUUCGGAGAATT
sh-BZW2	GGAGAAGGCAUUUGAAGAUTT	AUCUUCAAAUGCCUUCUCCTT
sh-NC	UUCUCCGAACGUGUCACGUTT	ACGUGACACGUUCGGAGAATT

Supplementary Table S2. Primers used for qRT-PCR.

Gene	Forward (5’-3’)	Reverse (3’-5’)
BZW2	GAATGCCCGATCAAGGAGGT	TCTTGTTCCACTCAACAGCGT
GSK3β	TCACAGGGAGCGTCTGTTTG	TGGTGCTGGACTATGTTCCG
GAPDH	GCACCGTCAAGGCTGAGAAC	TGGTGAAGACGCCAGTGGA

Supplementary Table S3. Antibodies used in the experiments.

Antigen	Supplier	Catalog #	Application
GAPDH	Huabio	ET1601-4	IB (1:20,000)
BZW2	GeneTex	GTX106985	IB (1:1,000)<br>IF (1:100)<br>IHC (1:100)
BZW2	Thermo Fisher	A304-608A	IP (1 μg/500 μg lysate)
Ki67	Servicebio	GB111499-100	IHC (1:500)
PCNA	Servicebio	GB11010-100	IHC (1:200)
IgG	Cell Signaling Technology	3900	IP (1 μg/500 μg lysate)
GSK3β	Cell Signaling Technology	12456	IB (1:1,000)<br>IF (1:200)<br>IP (1 μg/500 μg lysate)
Myc-Tag (Rabbit)	Huabio	R1208-1	IB (1:2,000)<br>IP (1 μg/500 μg lysate)
Myc-Tag (Mouse)	Huabio	EM31105	IB (1:2,000)<br>IP (1 μg/500 μg lysate)
Flag-Tag (Rabbit)	Huabio	0912-1	IB (1:5,000)<br>IP (1 μg/500 μg lysate)
Flag-Tag (Mouse)	Huabio	HA601080	IB (1:5,000)<br>IP (1 μg/500 μg lysate)
p-GSK3β (Ser 9)	Huabio	ET1607-60	IB (1:2,000)

Ubiquitin	Cell Signaling Technology	3936	IB (1:1,000)
TRAF6	Cell Signaling Technology	8028	IB (1:1,000)
N-Cadherin	Proteintech	22018-1-AP	IB (1:2,000)
E-Cadherin	Abways	CY1155	IB (1:5,000)
β-catenin	Huabio	0407-16	IB (1:1,000)
c-Myc	Abways	CY5150	IB (1:1,000)
Vimentin	Huabio	R1308-6	IB (1:1,000)
c-Jun	Huabio	ET1608-3	IB (1:1,000)
Cyclin D1	Huabio	ET1601-31	IB (1:1,000)
Snail	Proteintech	13099-1-AP	IB (1:1,000)
Slug	Huabio	EM1706-65	IB (1:1,000)
HRP-anti- rabbit	Huabio	HA1001	IB (1:50,000)
HRP-anti- mouse	Huabio	HA1006	IB (1:20,000)
IPKine™ HRP, Mouse Anti-Rabbit IgG LCS	Abbkine	A25022	IB (1:2,000)

Supplementary Figures and Figure Legends

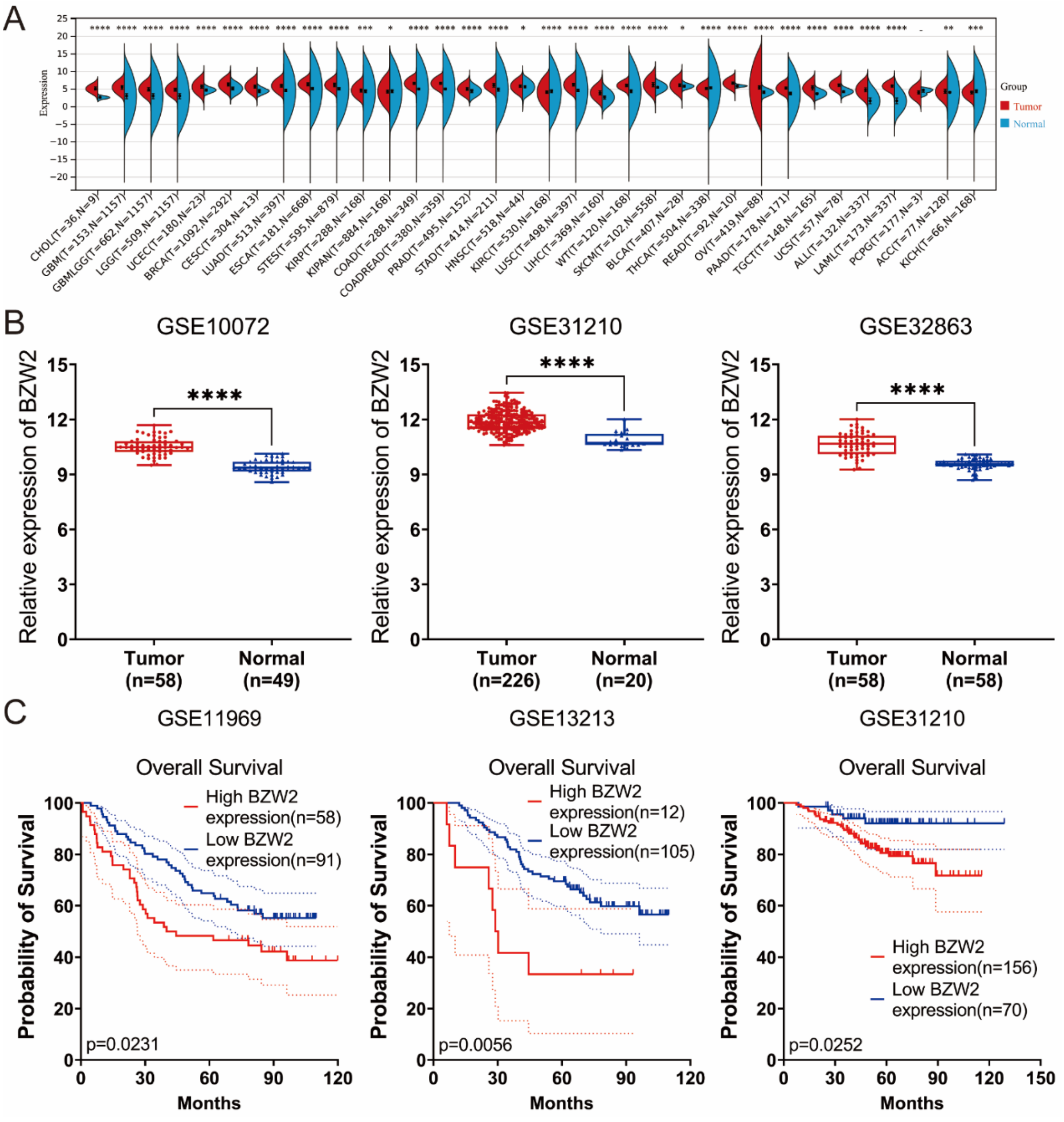

Figure S1. The mRNA expression of BZW2 was upregulated and was correlated with a poor prognosis in LUAD patients. **A** The mRNA levels of BZW2 were upregulated in a variety of cancers. **B** The mRNA expression of BZW2 was higher in tumor tissues than in normal lung tissues according to GEO database. **C** Kaplan–Meier analysis for LUAD samples based on the expression of BZW2 according to GEO database. (ns, no significance, *P < 0.05, **P < 0.01, ***P < 0.001, ****P < 0.0001)

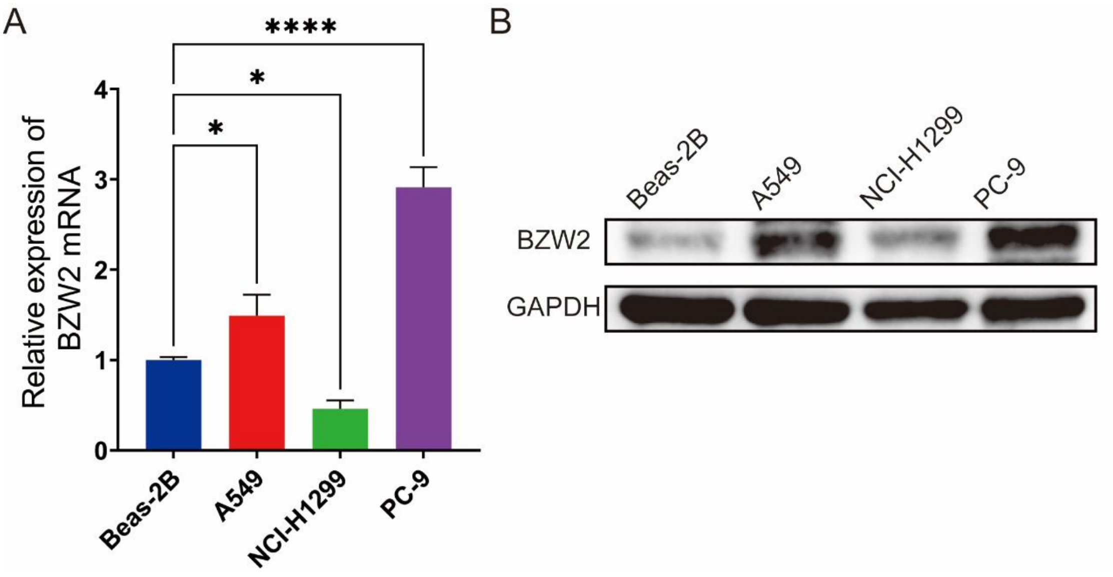

Figure S2. The expression of BZW2 in LUAD cell lines and bronchial epithelial cells. The expression levels of BZW2 in Beas-2B cells and several LUAD cell lines, such as A549, NCI-H1299 and PC-9 were confirmed by qRT-PCR (**A**) and western blot (**B**). (*P < 0.05, ****P < 0.0001)

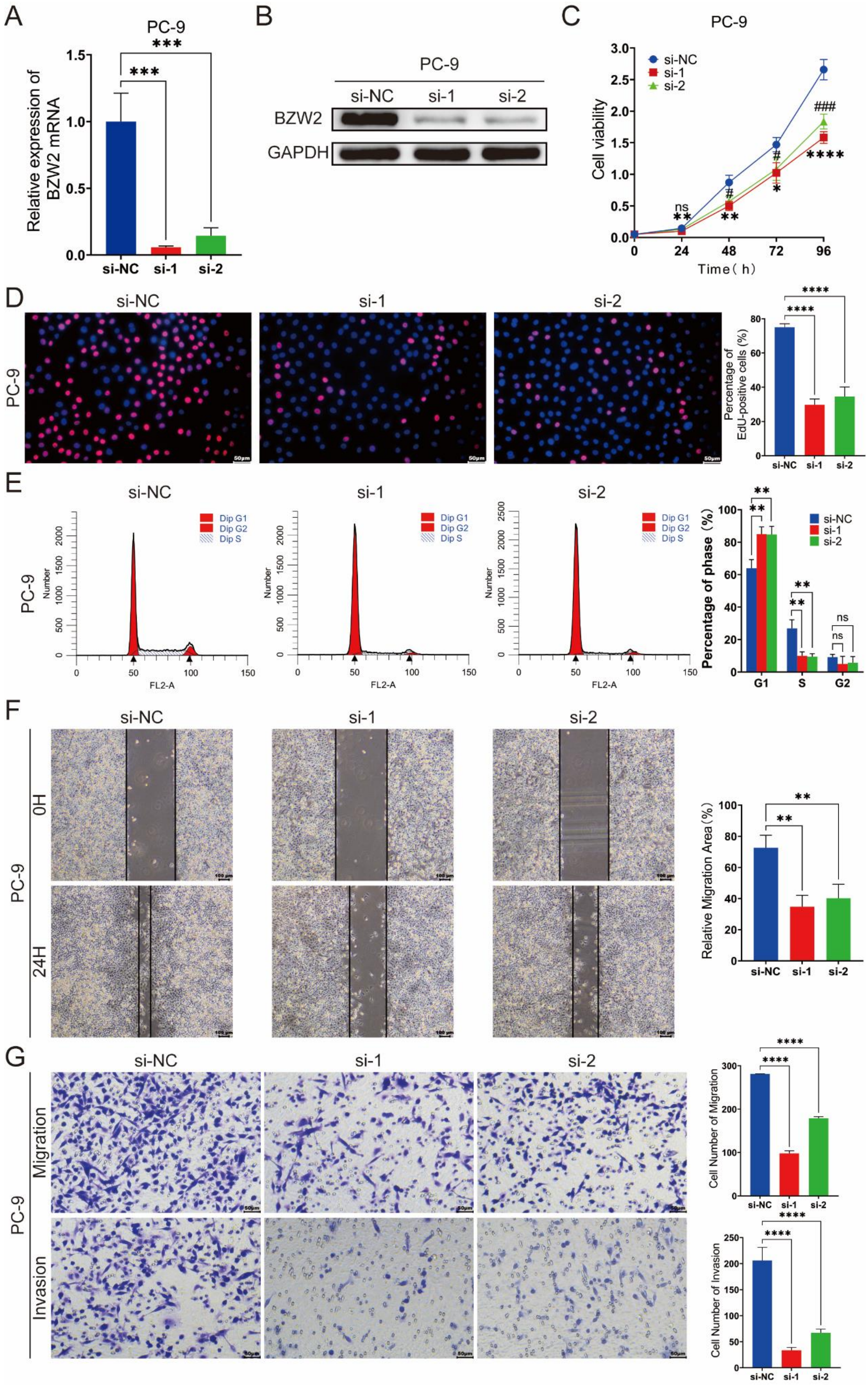
A
PC-9
Relative expression of BZW2 mRNA
si-NC
si-1
si-2
B
PC-9
BZW2
GAPDH
C
PC-9
Cell viability
si-NC
si-1
si-2
Time（h）
ns
D
PC-9
Percentage of EdU-positive cells (%)
E
Dip G1
Dip G2
Dip S
Number
FL2-A
Percentage of phase（%）
G1
S
G2
F
0H
24H
Relative Migration Area (%)
G
Migration
Invasion
Cell Number of Migration
Cell Number of Invasion

Figure S3. The knockdown of BZW2 inhibited the proliferation, migration and invasion of LUAD *in vitro.* The knockdown efficiencies of BZW2 were confirmed by qRT-PCR (**A**) and western blot (**B**) in LUAD cells. CCK8 (**C**), EdU incorporation assays (**D**) and flow cytometry analyses (**E**) detected the proliferation ability in BZW2-depleted PC-9 cells and control cells. Wound healing assays (**F**) and Transwell assays (**G**) detected the migration and invasion abilities in BZW2-depleted PC-9 cells and control cells. (ns, no significance, *P < 0.05, **P < 0.01, ***P < 0.001, ****P < 0.0001).

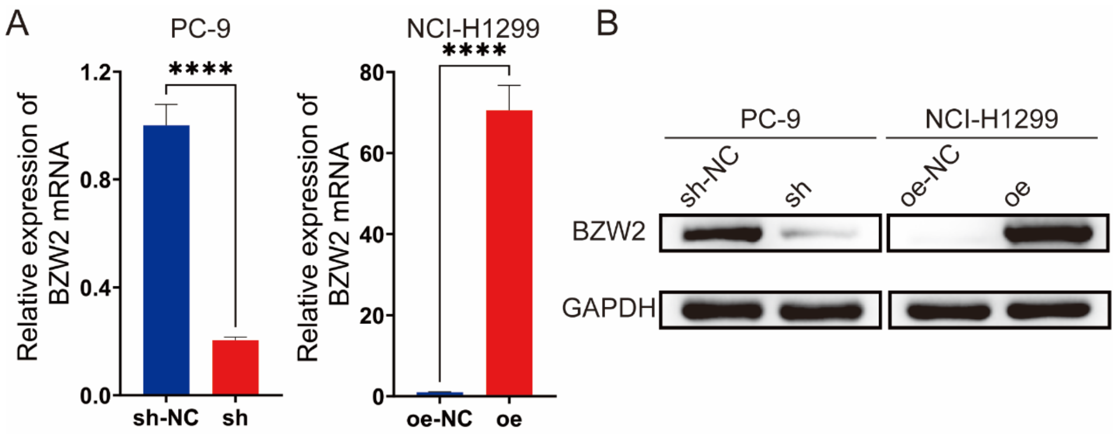

Figure S4. The lentiviral transfection efficiencies of BZW2 in LUAD cells. The qRT-PCR (**A**) and western blot (**B**) evaluated the lentiviral transfection efficiencies of BZW2 in LUAD cells. (****P < 0.0001).

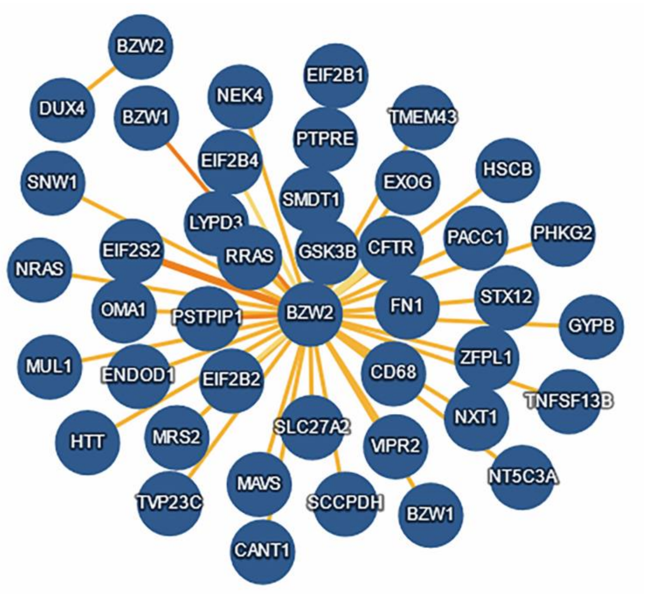

Figure S5. BZW2 interacts with GSK3β. **A** The PPI network was obtained from the IntAct database.

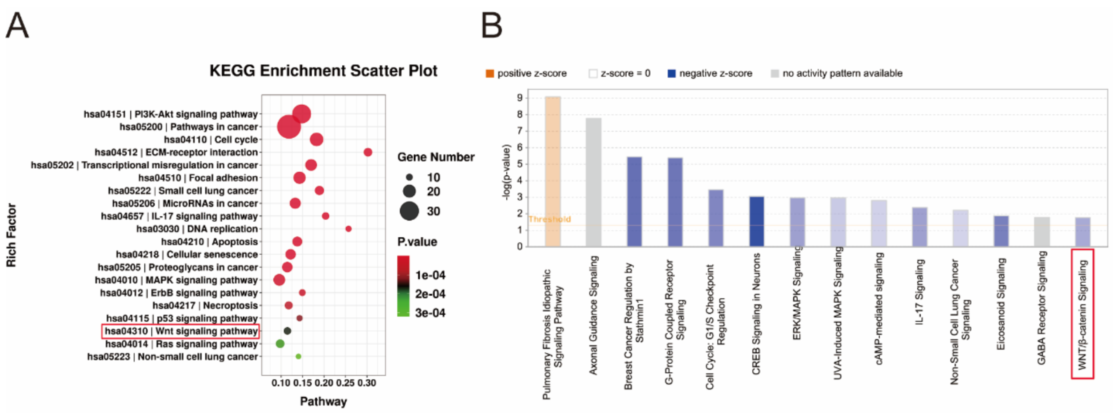

Figure S6. BZW2 activated Wnt/β-catenin signaling pathway in LUAD cells. **A** Kyoto Encyclopedia of Genes and Genomes (KEGG) pathway enrichment analysis of the transcriptome sequencing data. **B** Ingenuity Pathway Analysis (IPA) of the transcriptome sequencing data.
