# Supplementary material for: BZW2 promotes malignant progression in lung adenocarcinoma through enhancing the ubiquitination and degradation of GSK3β
Source: Cell Death Discov. 2024 Feb 29;10:105. doi: 10.1038/s41420-024-01879-7 (PMC10904796; doi:10.1038/s41420-024-01879-7)
Supplement: Supplementary file 2 — Original Data File [file 41420_2024_1879_MOESM2_ESM.ppt]

## Slide 1
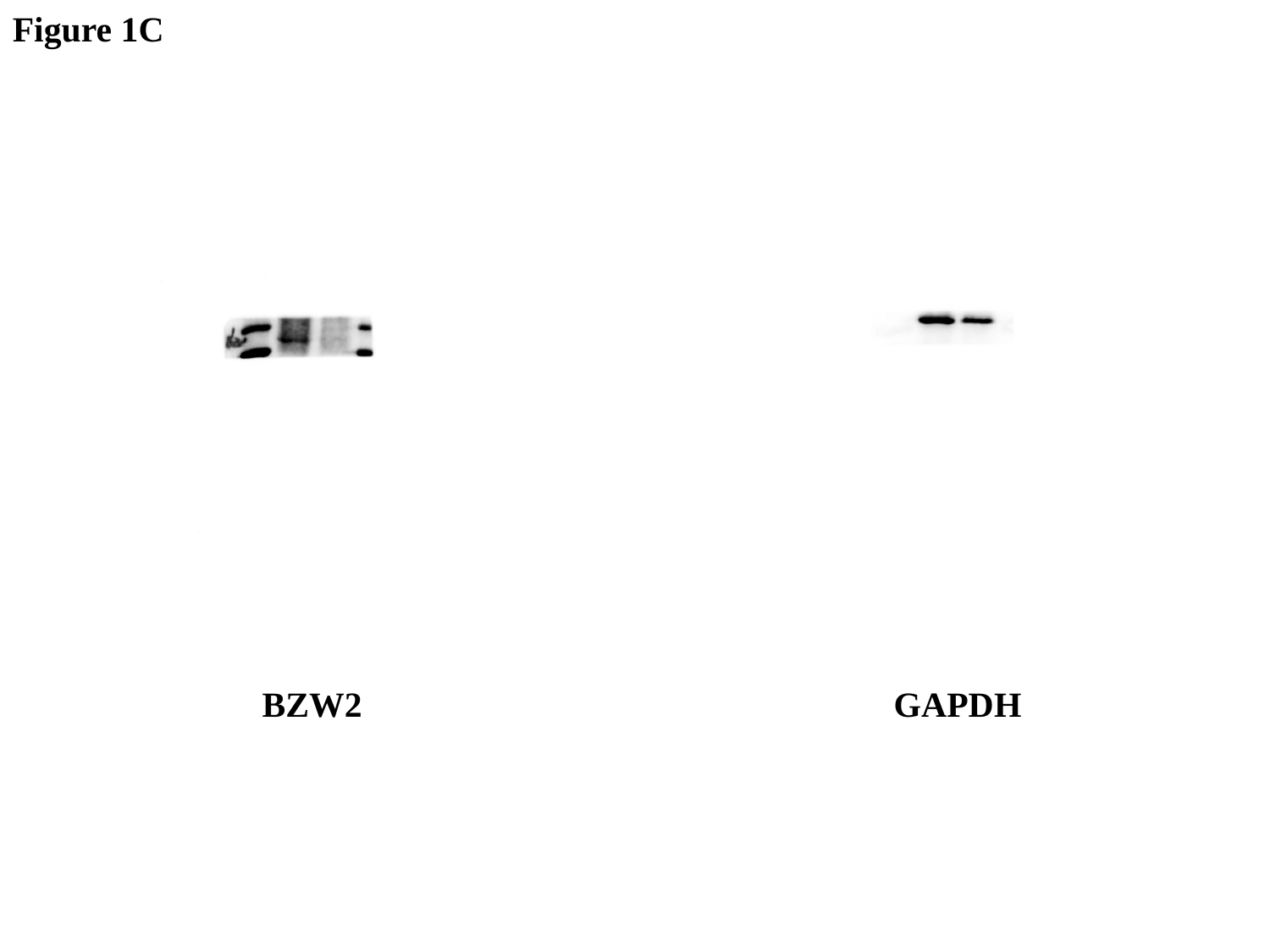

Figure 1C
GAPDH
BZW2

## Slide 2
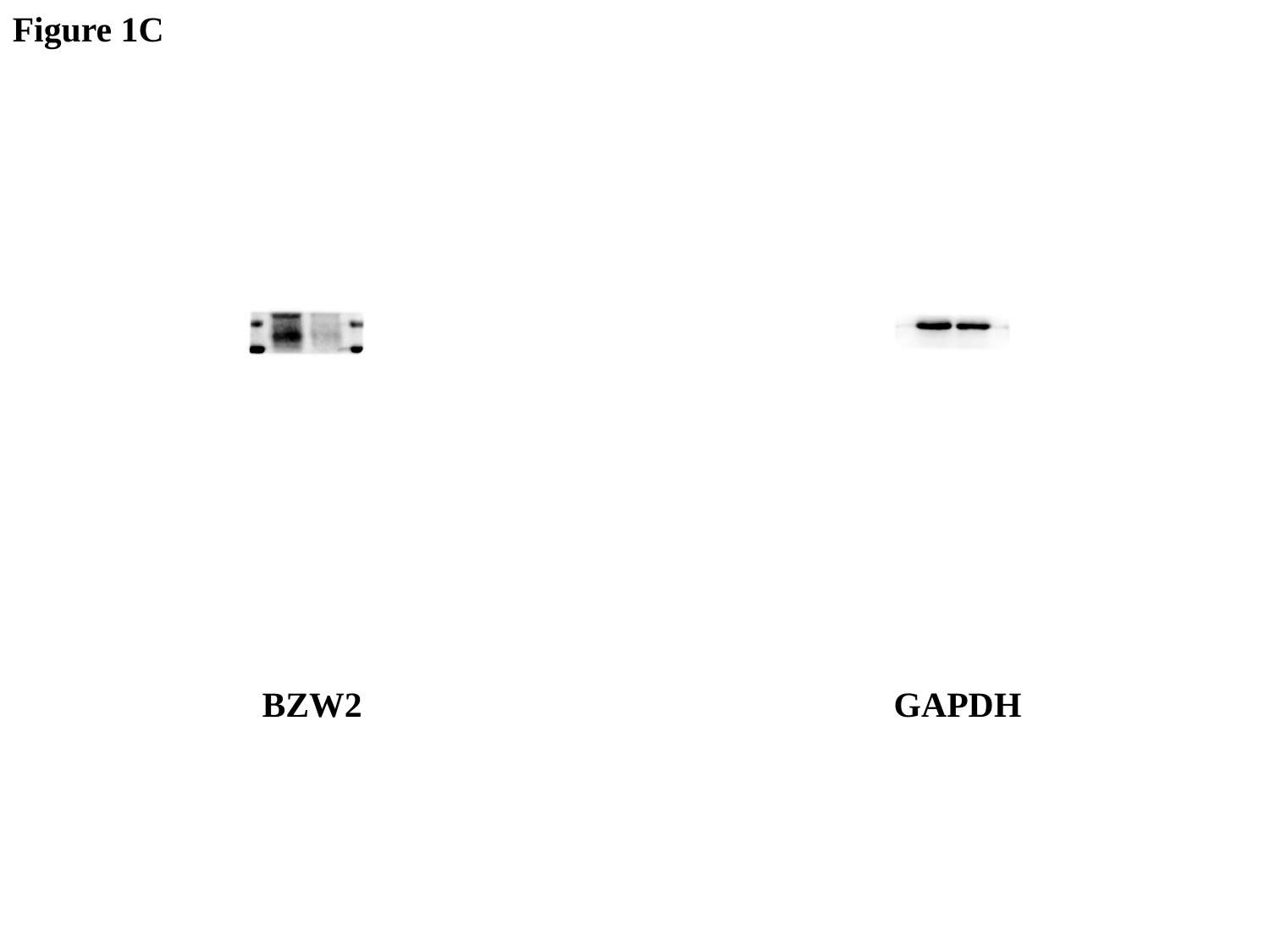

Figure 1C
GAPDH
BZW2

## Slide 3
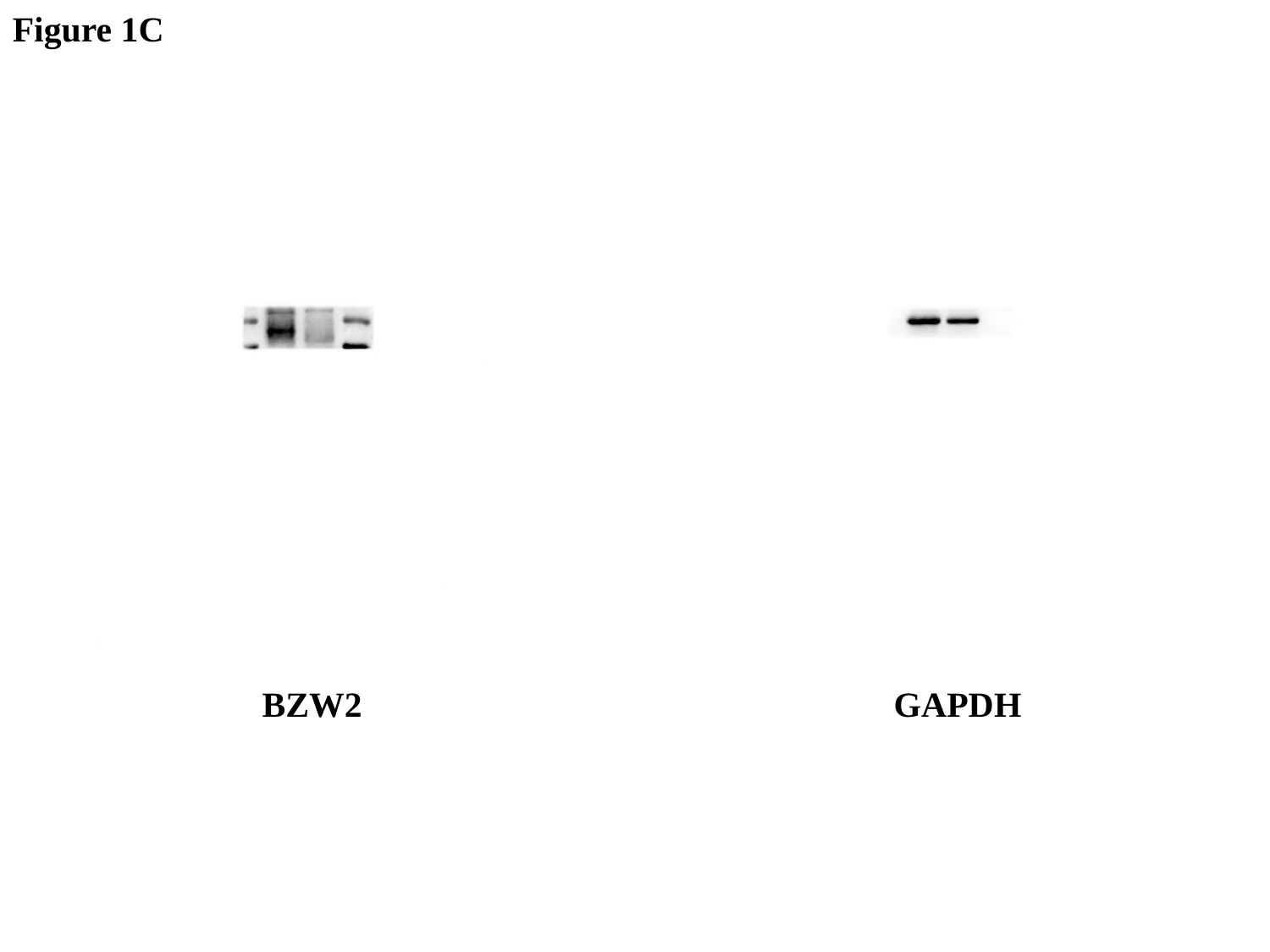

Figure 1C
GAPDH
BZW2

## Slide 4
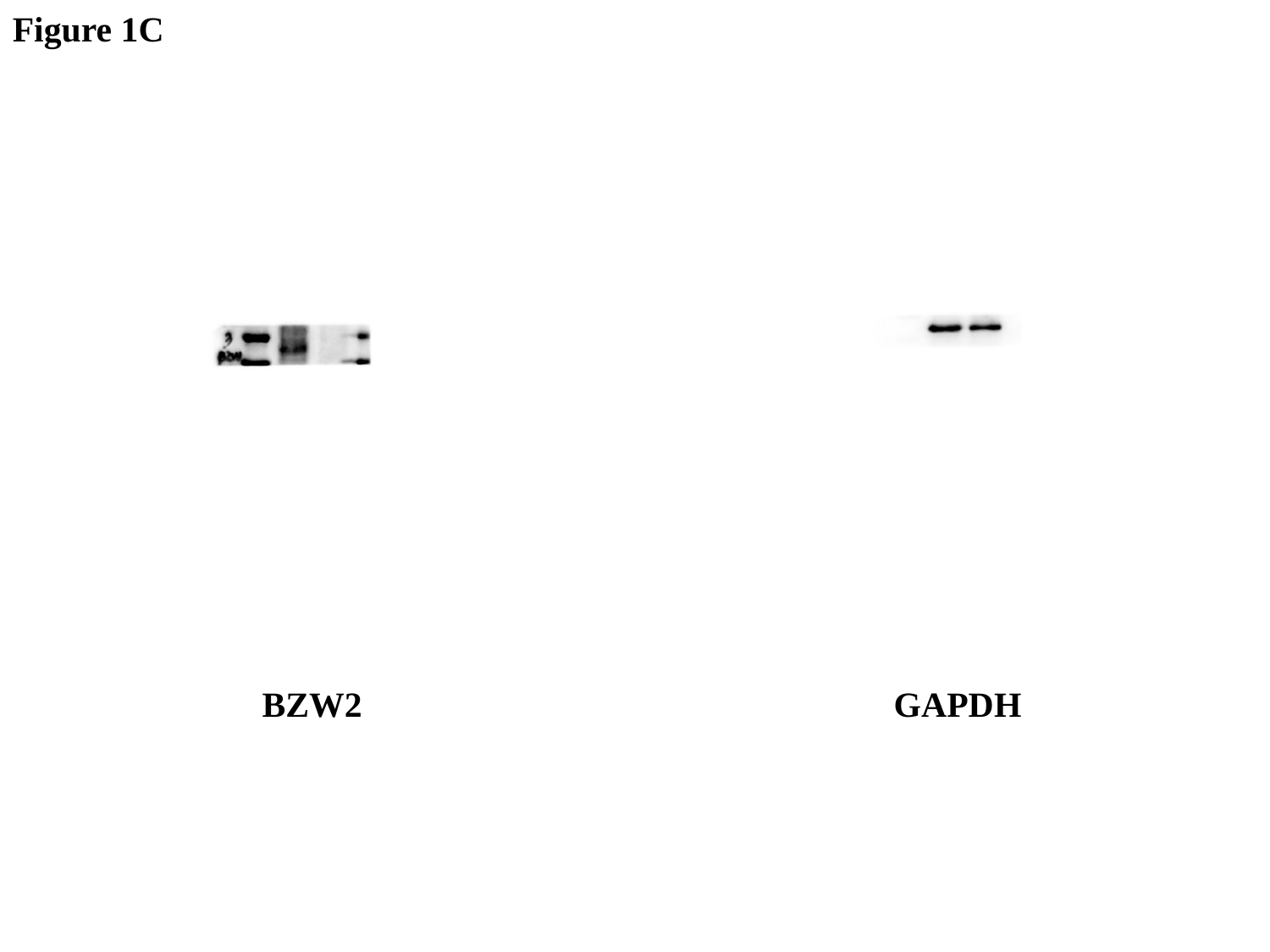

Figure 1C
GAPDH
BZW2

## Slide 5
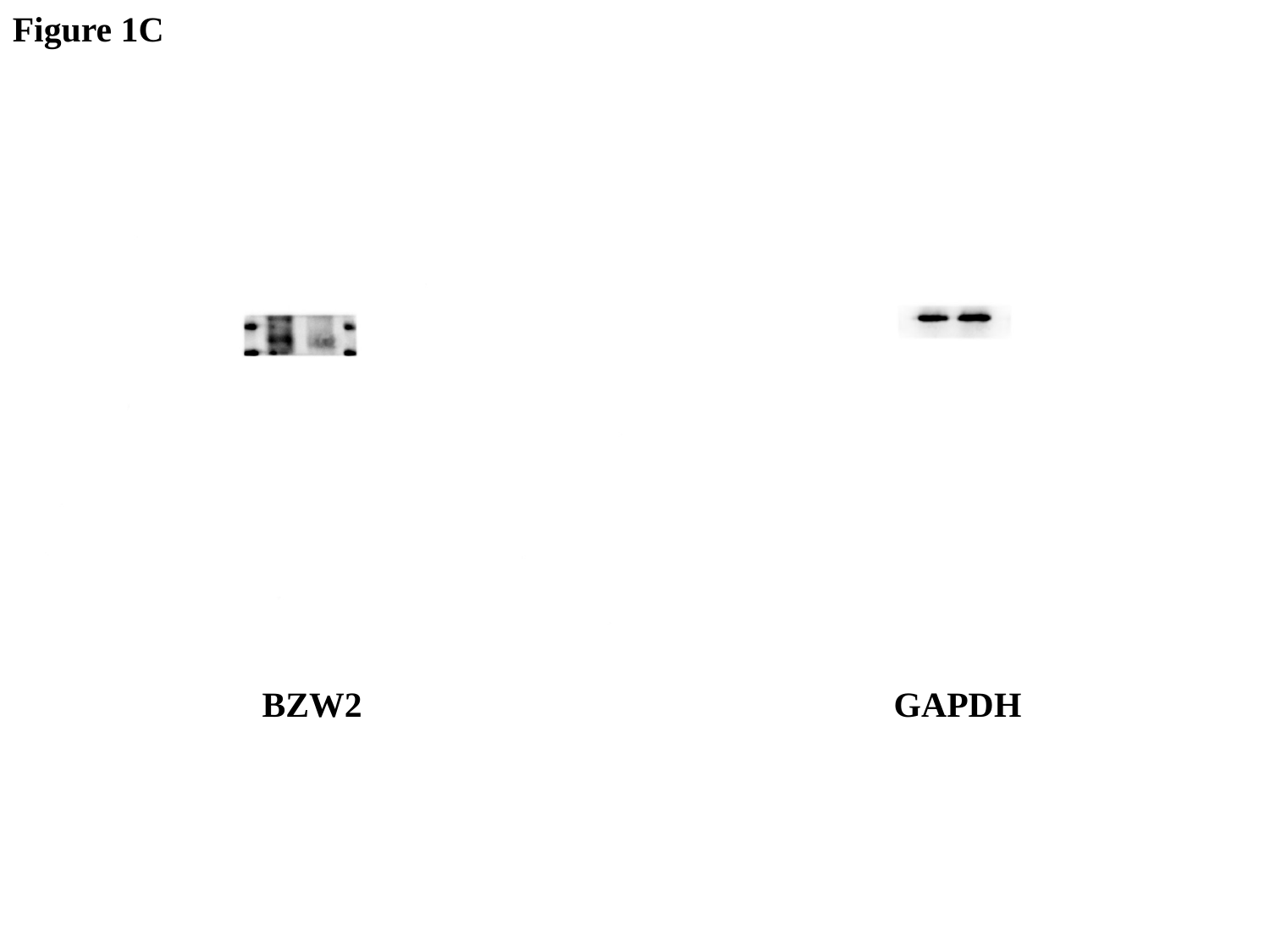

Figure 1C
GAPDH
BZW2

## Slide 6
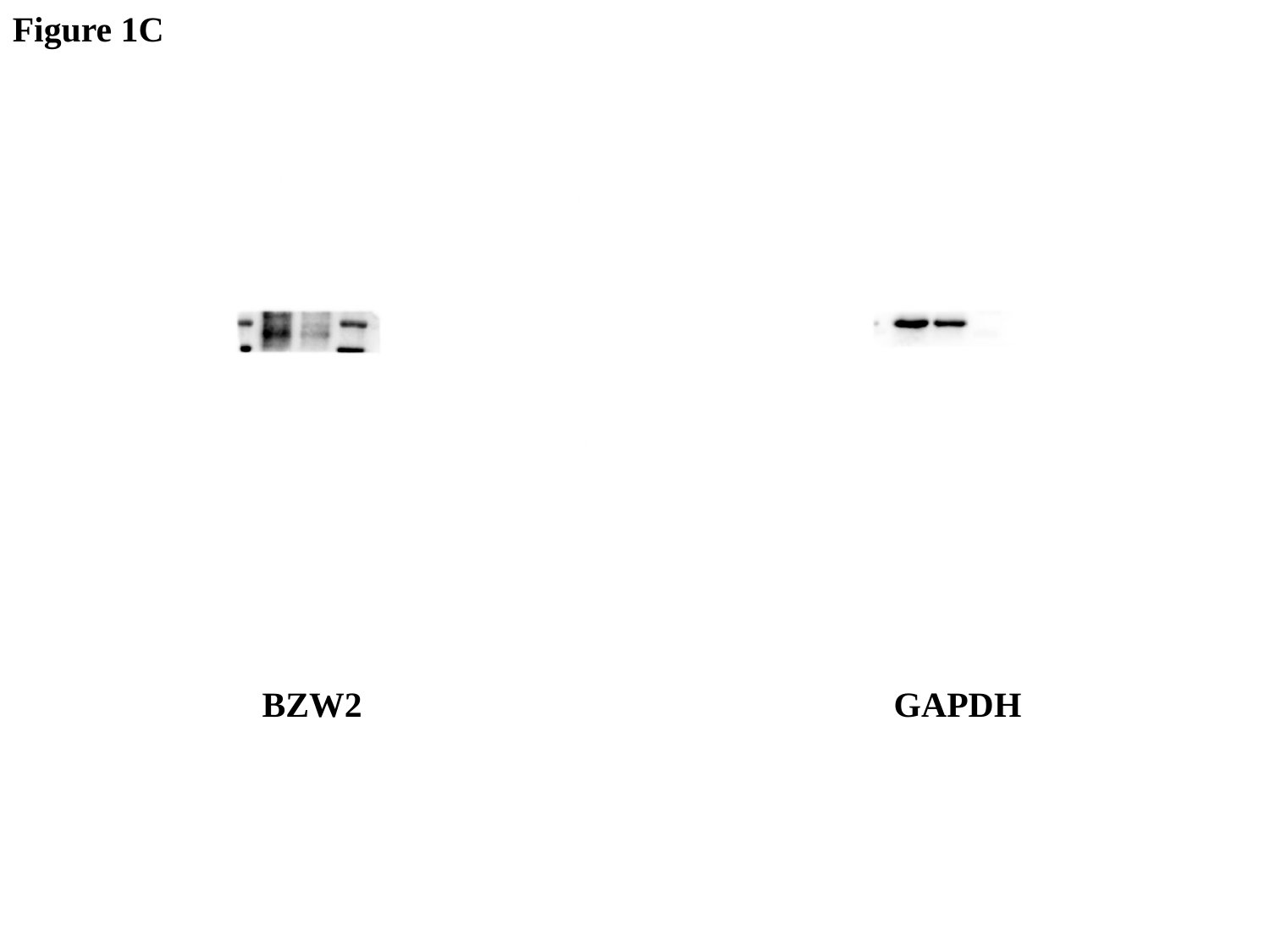

Figure 1C
GAPDH
BZW2

## Slide 7
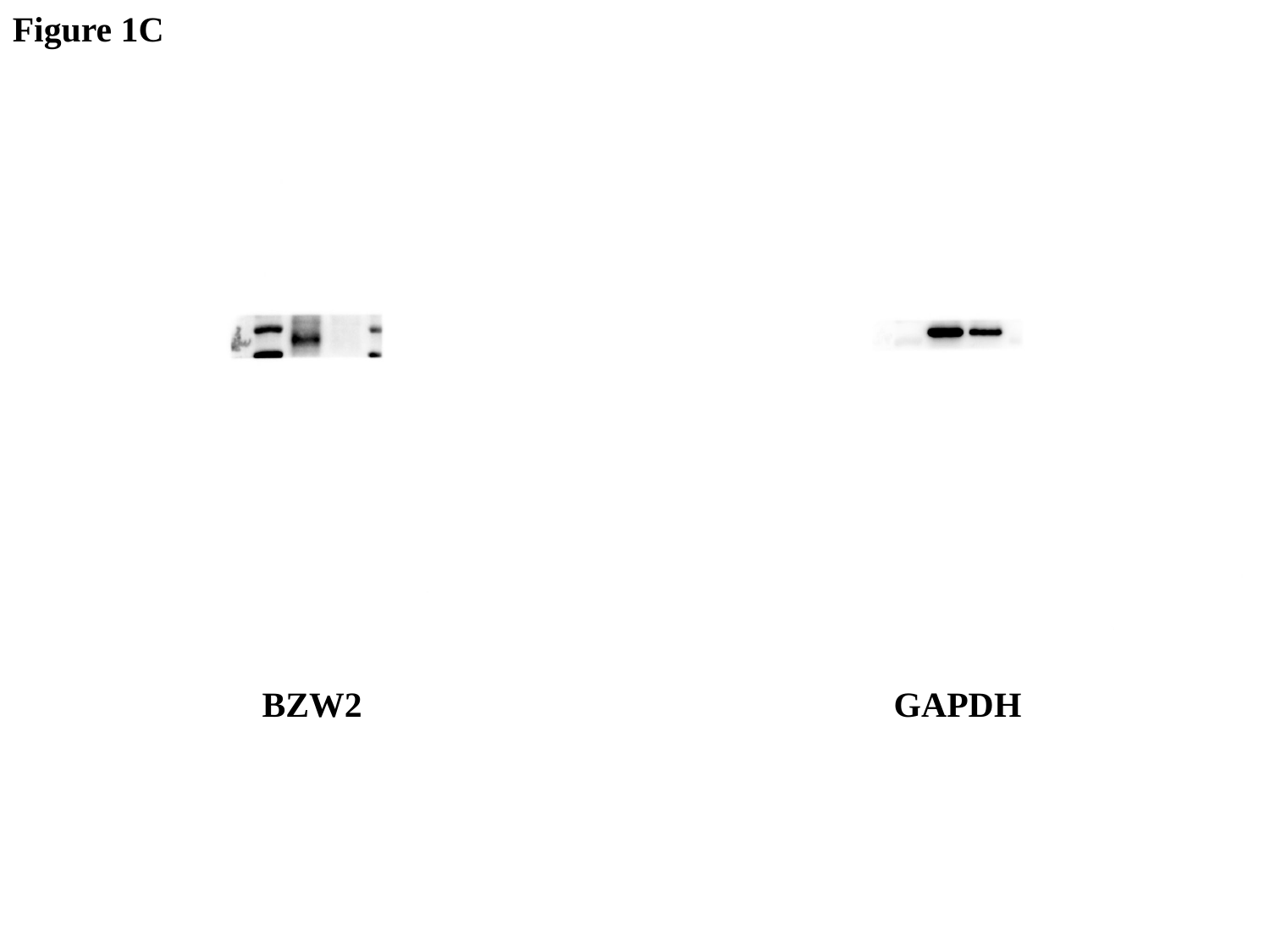

Figure 1C
GAPDH
BZW2

## Slide 8
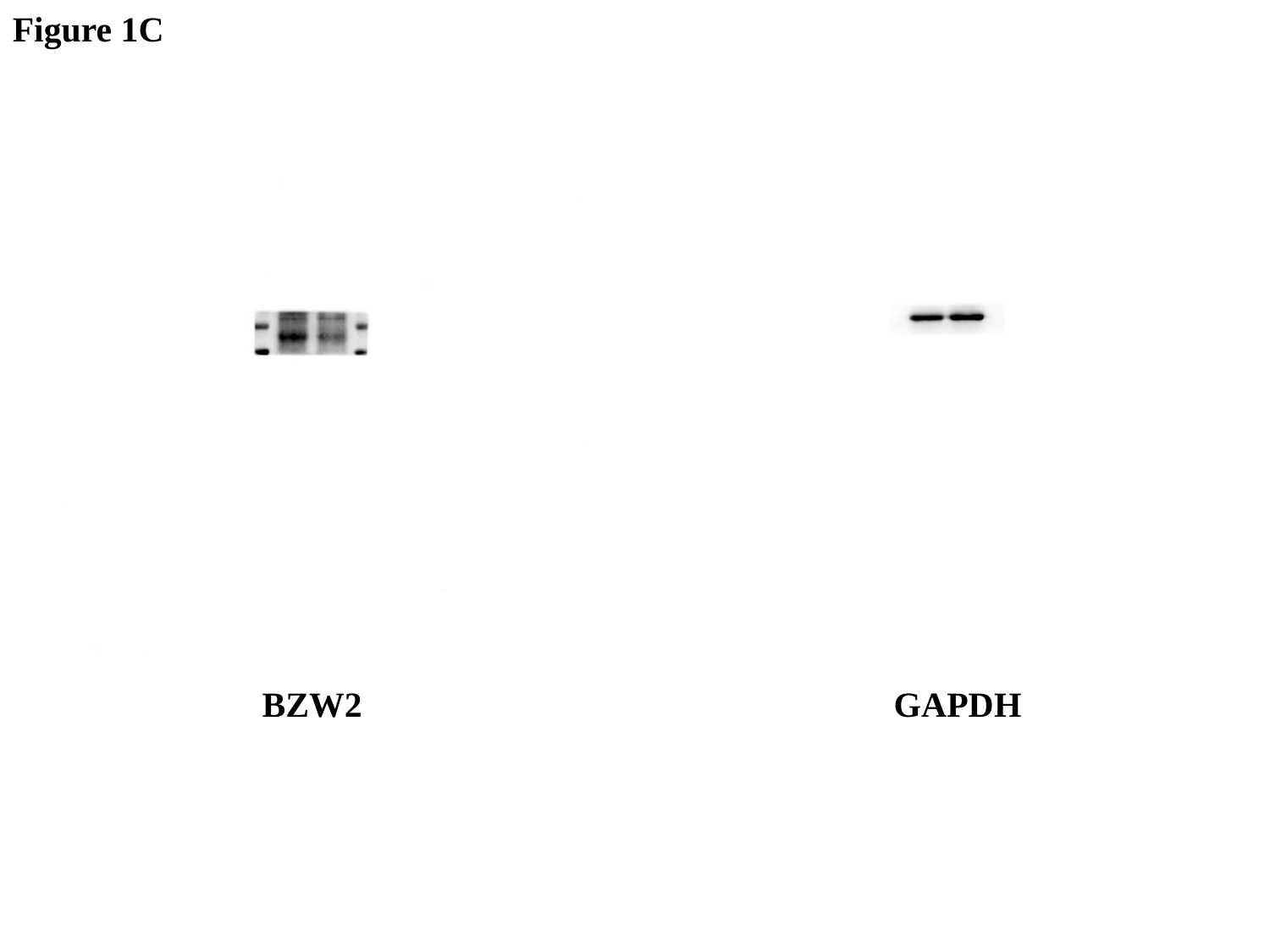

Figure 1C
GAPDH
BZW2

## Slide 9
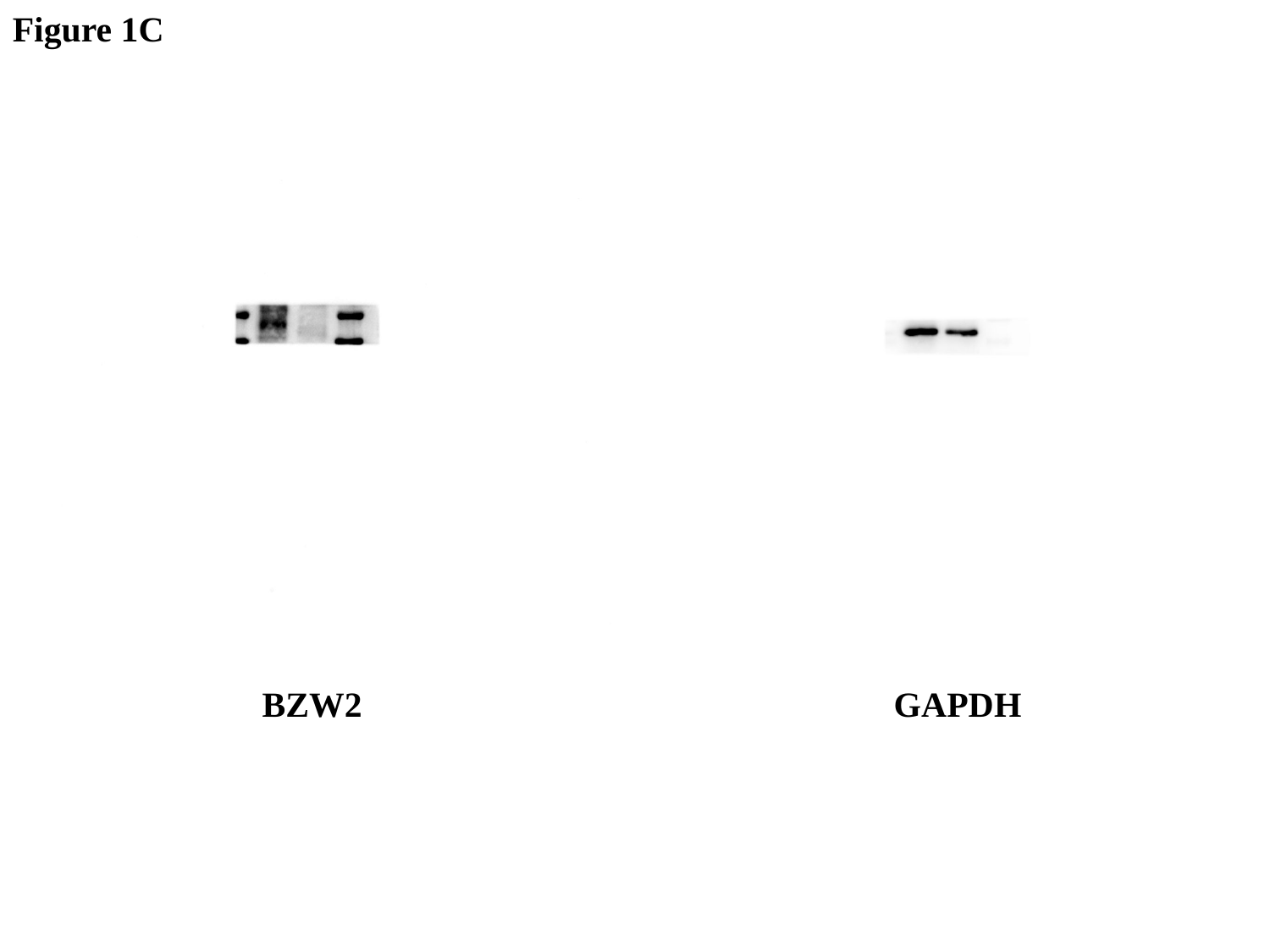

Figure 1C
GAPDH
BZW2

## Slide 10
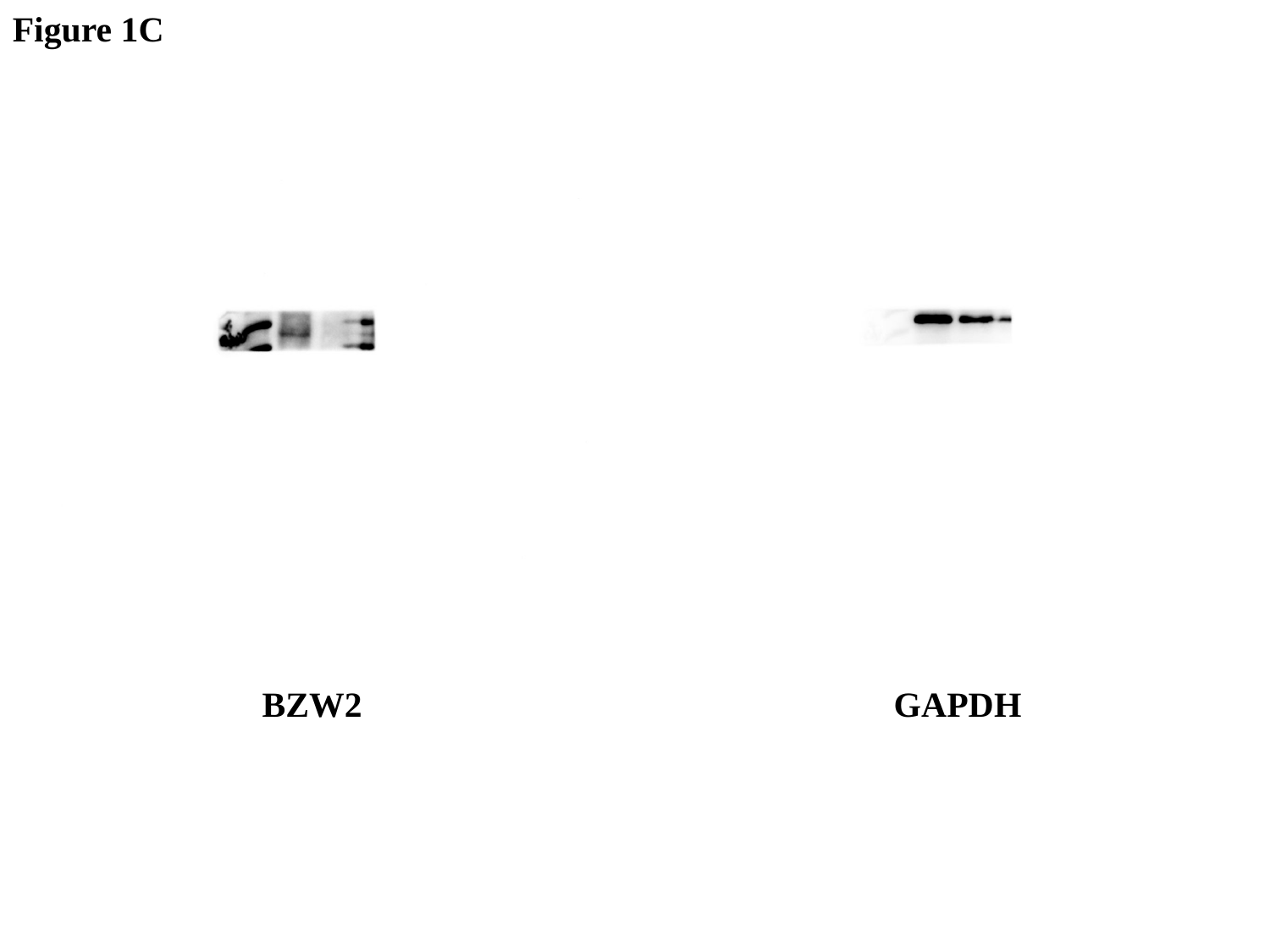

Figure 1C
GAPDH
BZW2

## Slide 11
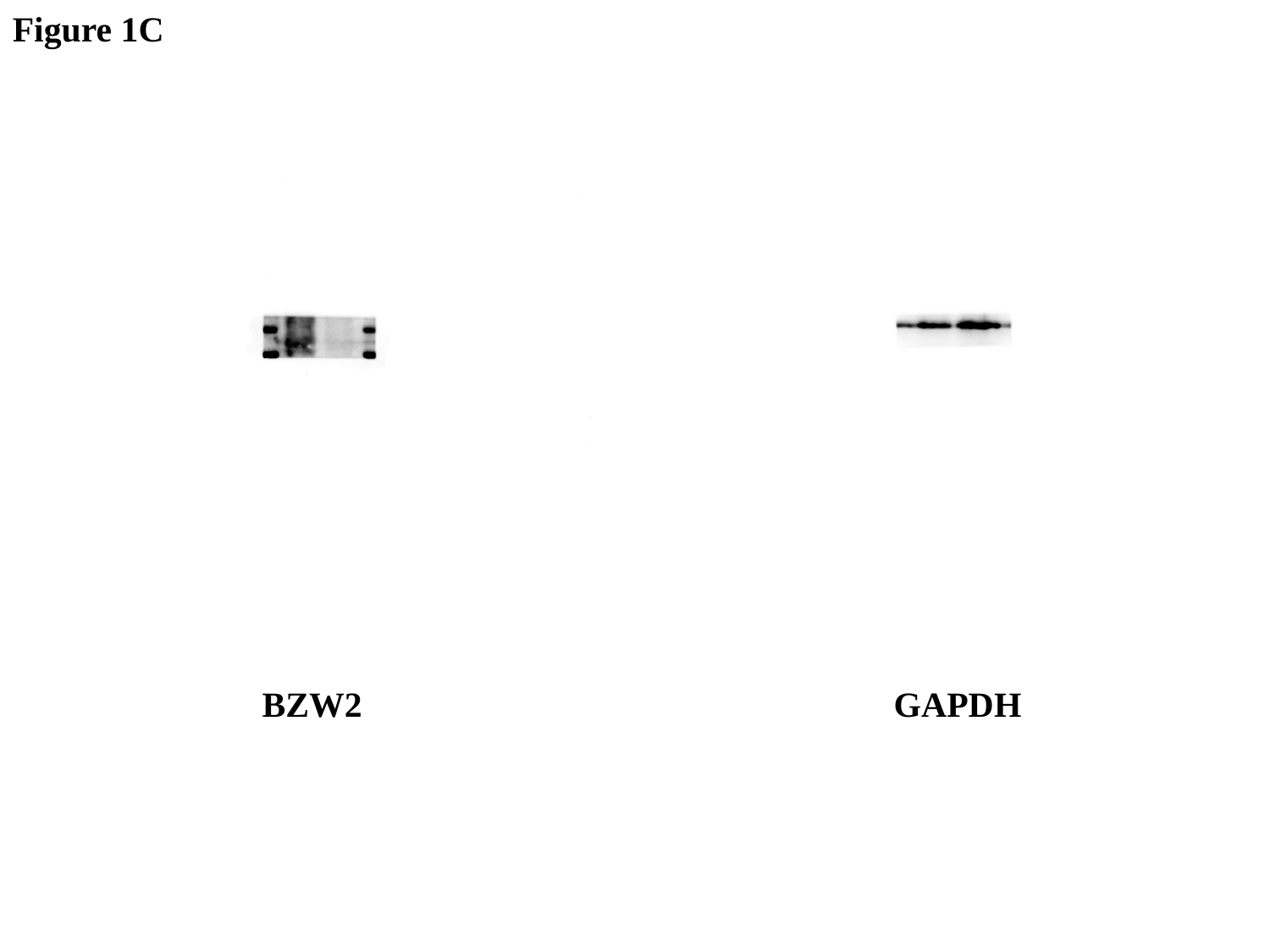

Figure 1C
GAPDH
BZW2

## Slide 12
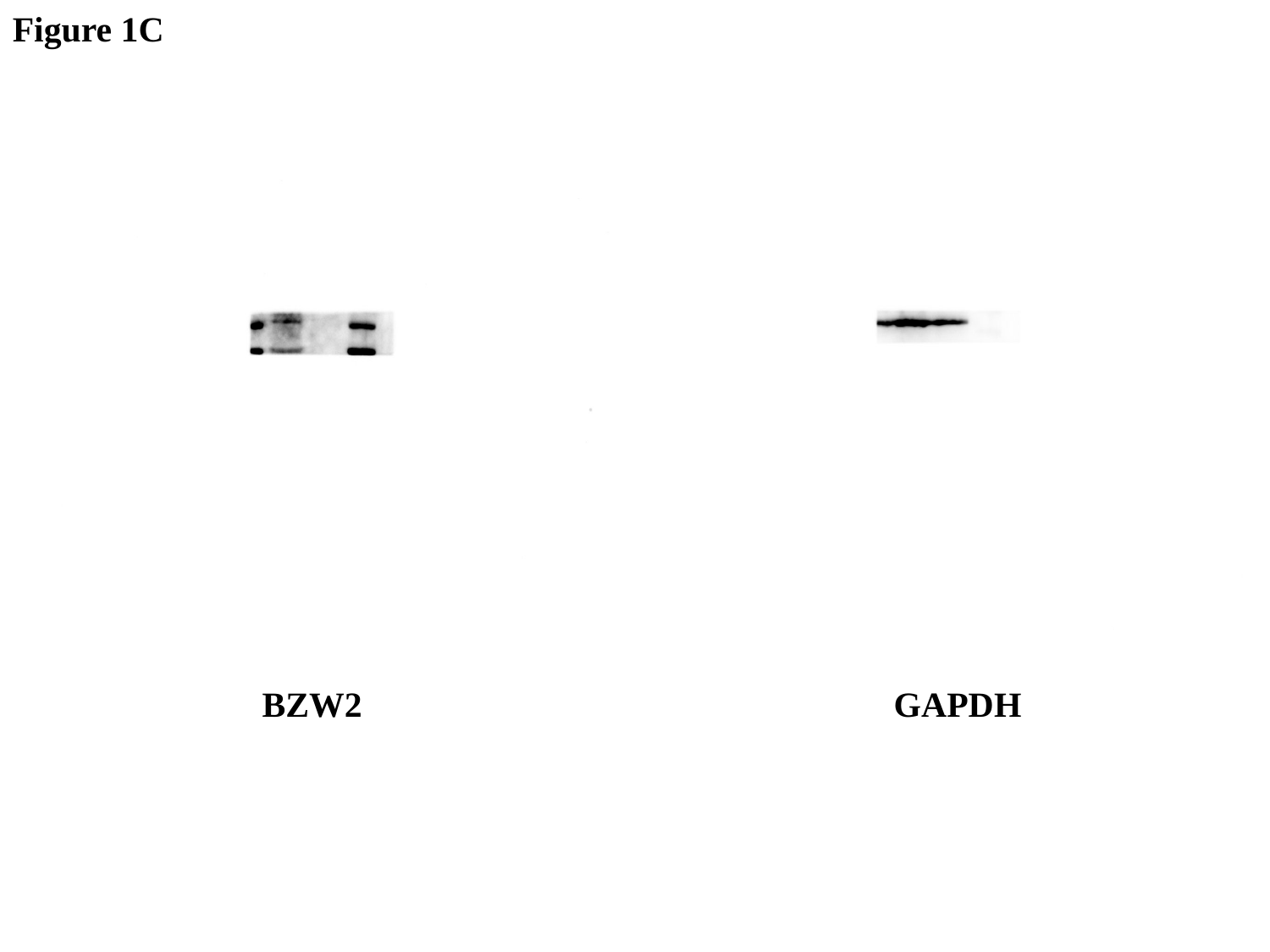

Figure 1C
GAPDH
BZW2

## Slide 13
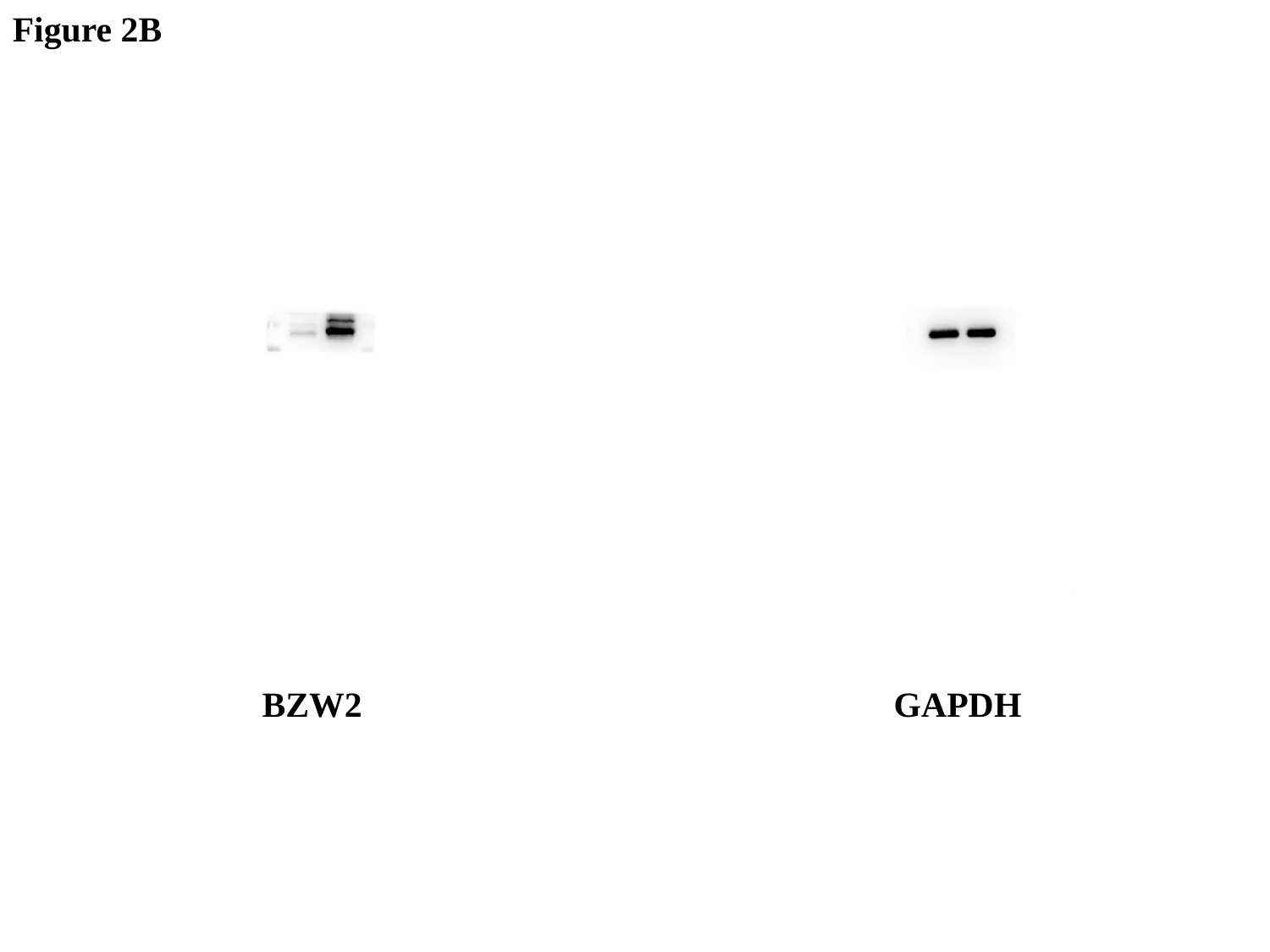

Figure 2B
GAPDH
BZW2

## Slide 14
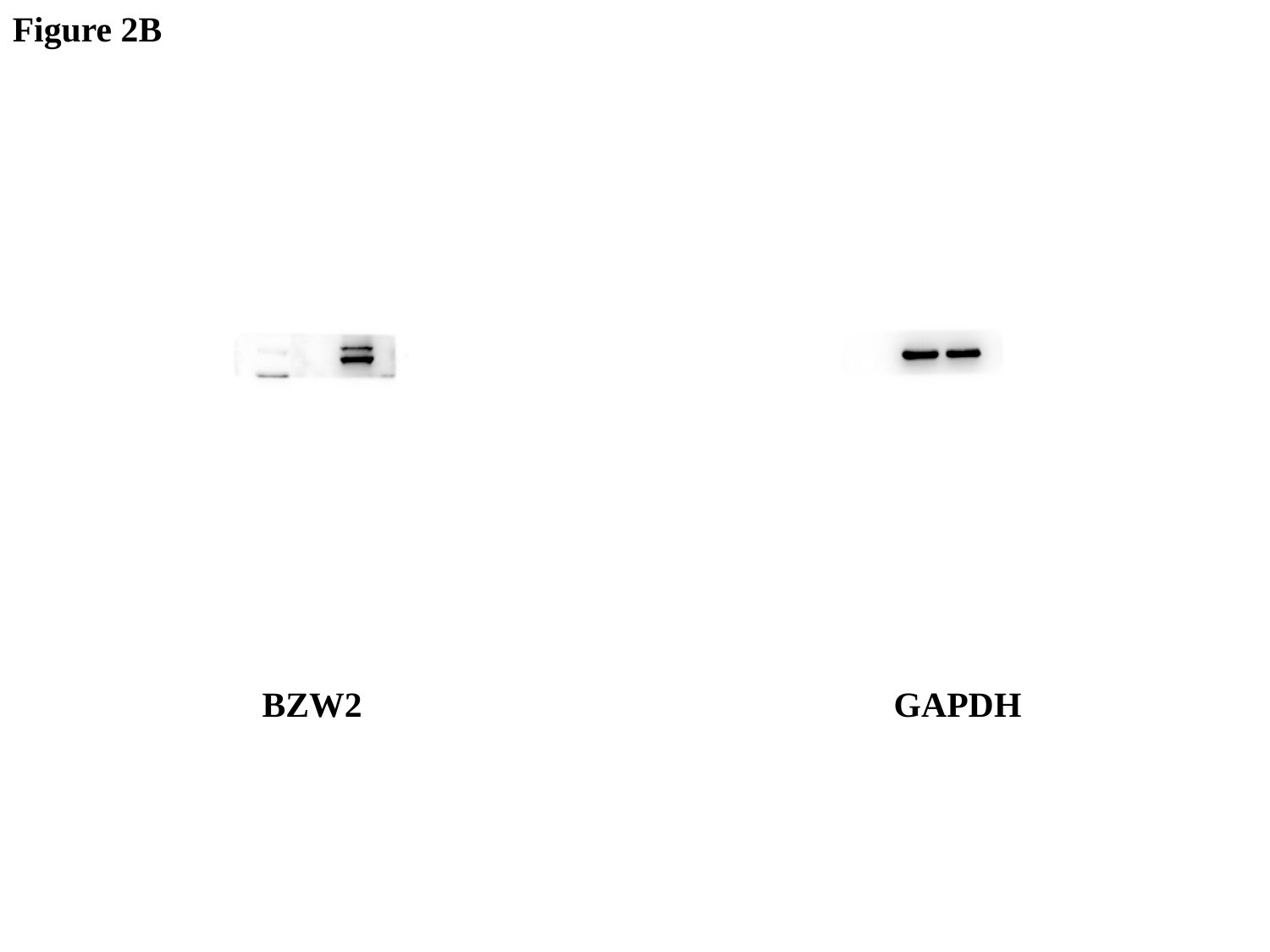

Figure 2B
GAPDH
BZW2

## Slide 15
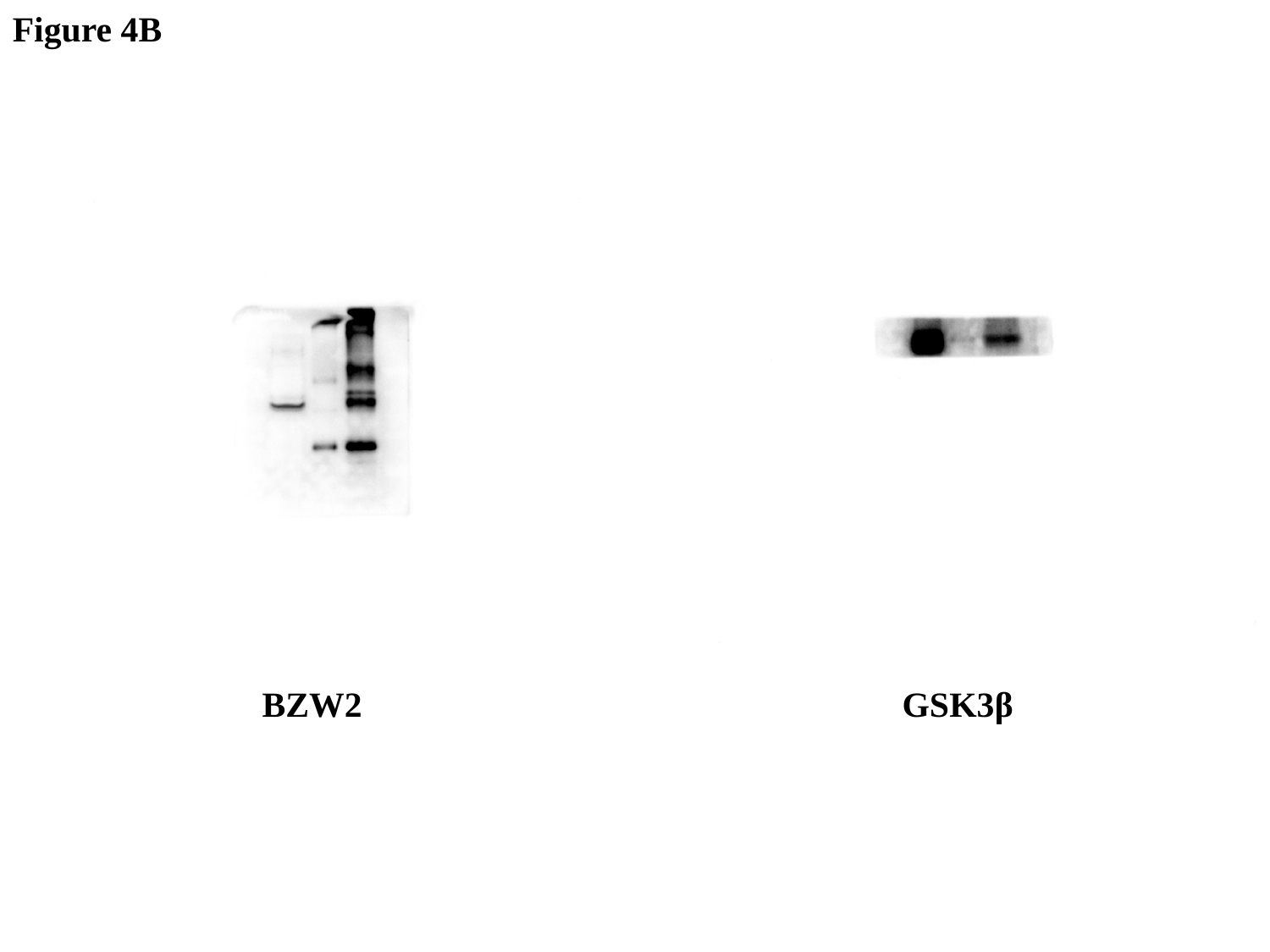

Figure 4B
GSK3β
BZW2

## Slide 16
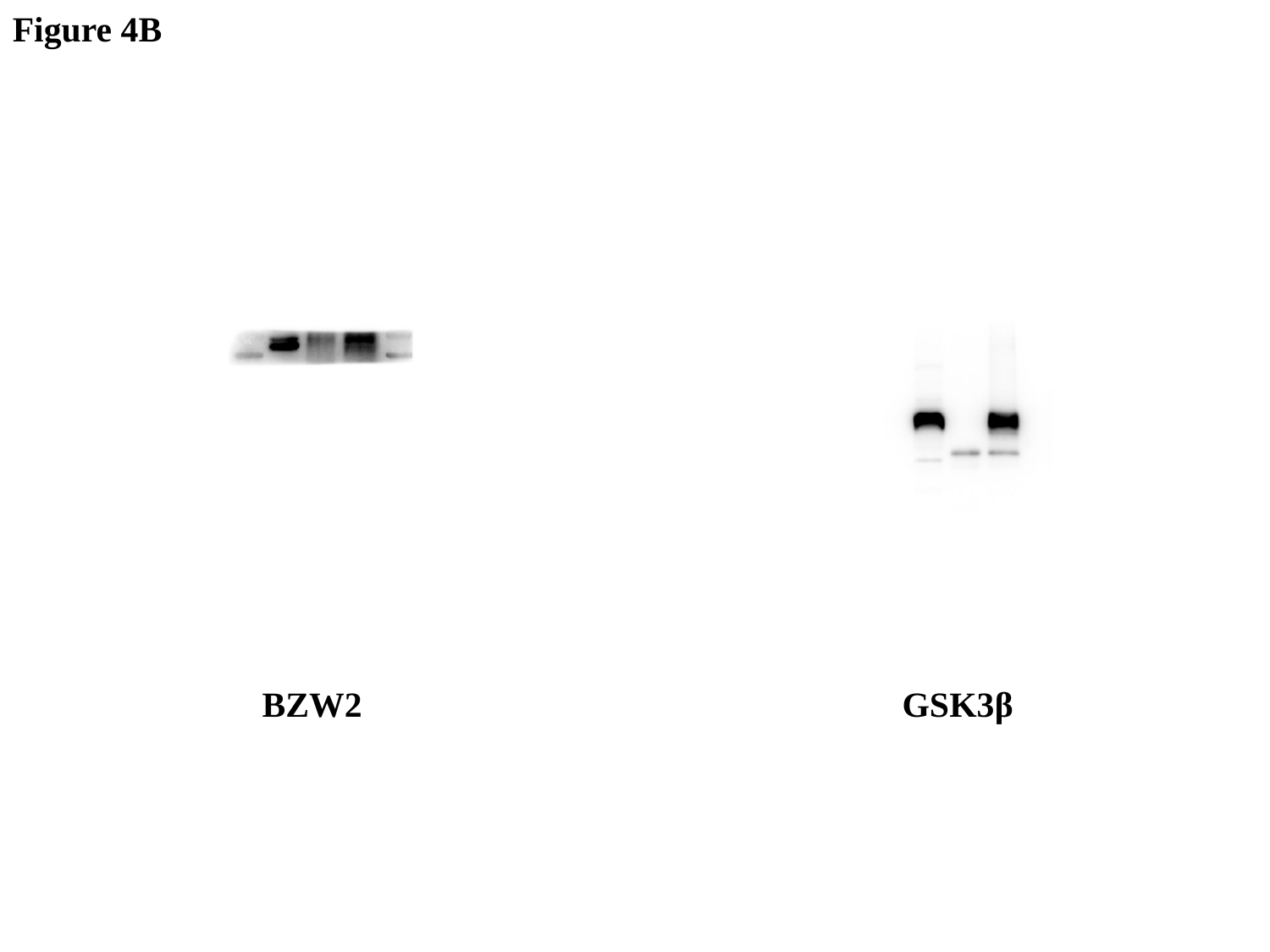

Figure 4B
GSK3β
BZW2

## Slide 17
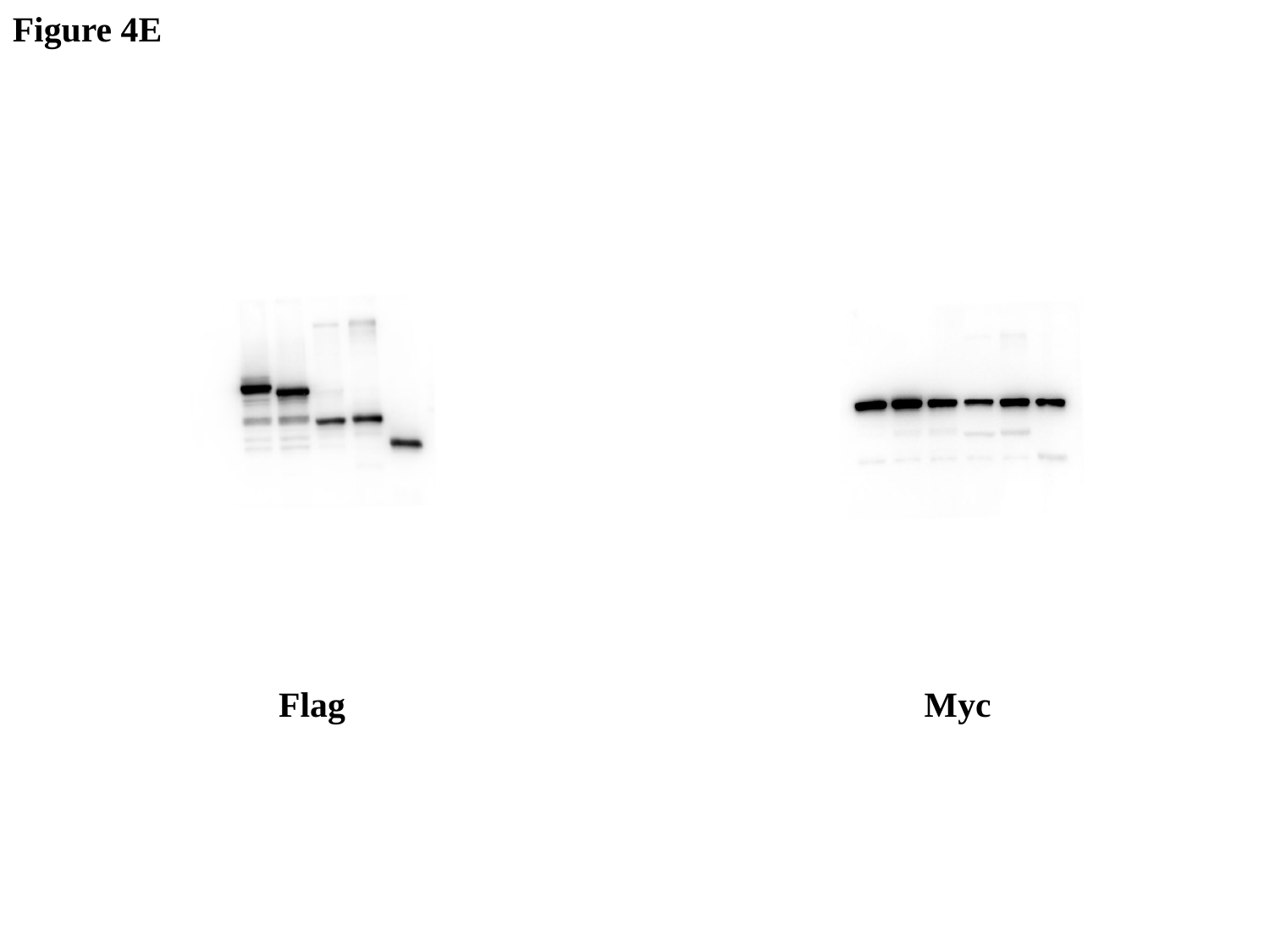

Figure 4E
Myc
Flag

## Slide 18
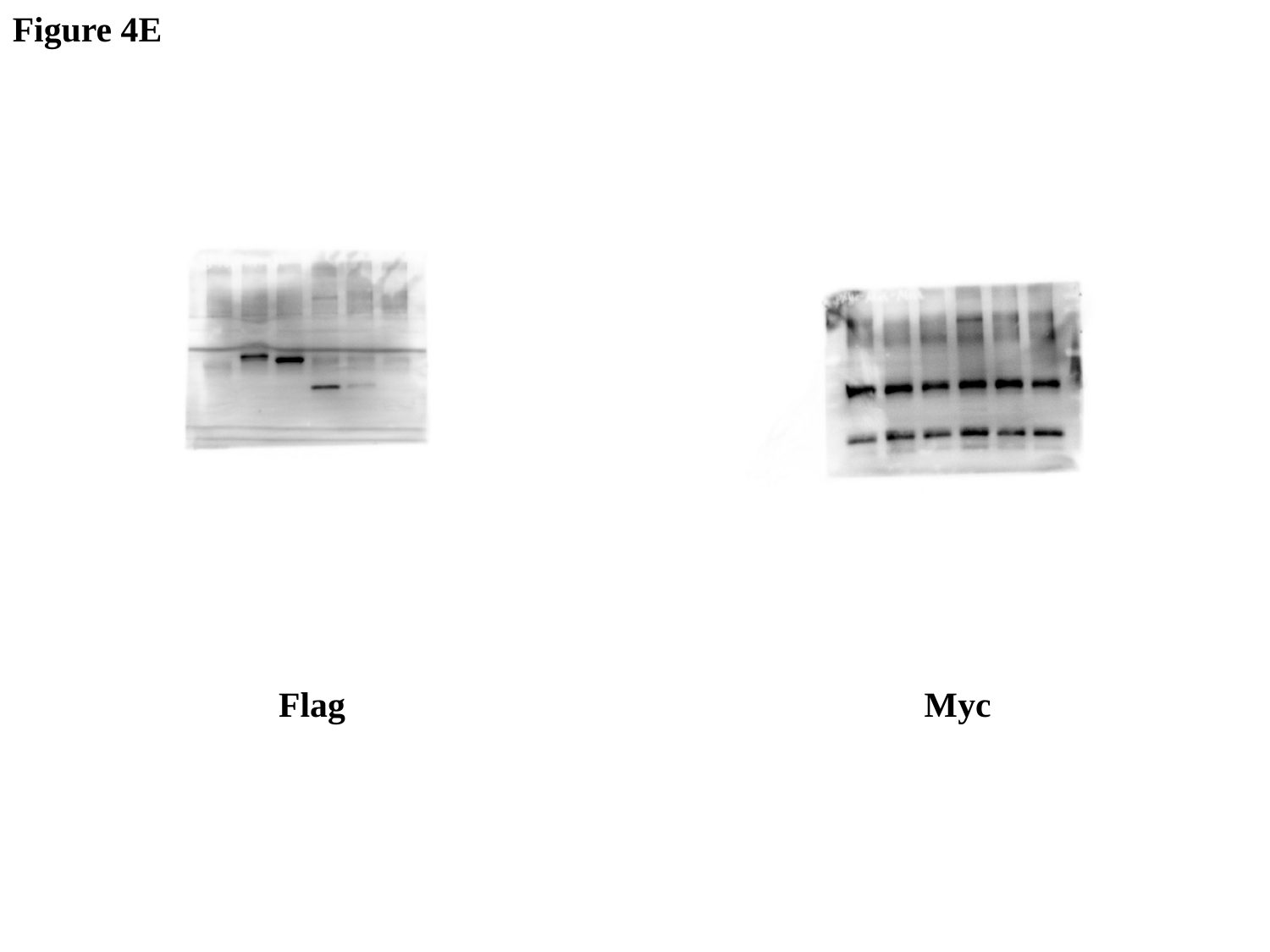

Figure 4E
Myc
Flag

## Slide 19
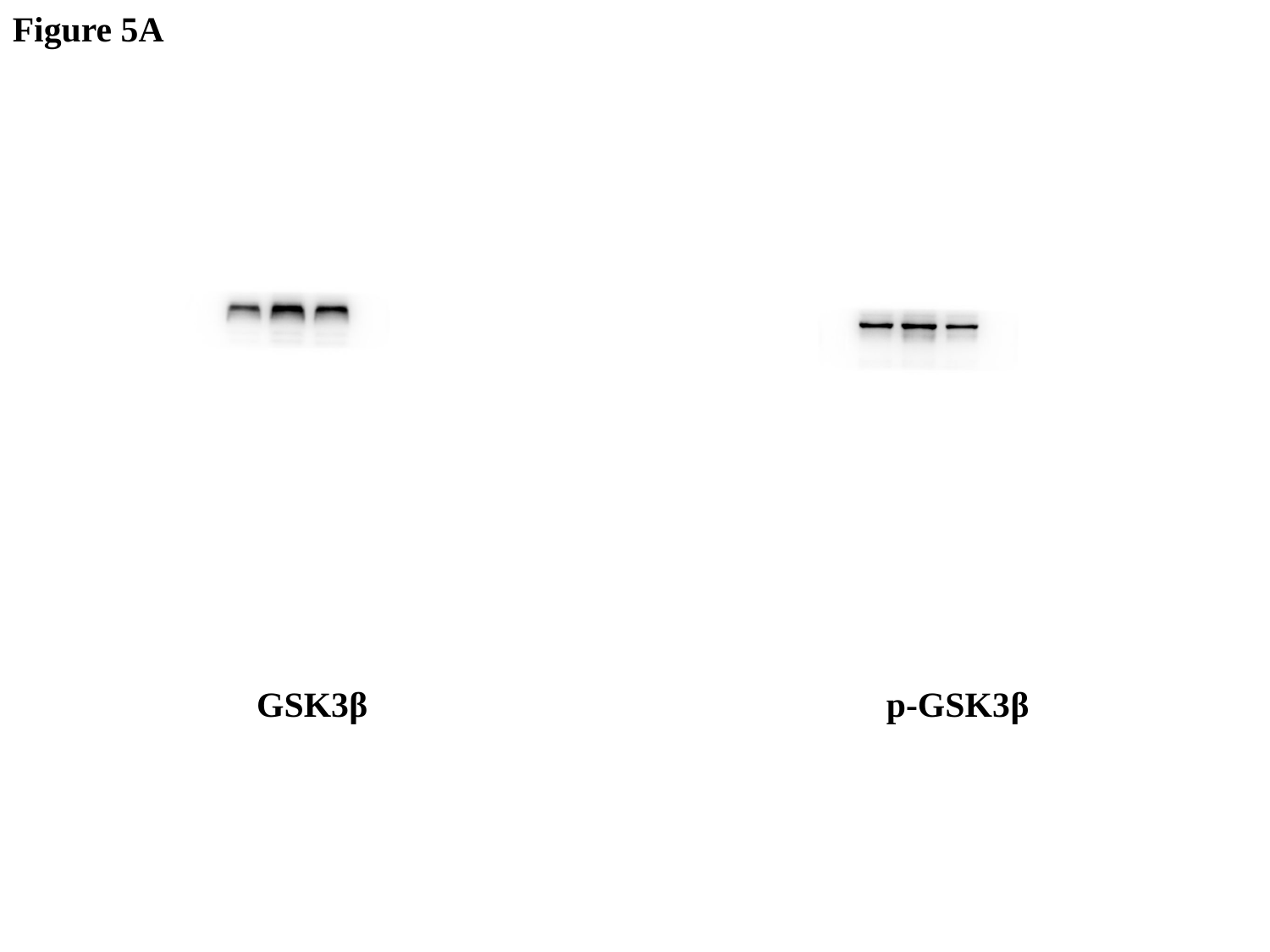

Figure 5A
p-GSK3β
GSK3β

## Slide 20
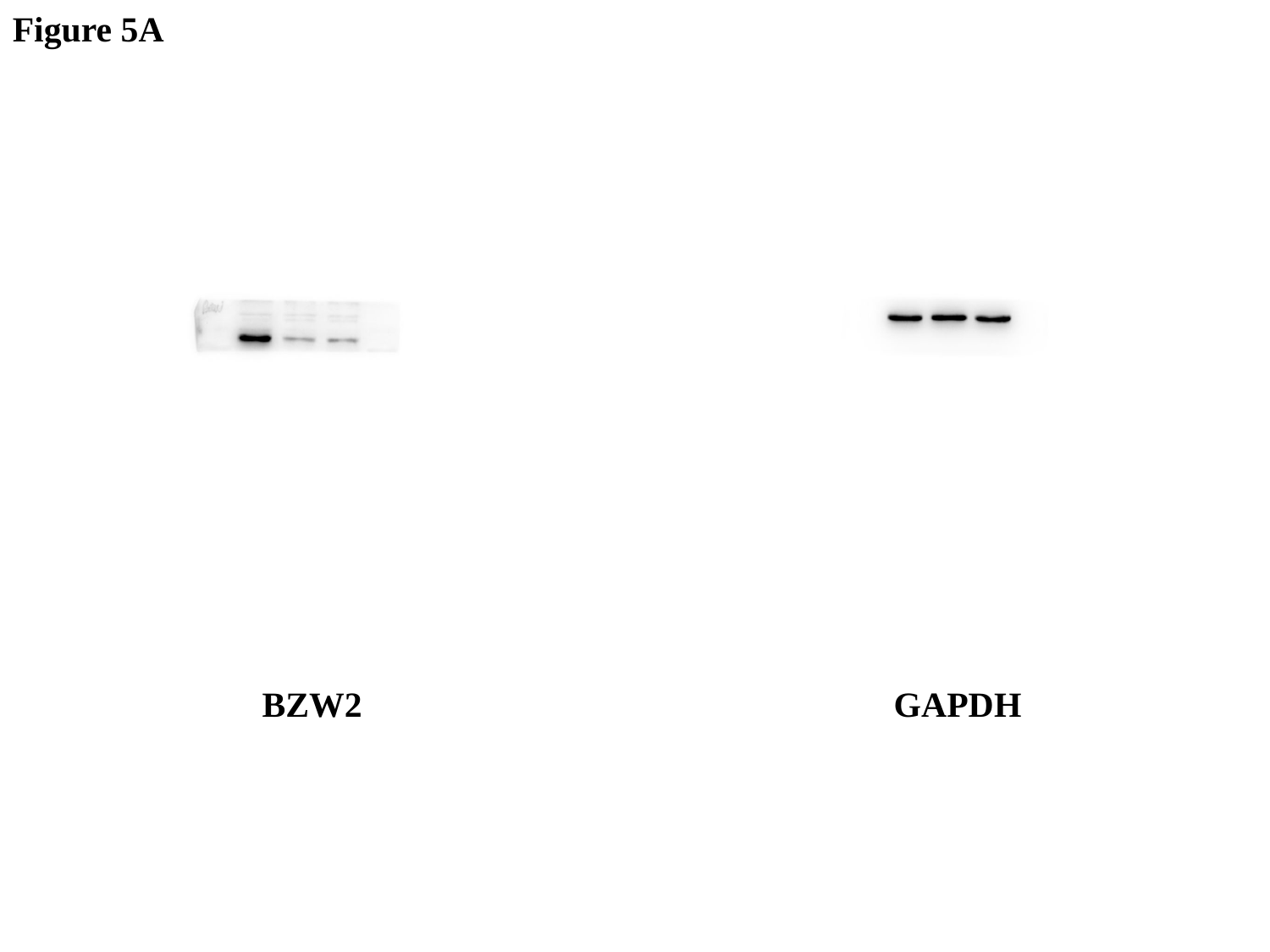

Figure 5A
GAPDH
BZW2

## Slide 21
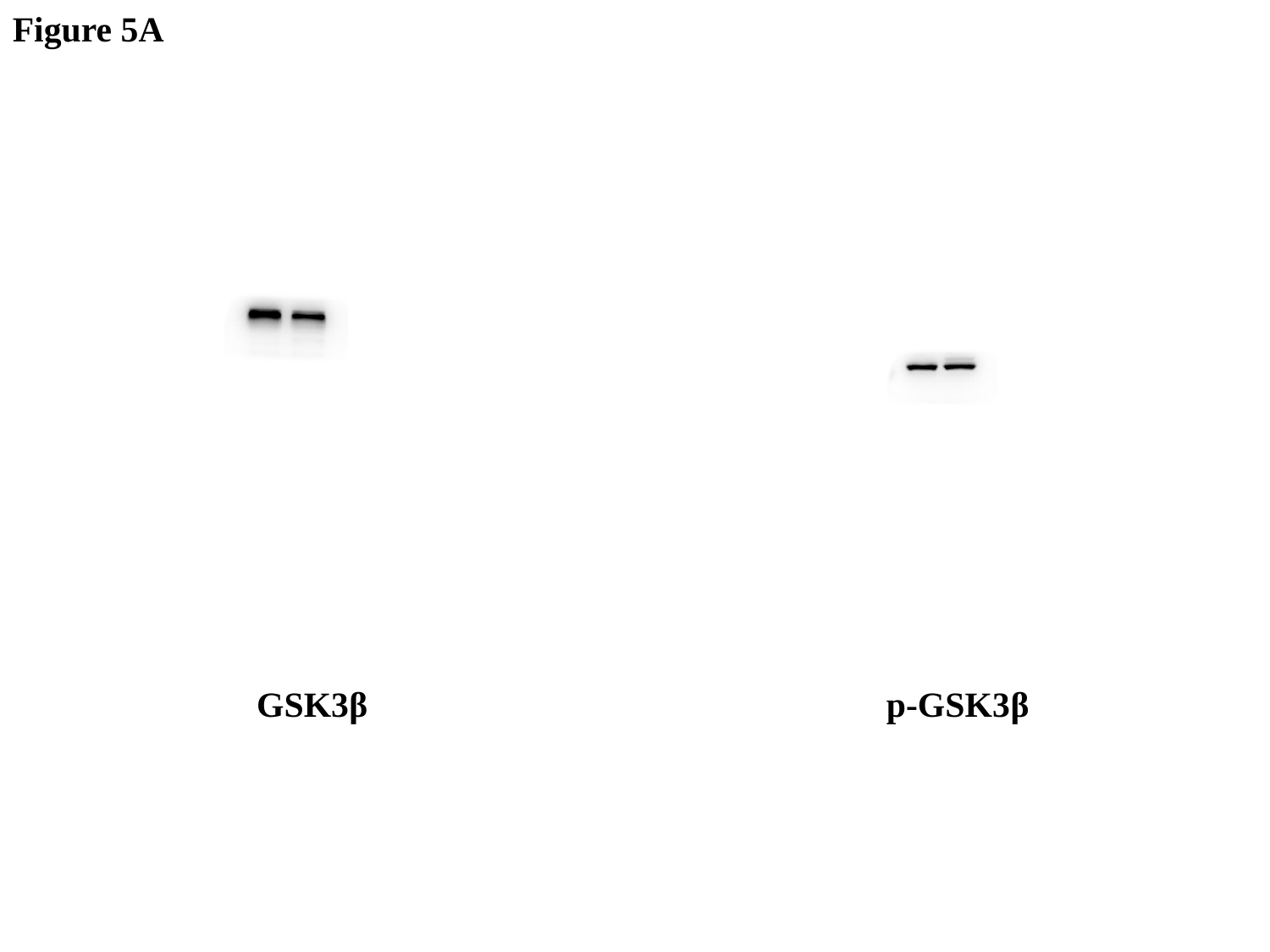

Figure 5A
p-GSK3β
GSK3β

## Slide 22
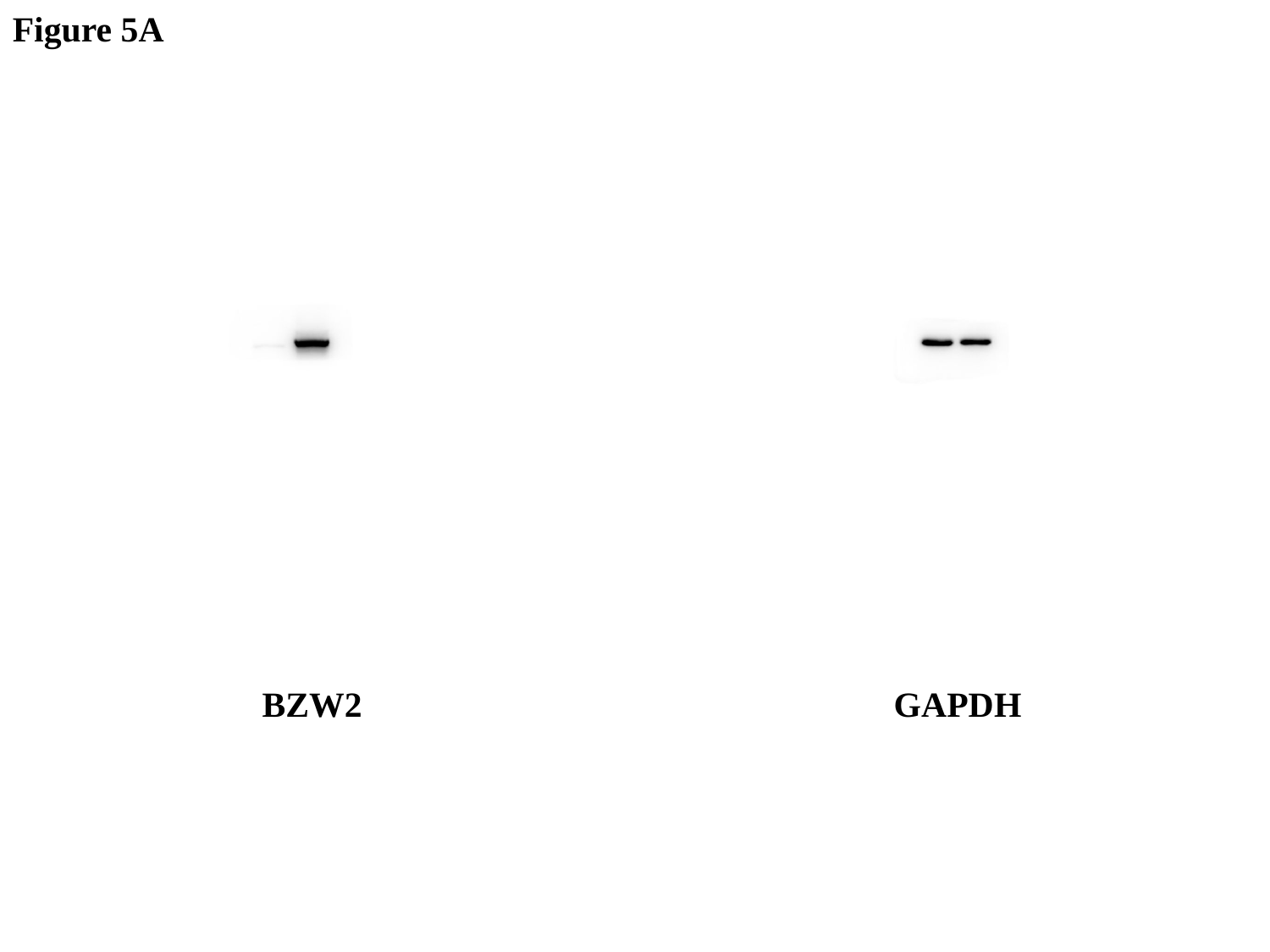

Figure 5A
GAPDH
BZW2

## Slide 23
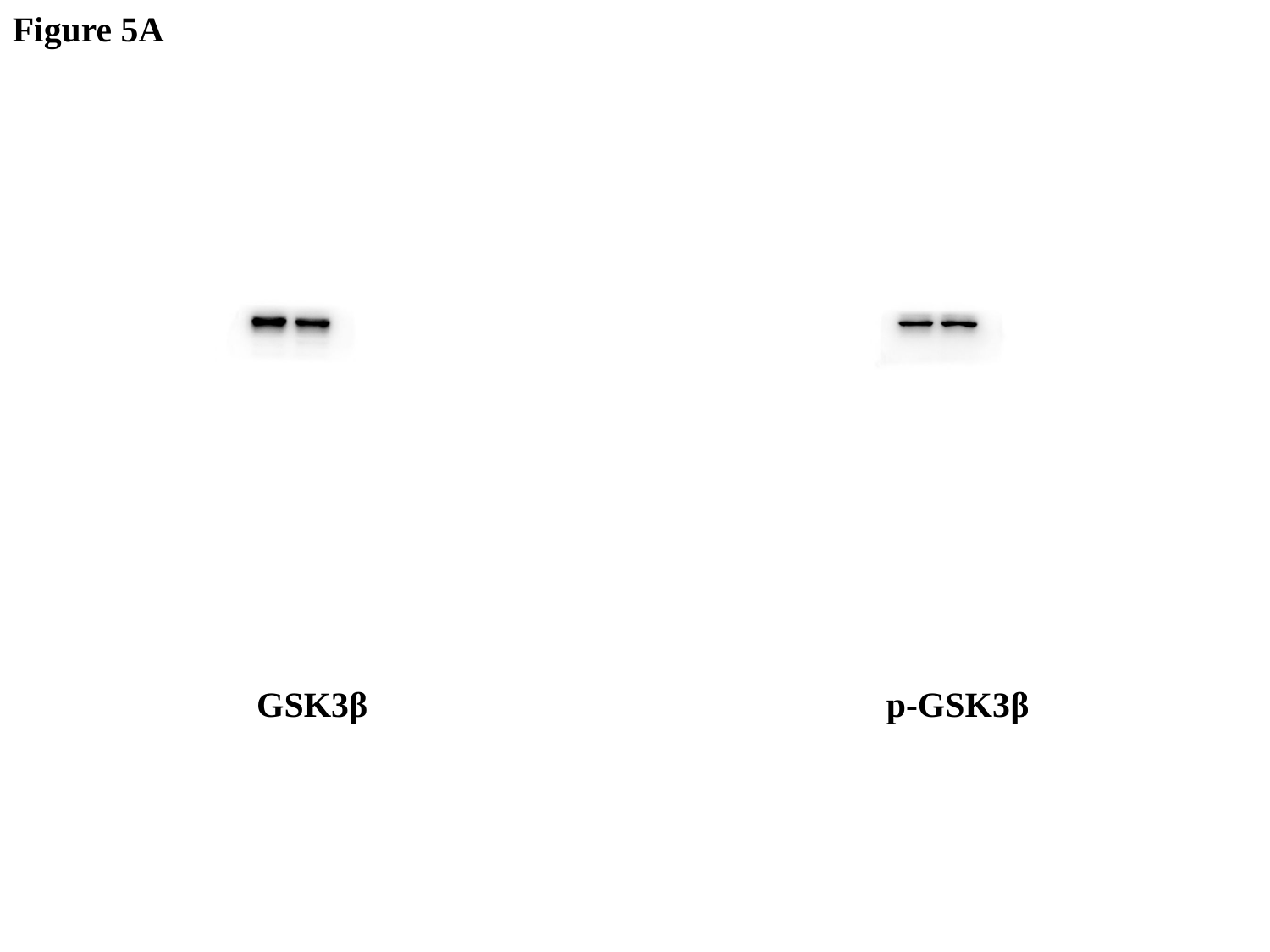

Figure 5A
p-GSK3β
GSK3β

## Slide 24
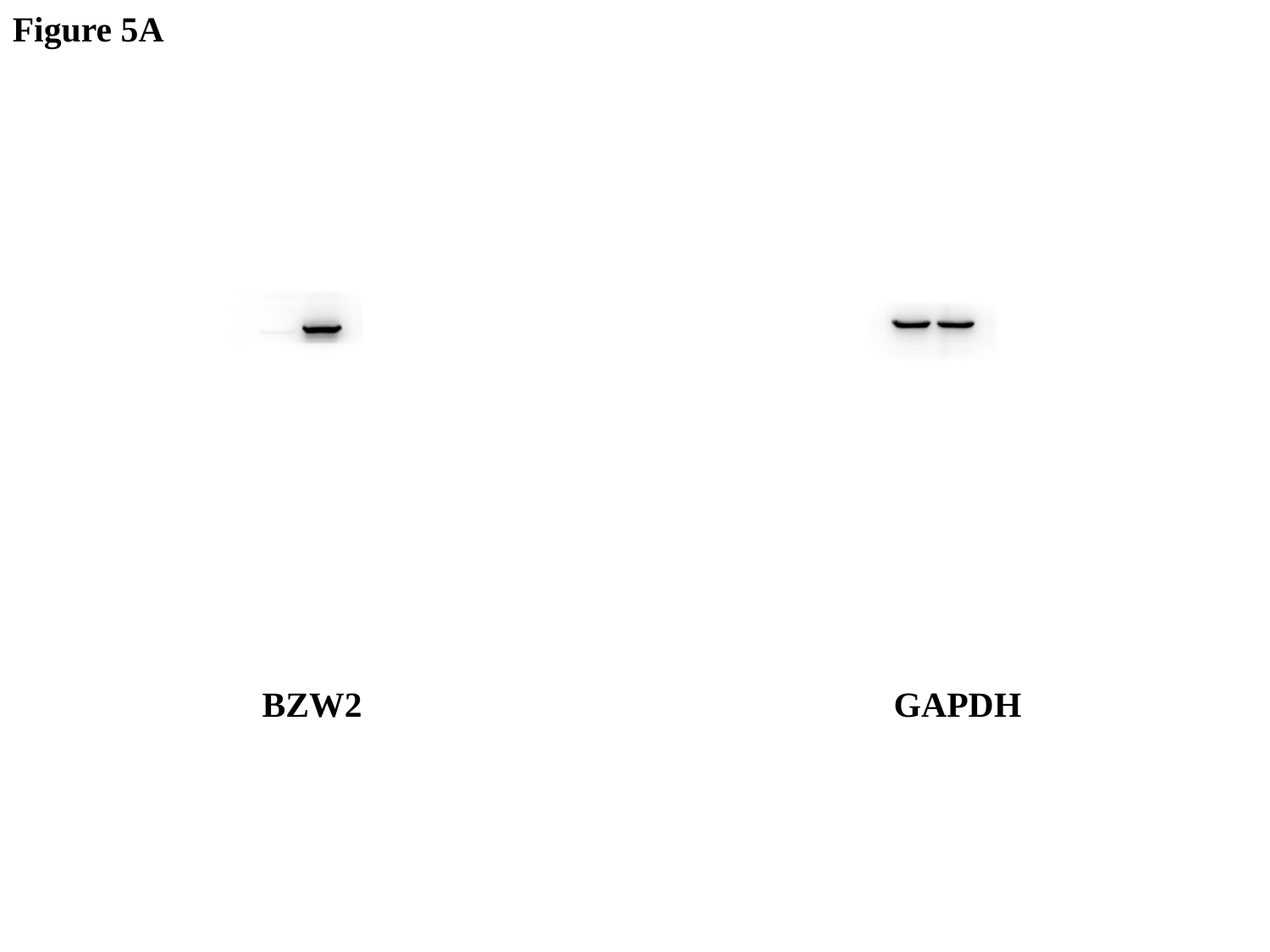

Figure 5A
GAPDH
BZW2

## Slide 25
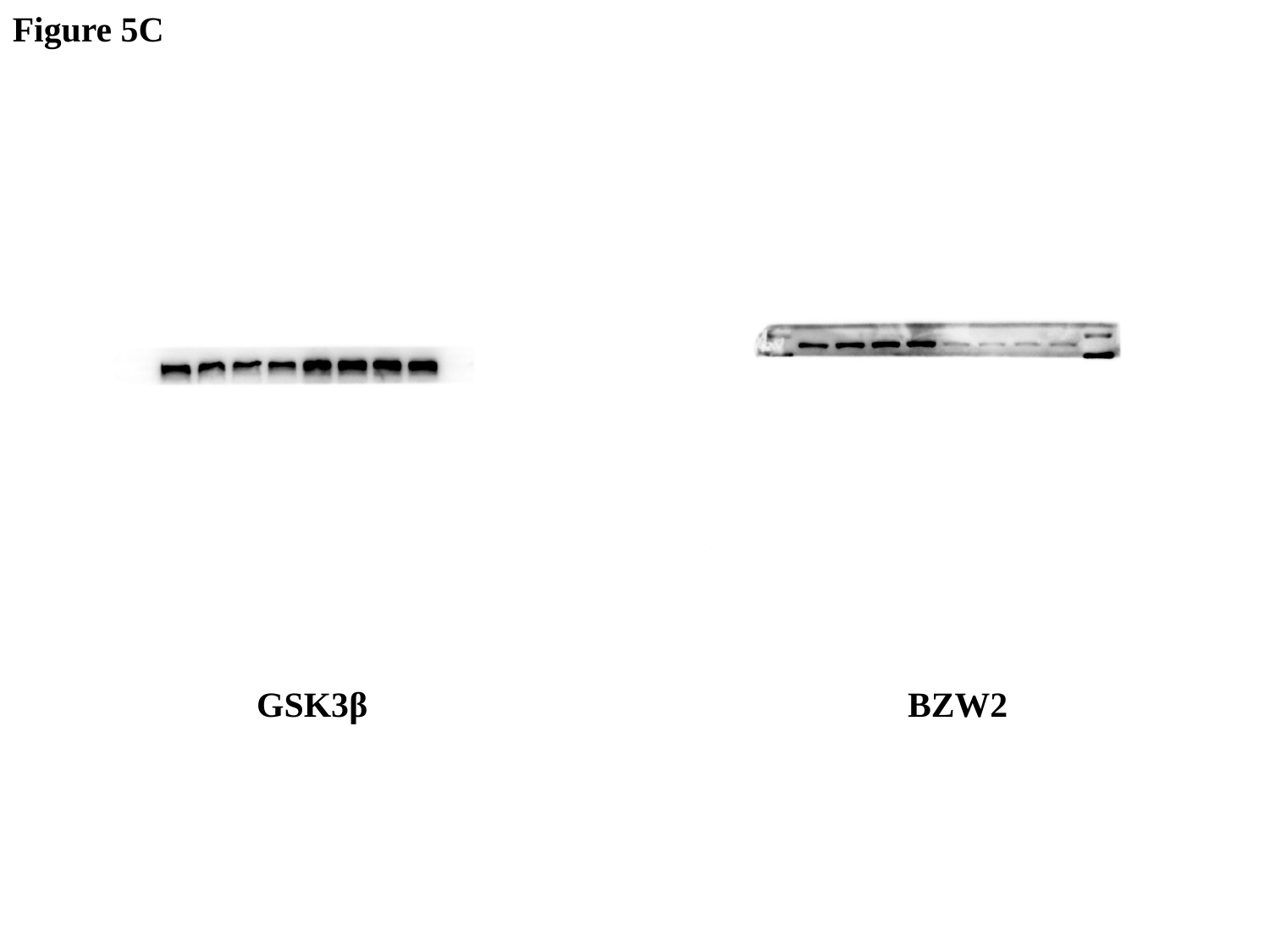

Figure 5C
BZW2
GSK3β

## Slide 26
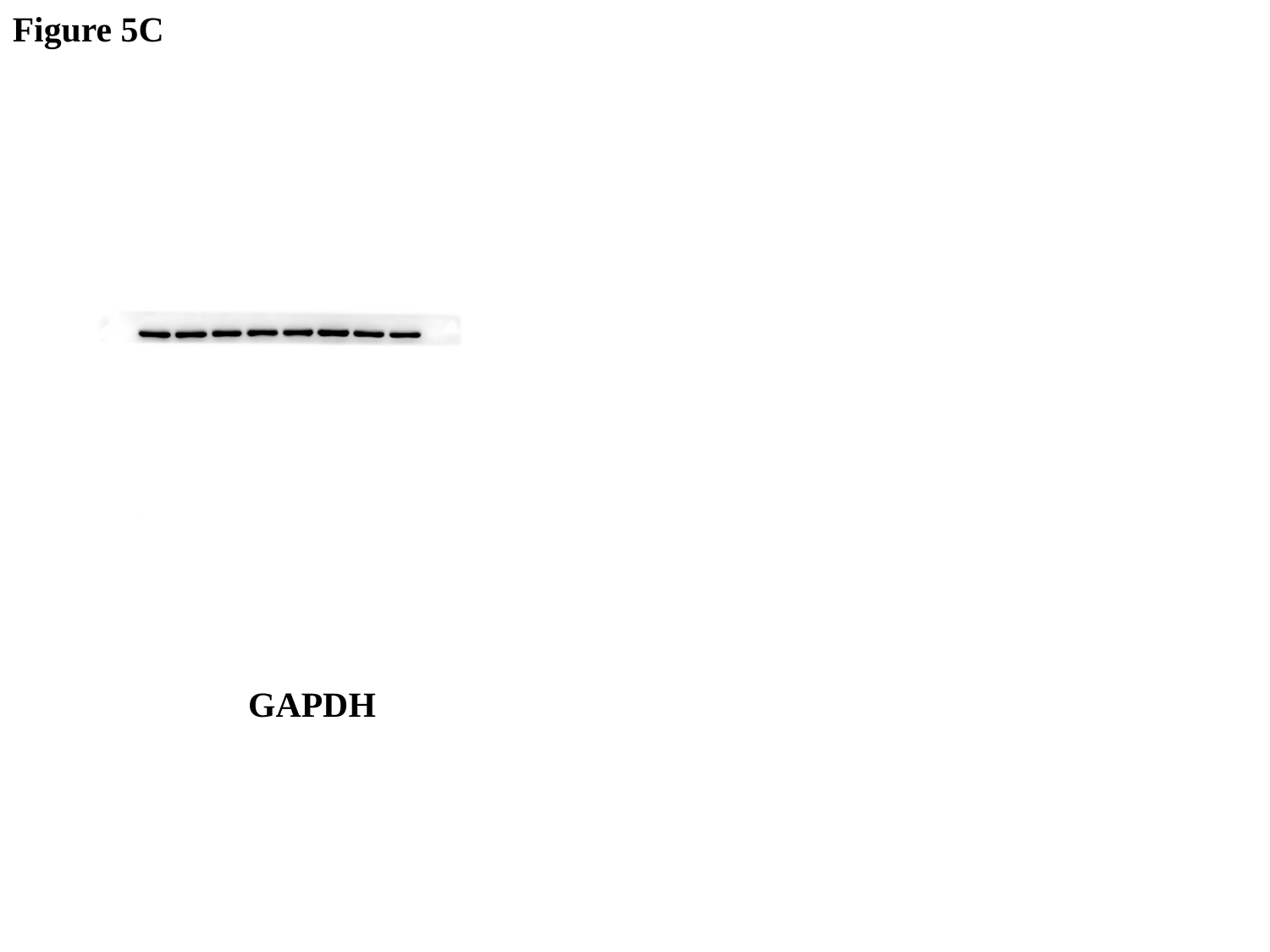

Figure 5C
GAPDH

## Slide 27
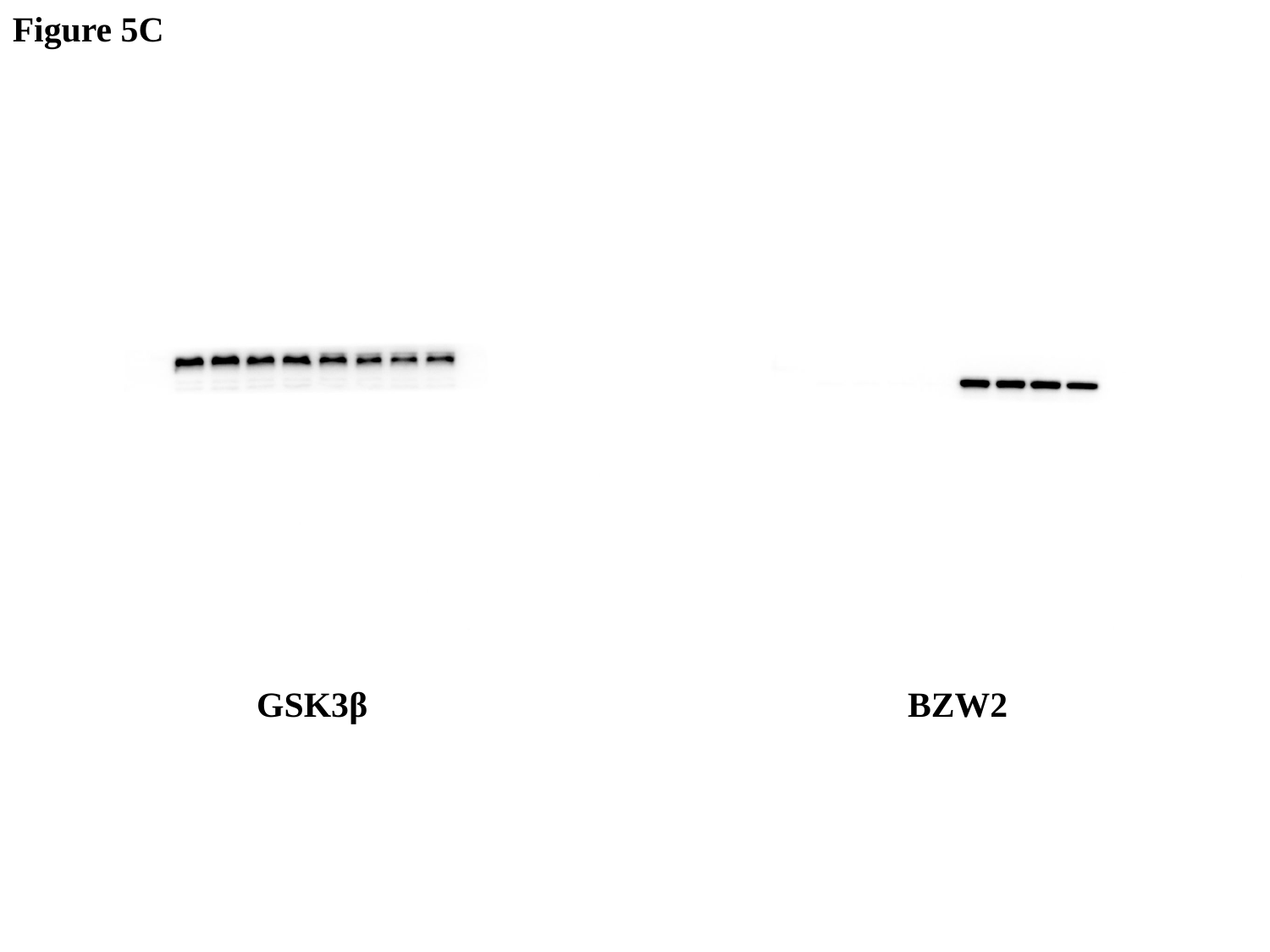

Figure 5C
BZW2
GSK3β

## Slide 28
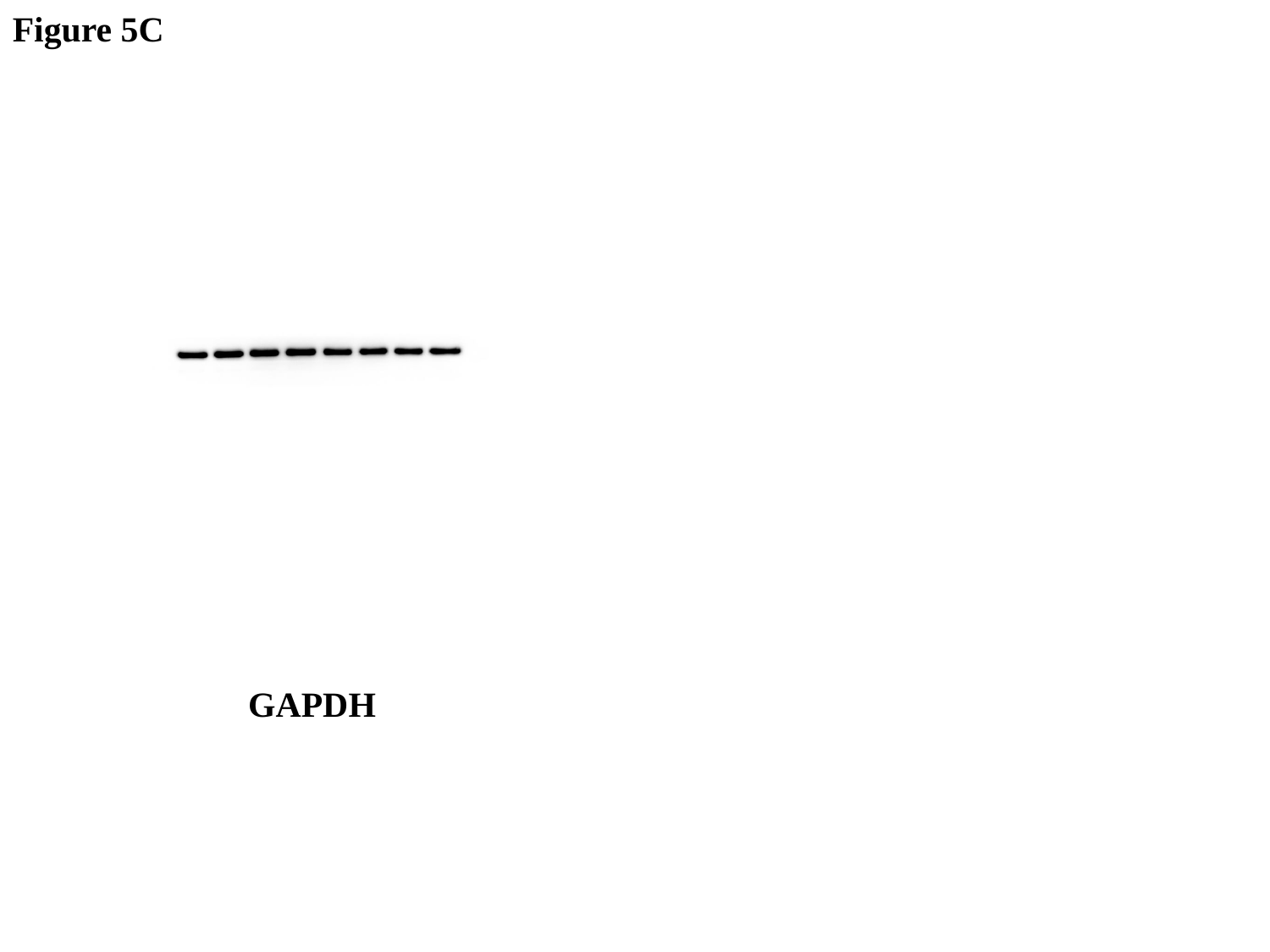

Figure 5C
GAPDH

## Slide 29
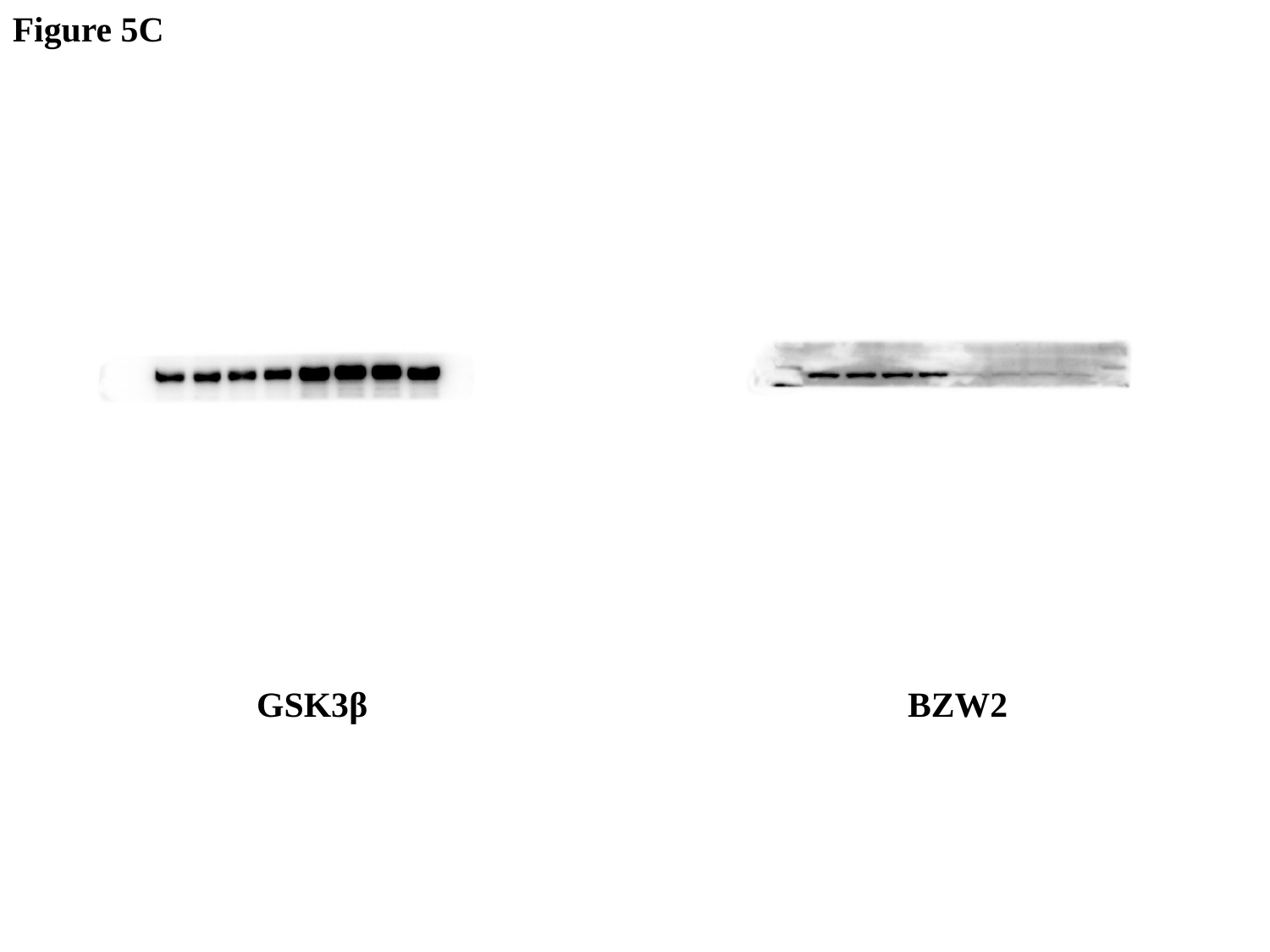

Figure 5C
BZW2
GSK3β

## Slide 30
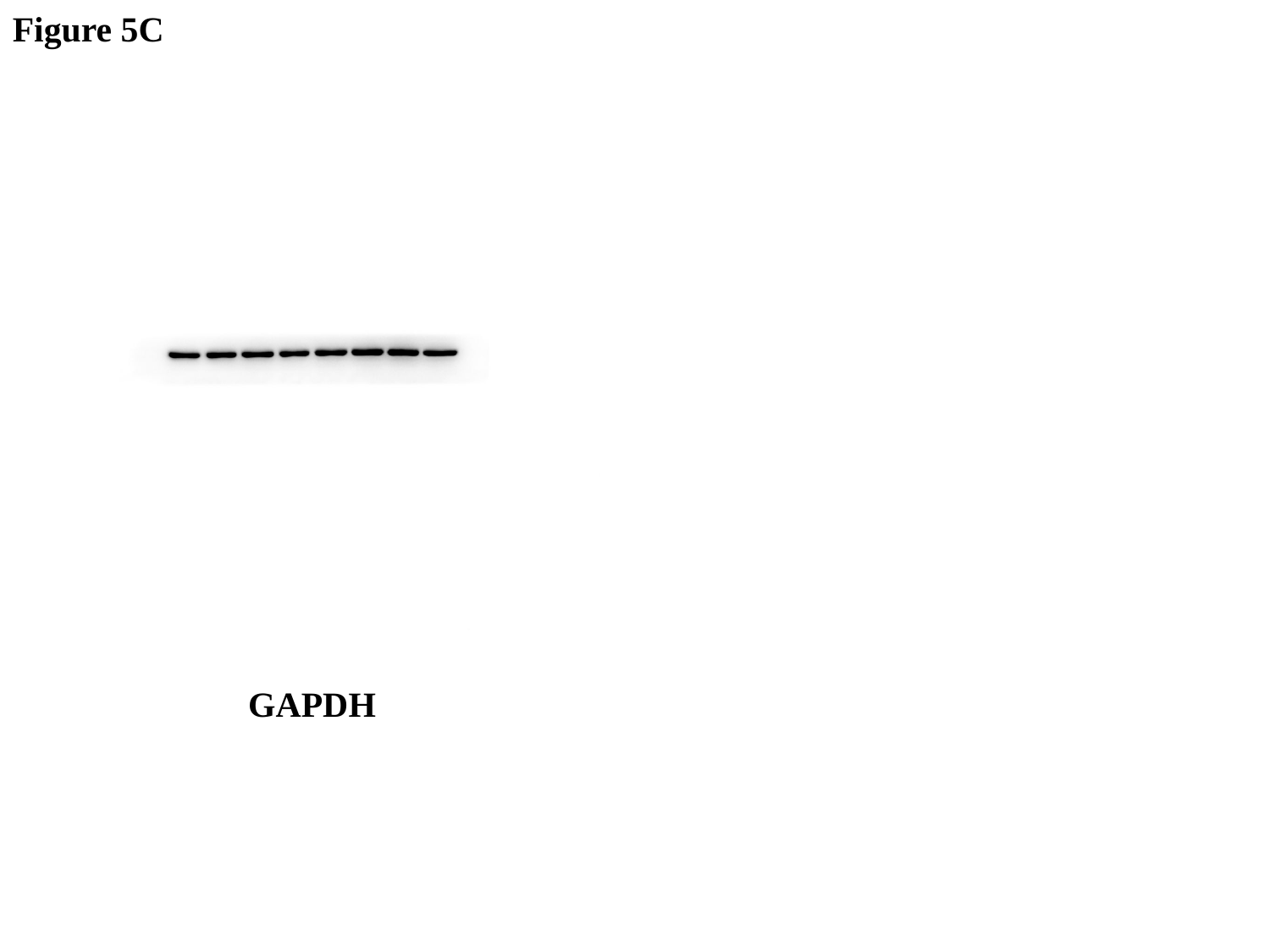

Figure 5C
GAPDH

## Slide 31
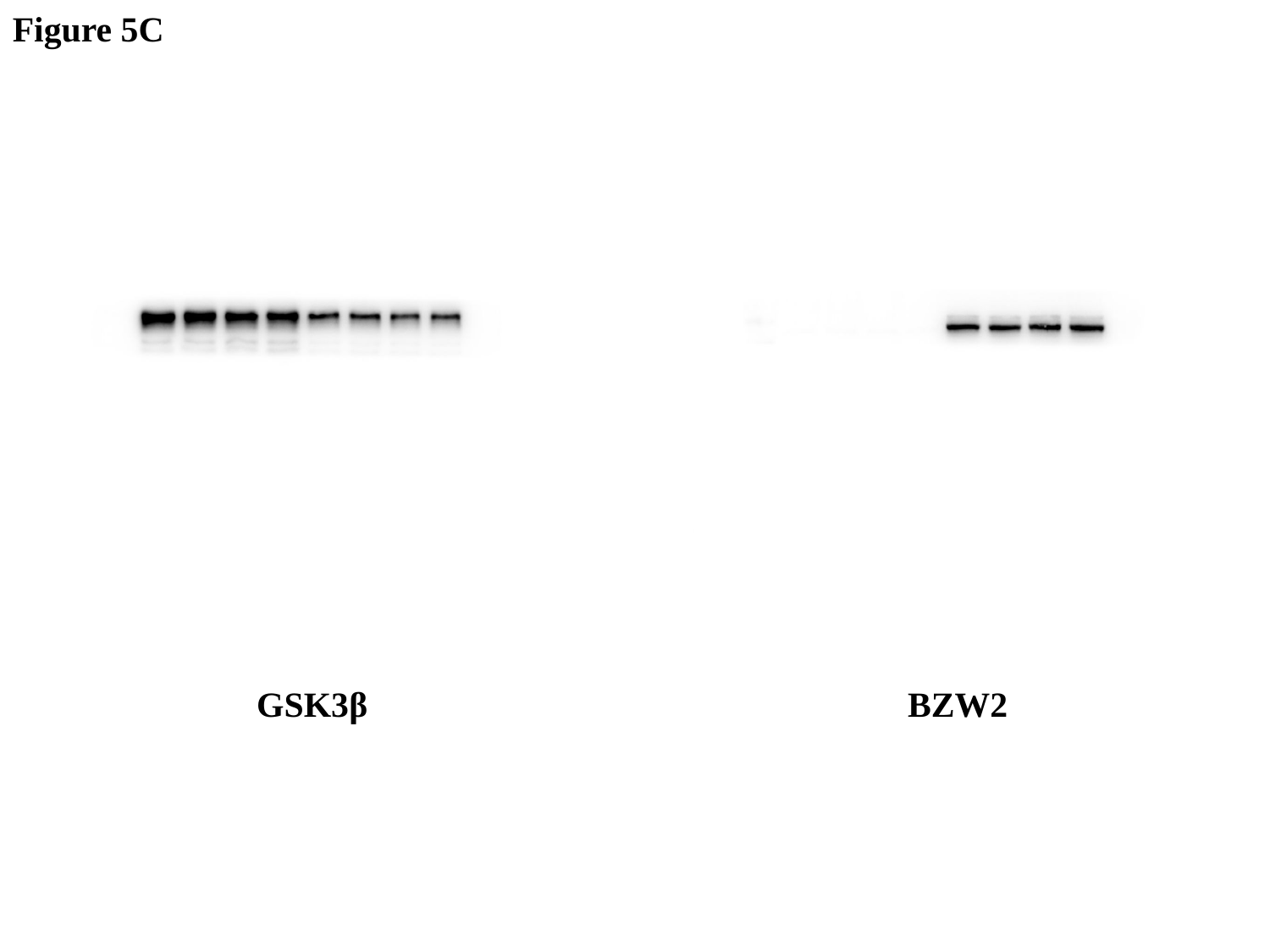

Figure 5C
BZW2
GSK3β

## Slide 32
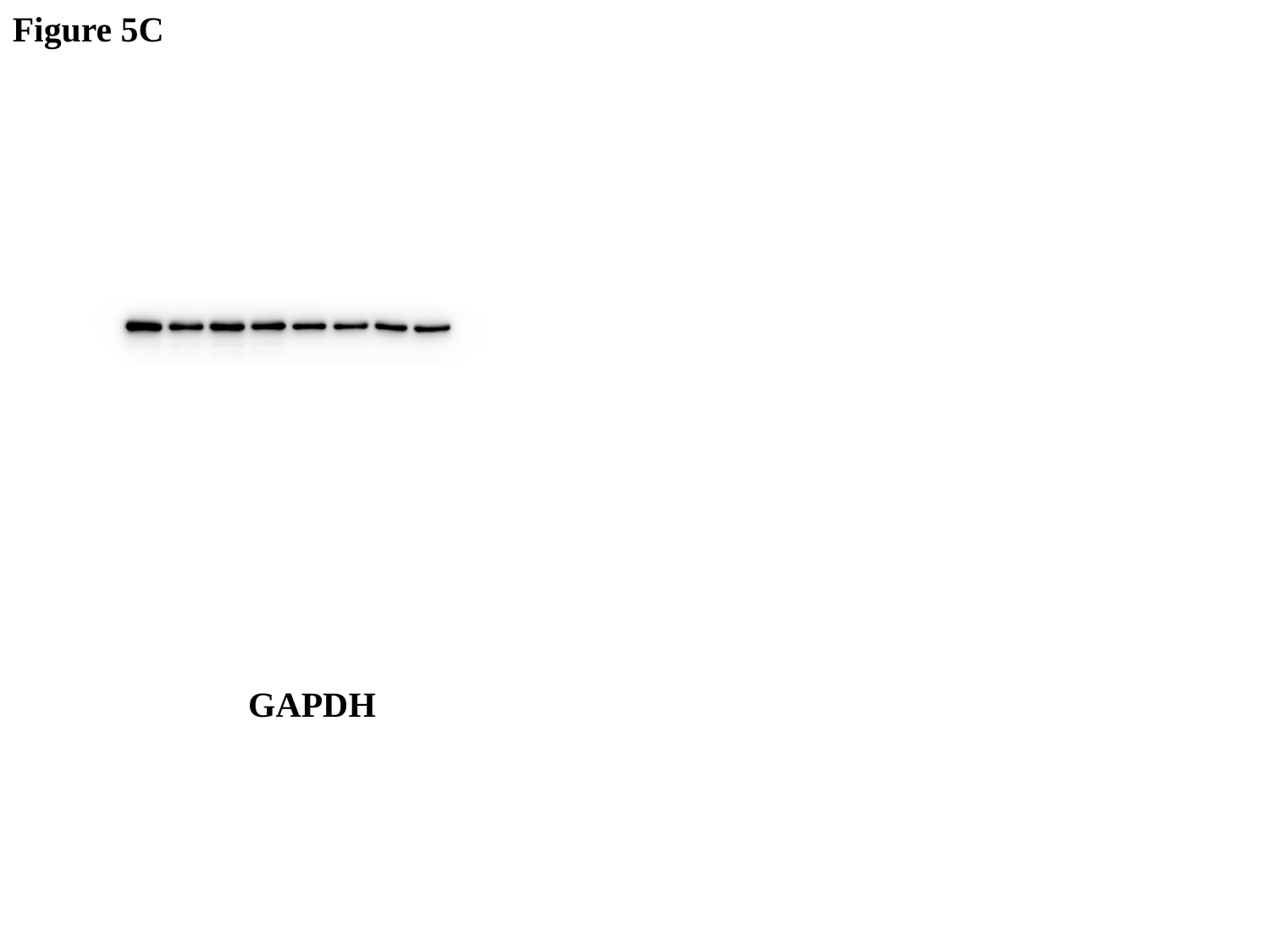

Figure 5C
GAPDH

## Slide 33
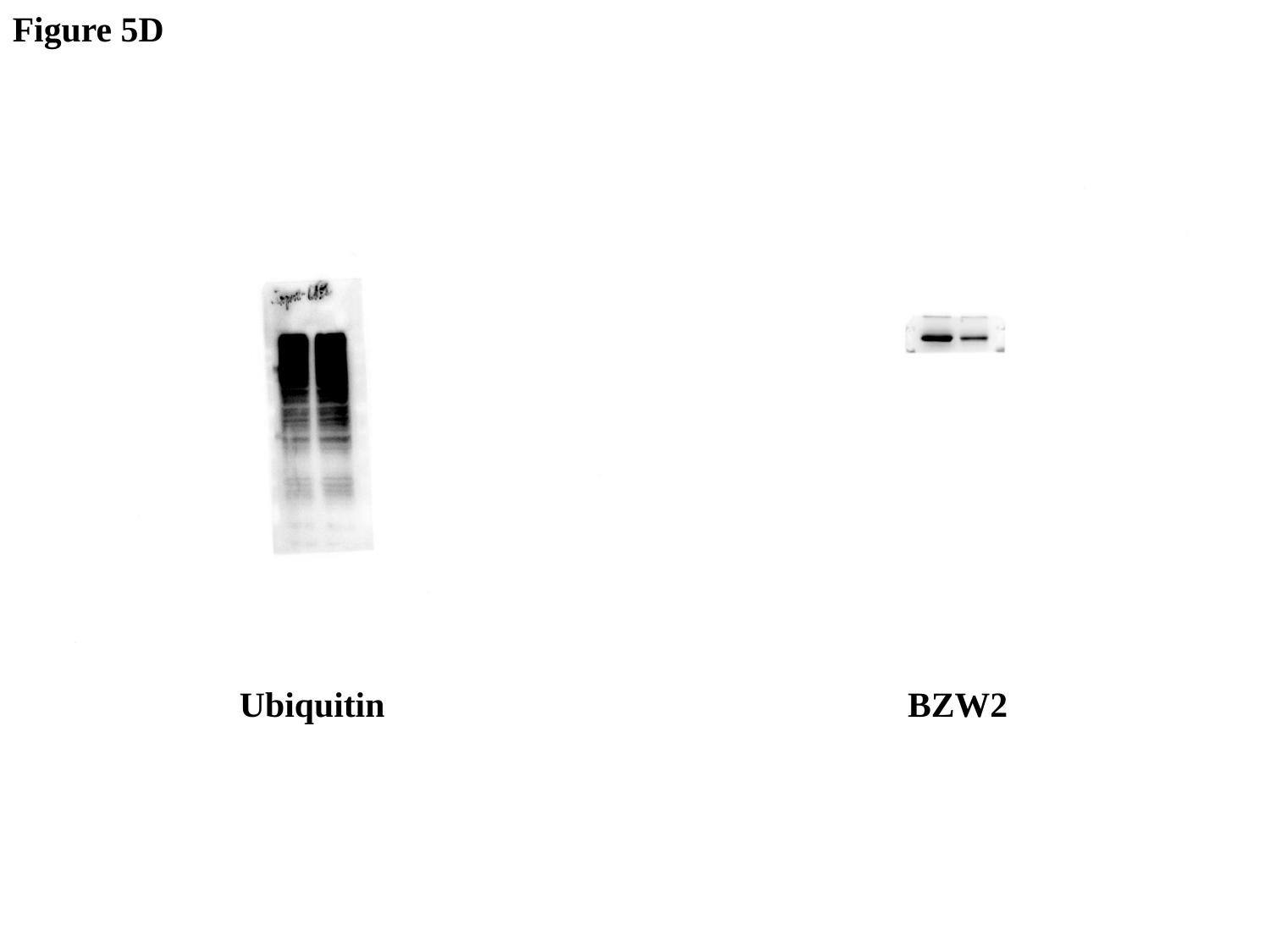

Figure 5D
BZW2
Ubiquitin

## Slide 34
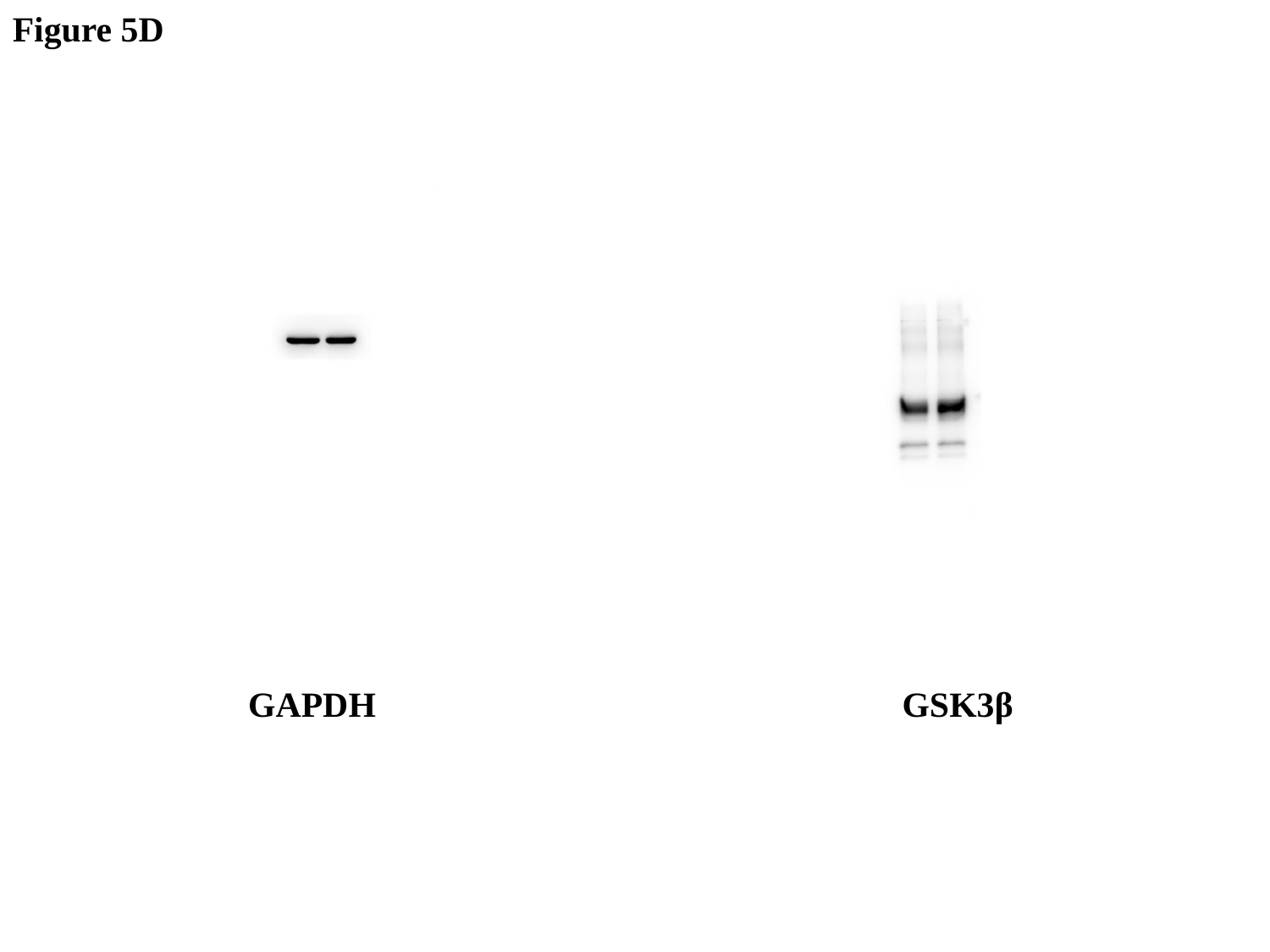

Figure 5D
GSK3β
GAPDH

## Slide 35
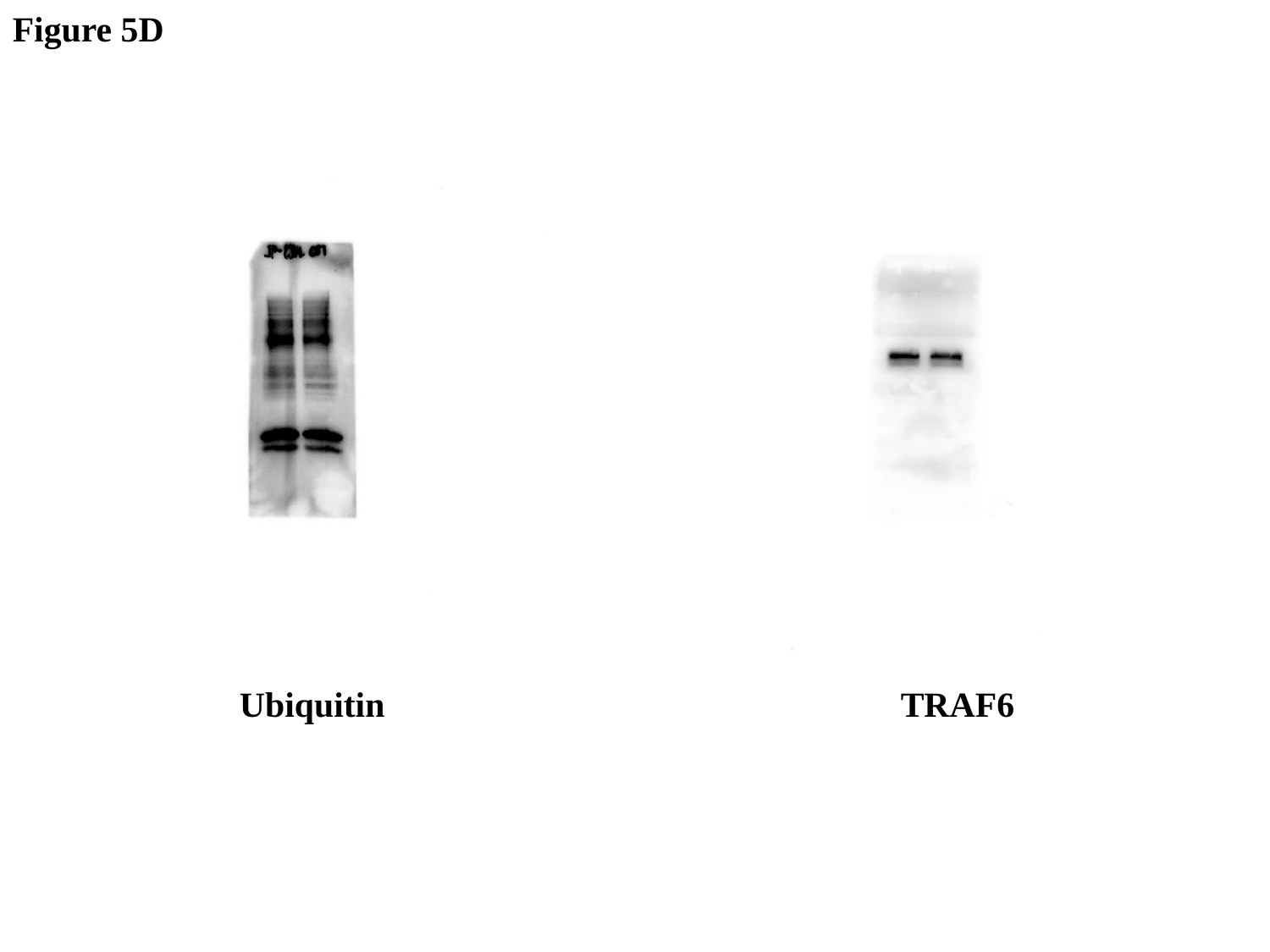

Figure 5D
TRAF6
Ubiquitin

## Slide 36
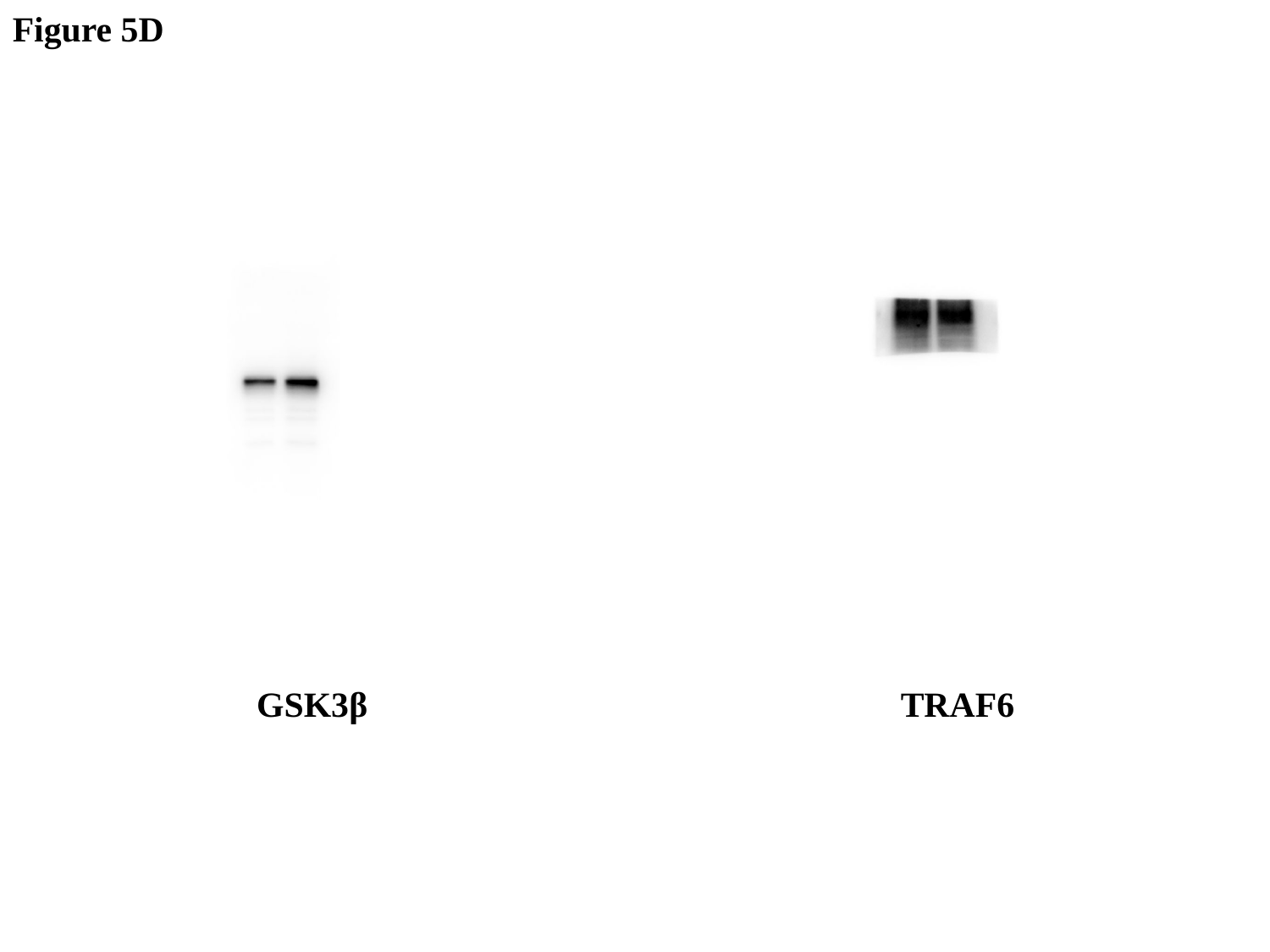

Figure 5D
TRAF6
GSK3β

## Slide 37
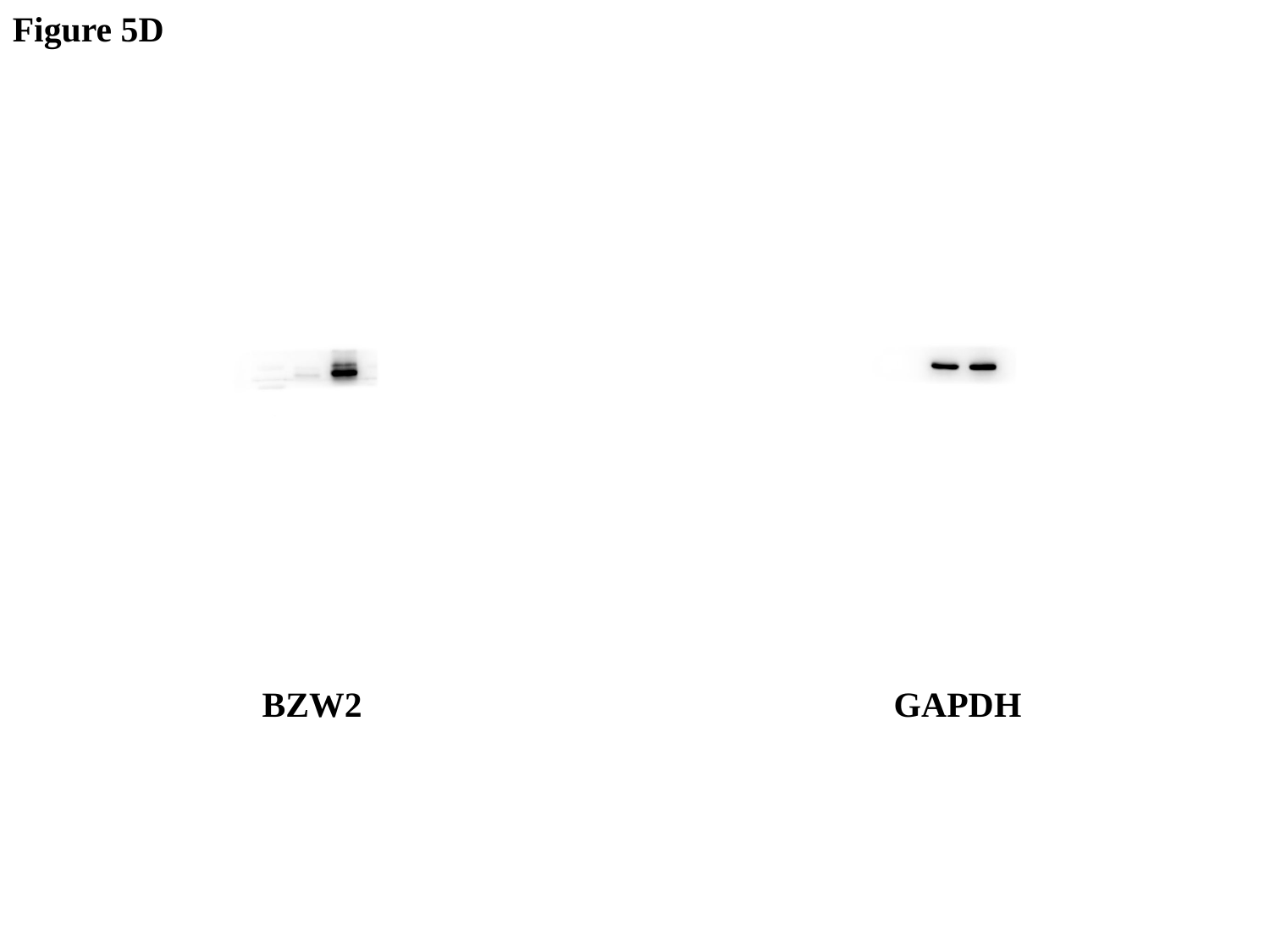

Figure 5D
GAPDH
BZW2

## Slide 38
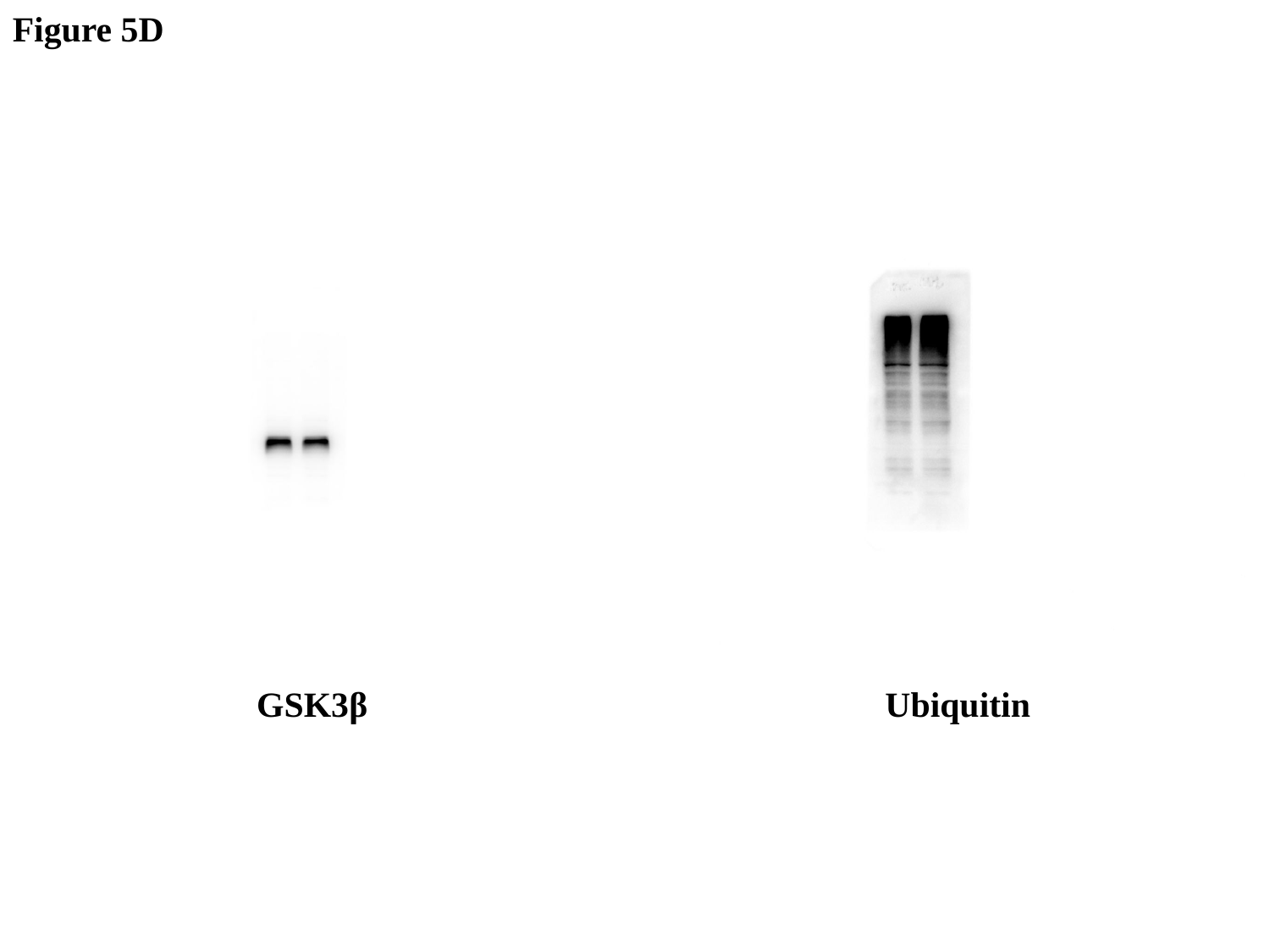

Figure 5D
Ubiquitin
GSK3β

## Slide 39
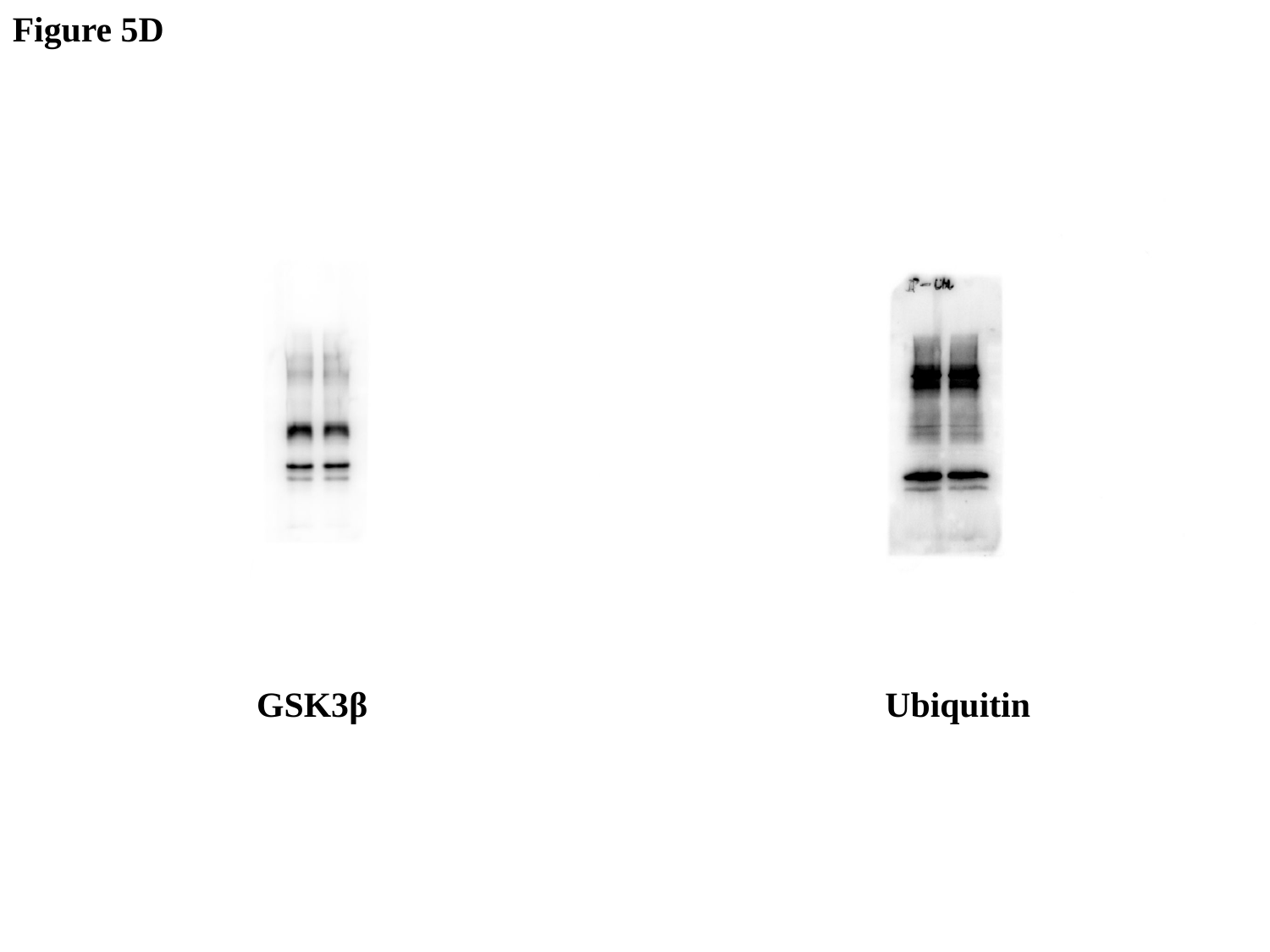

Figure 5D
Ubiquitin
GSK3β

## Slide 40
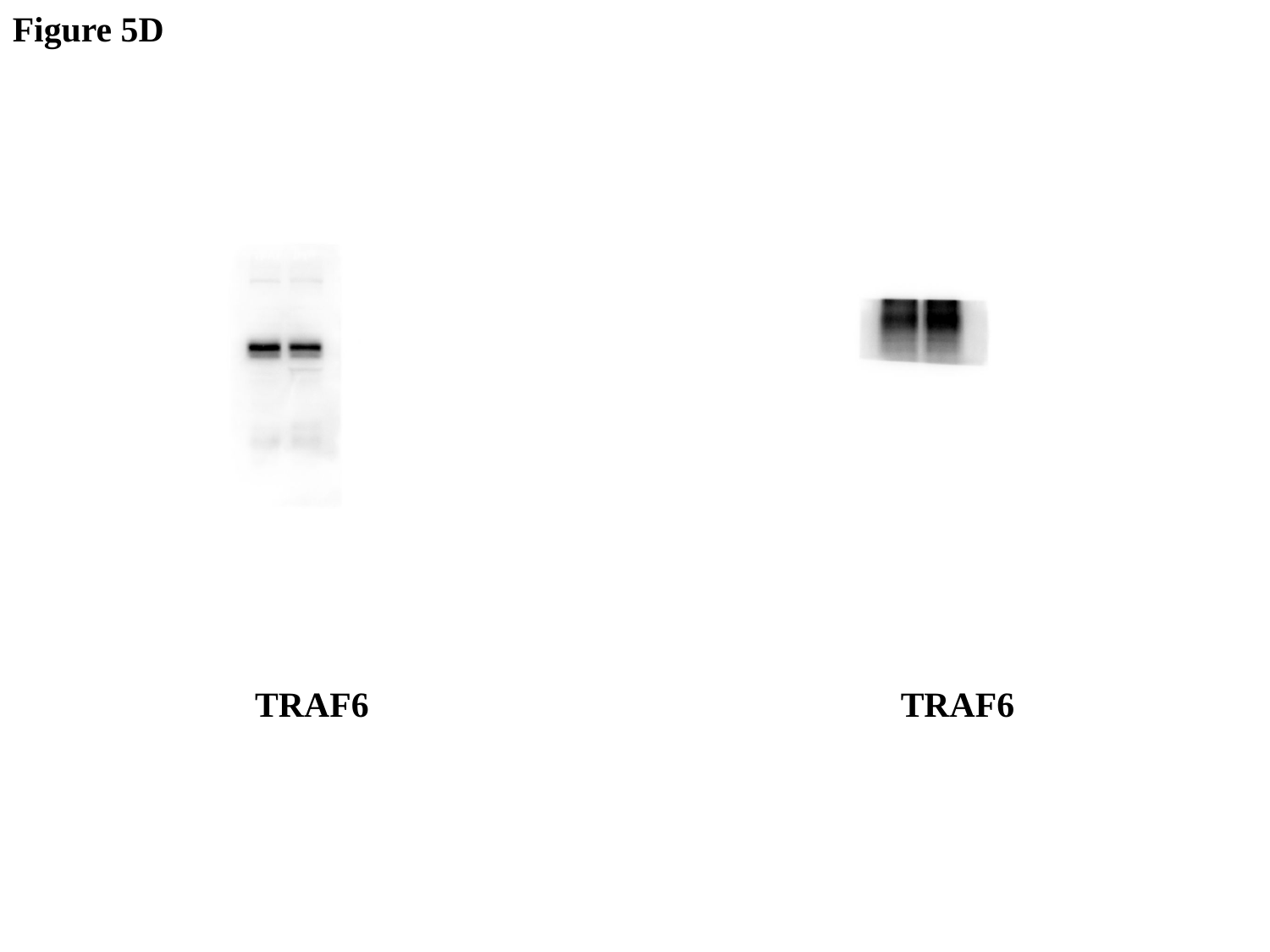

Figure 5D
TRAF6
TRAF6

## Slide 41
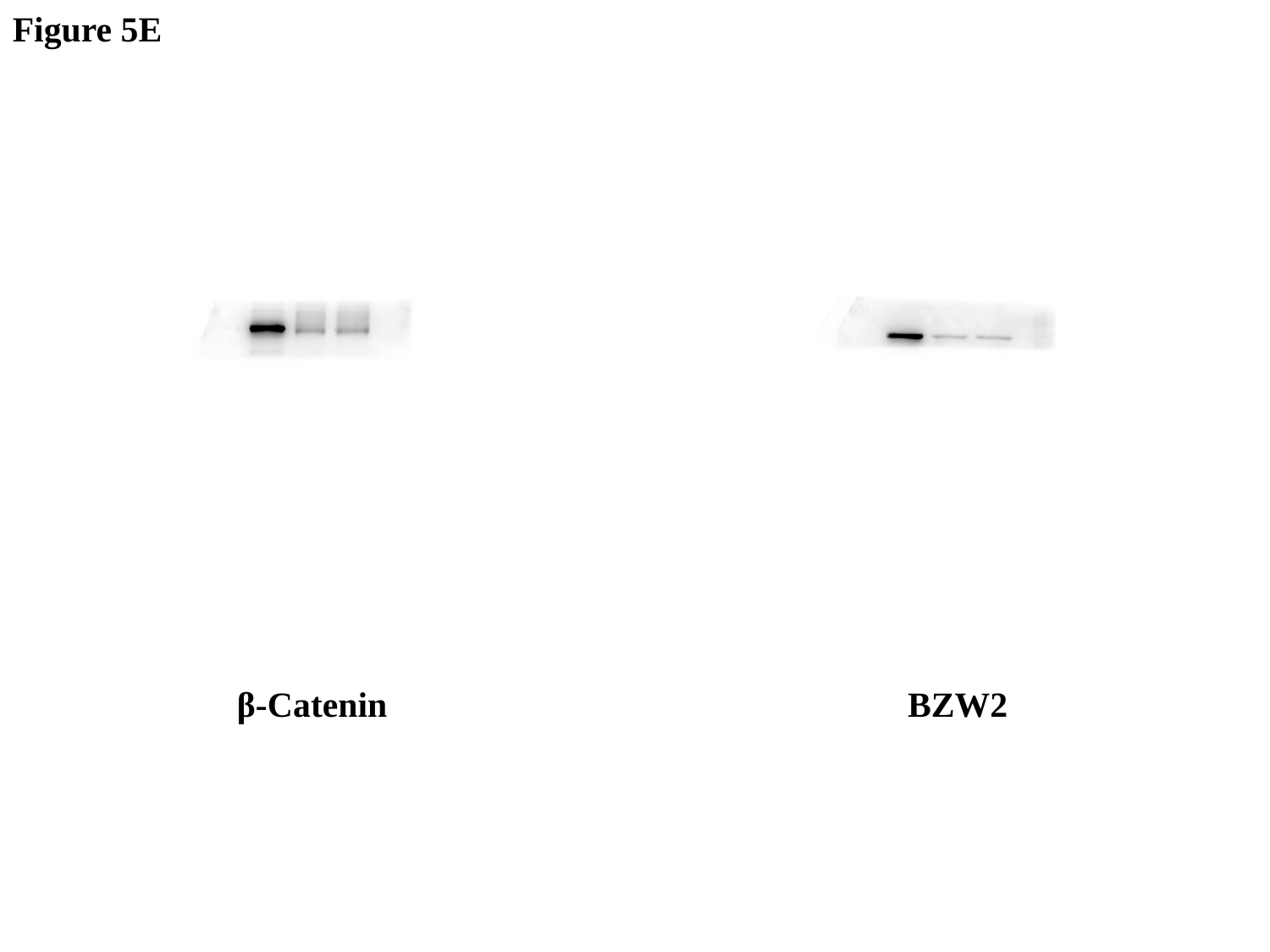

Figure 5E
BZW2
β-Catenin

## Slide 42
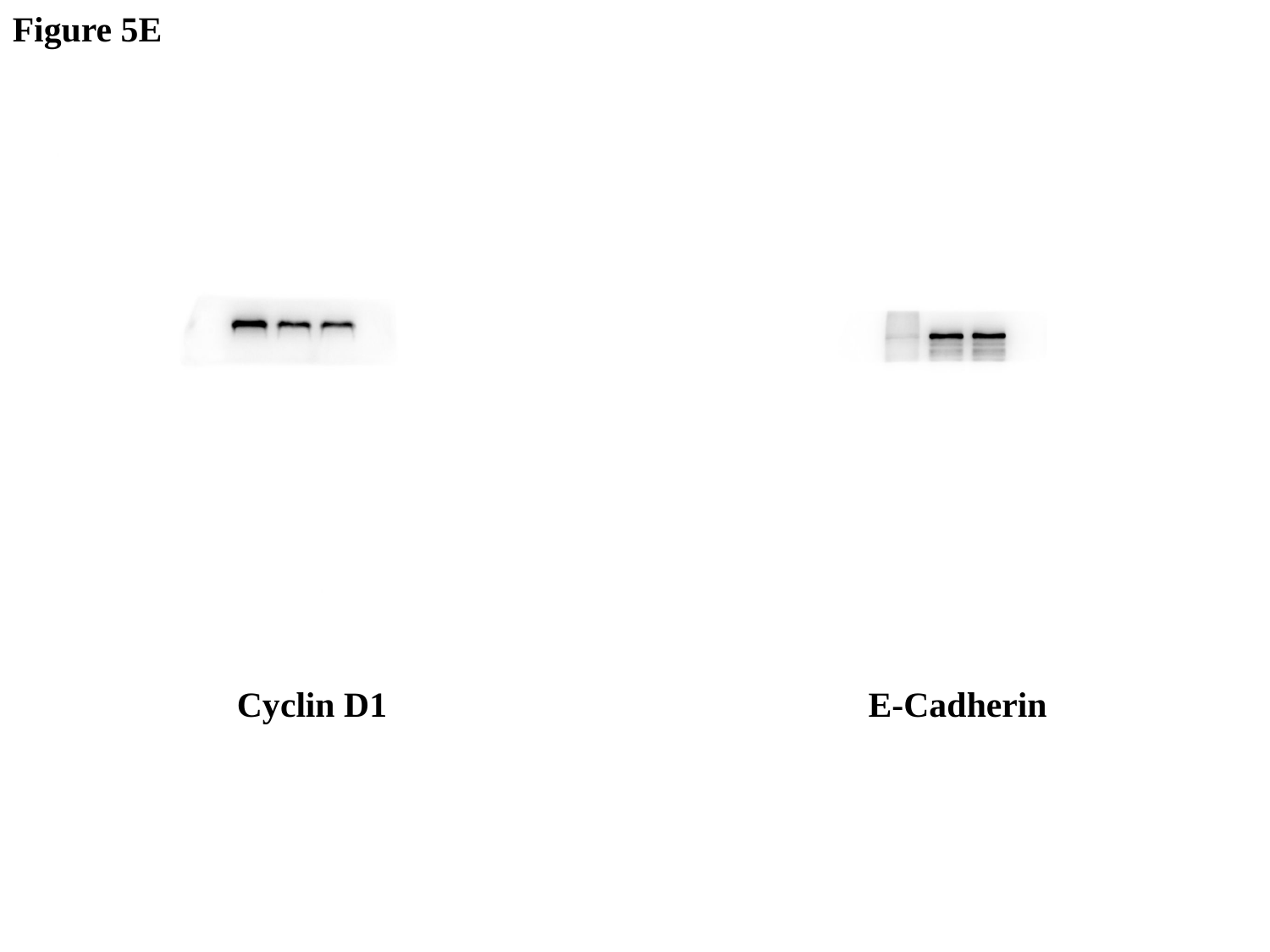

Figure 5E
E-Cadherin
Cyclin D1

## Slide 43
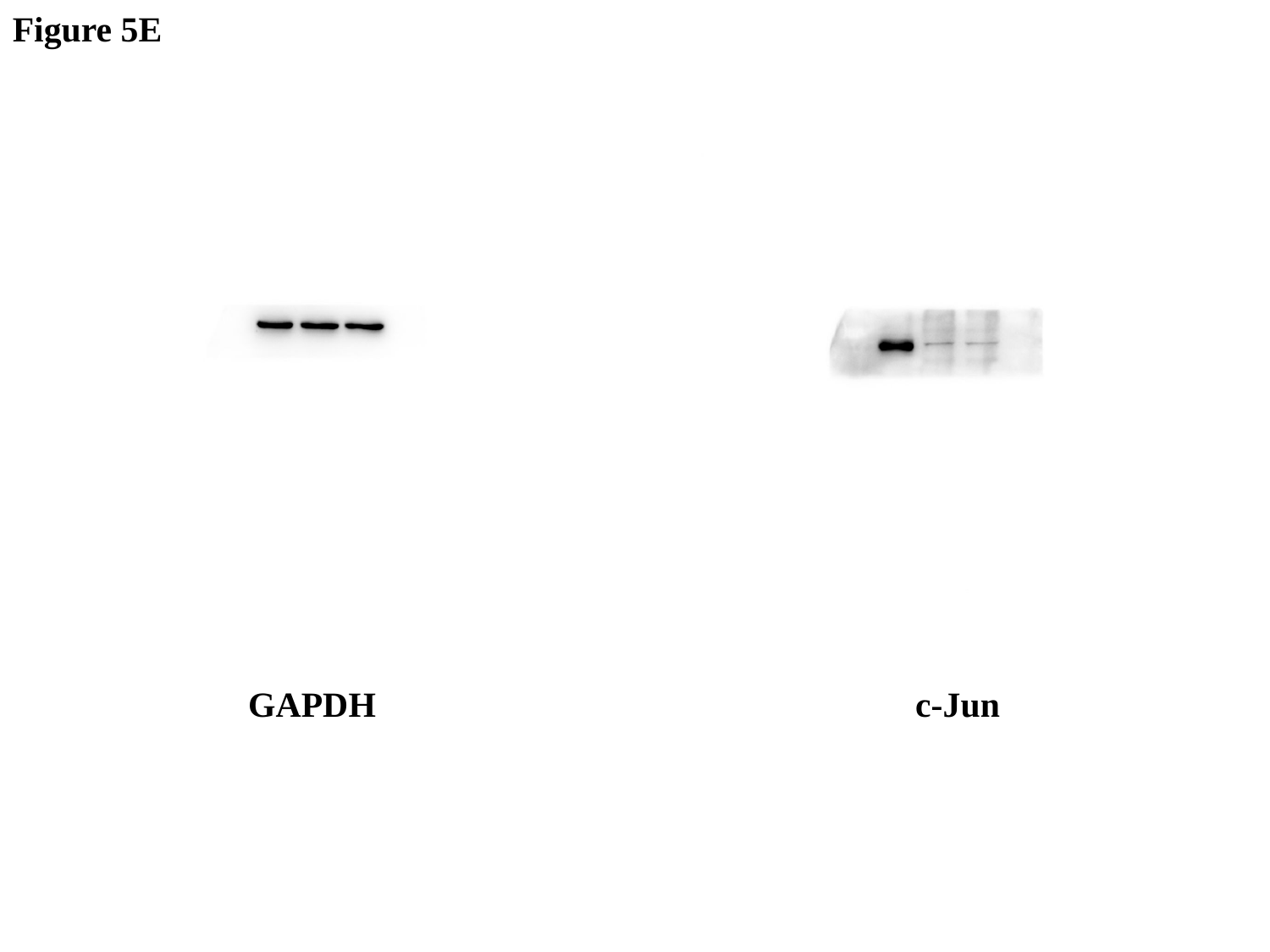

Figure 5E
c-Jun
GAPDH

## Slide 44
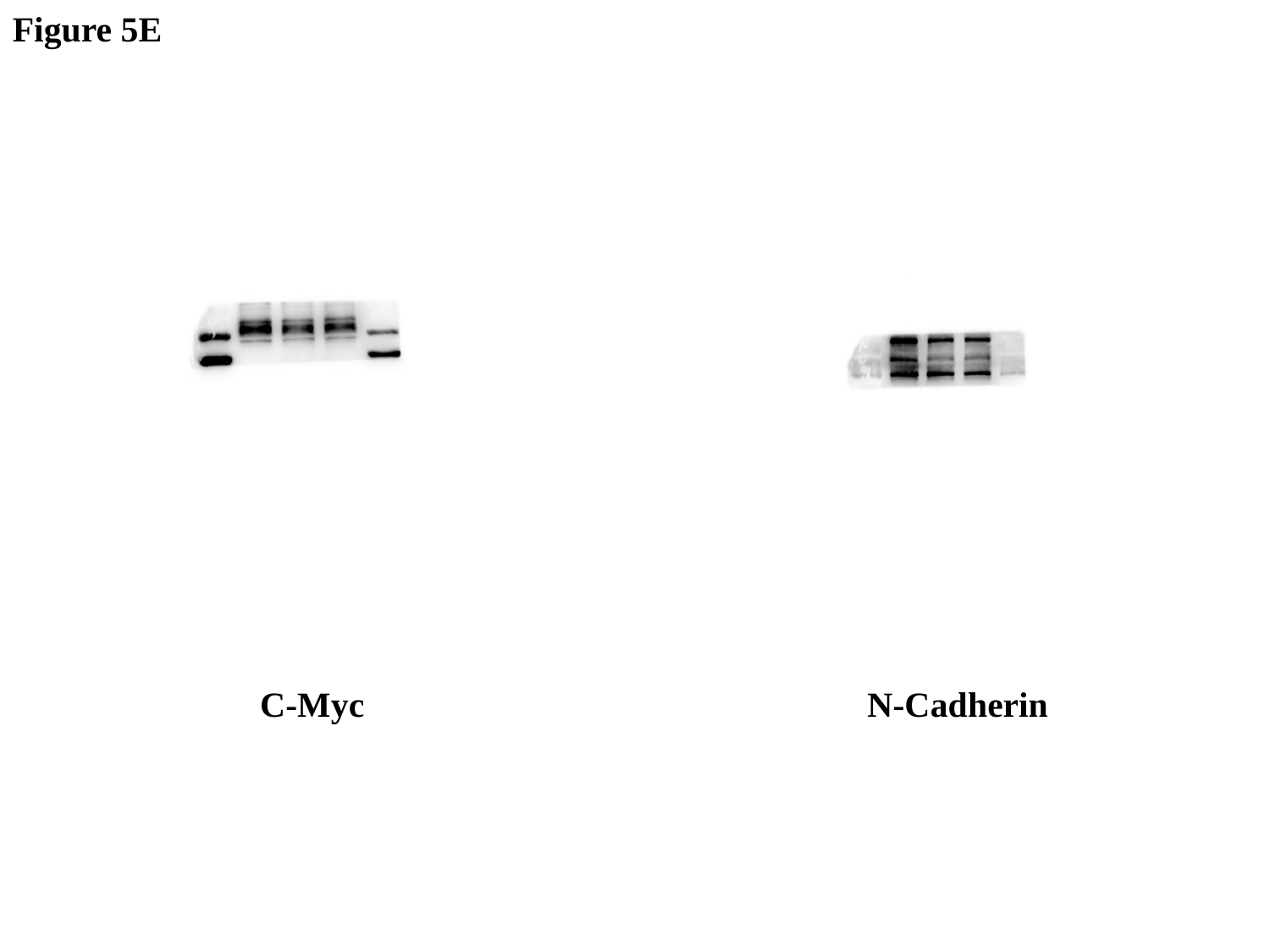

Figure 5E
N-Cadherin
C-Myc

## Slide 45
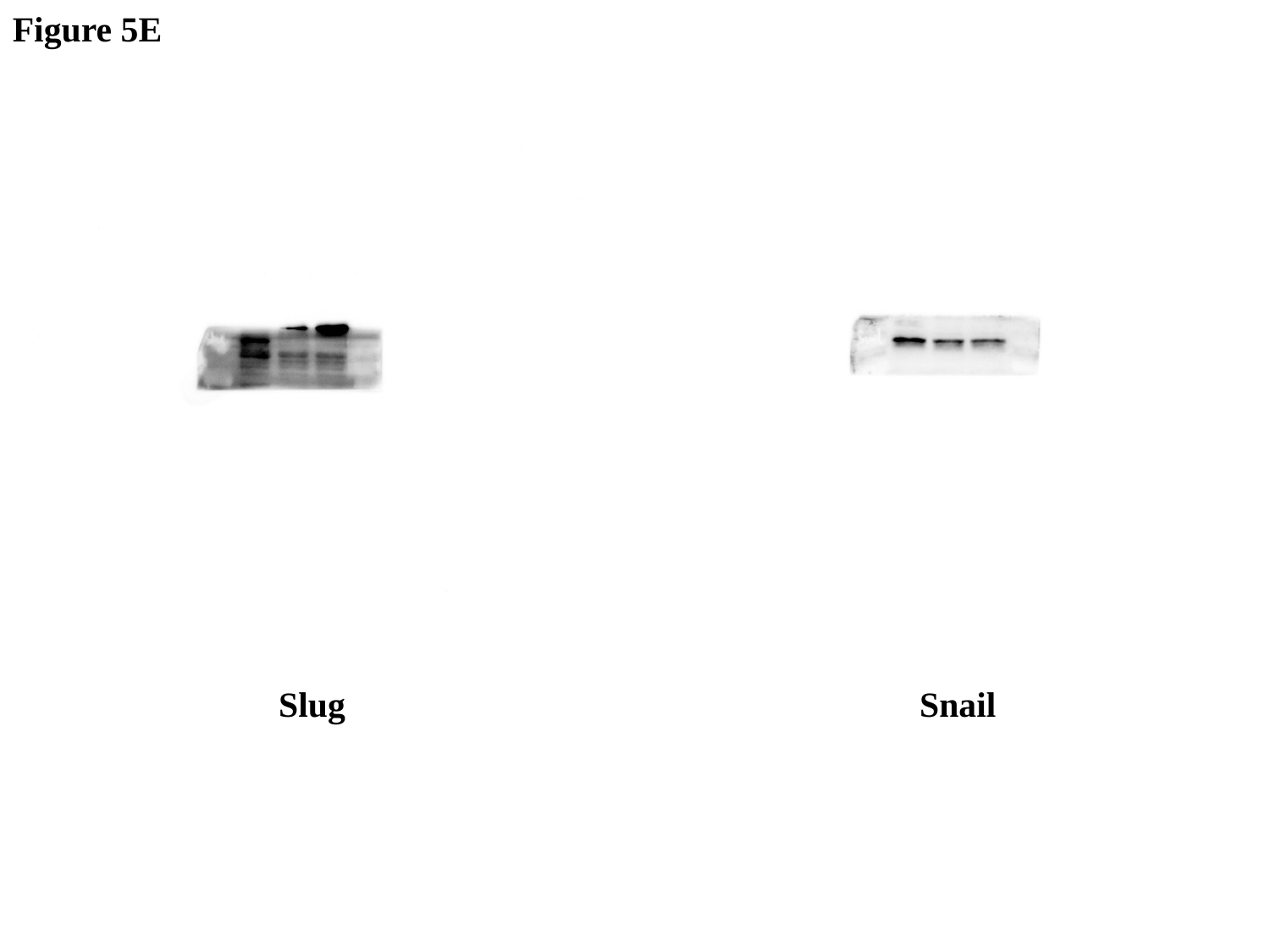

Figure 5E
Snail
Slug

## Slide 46
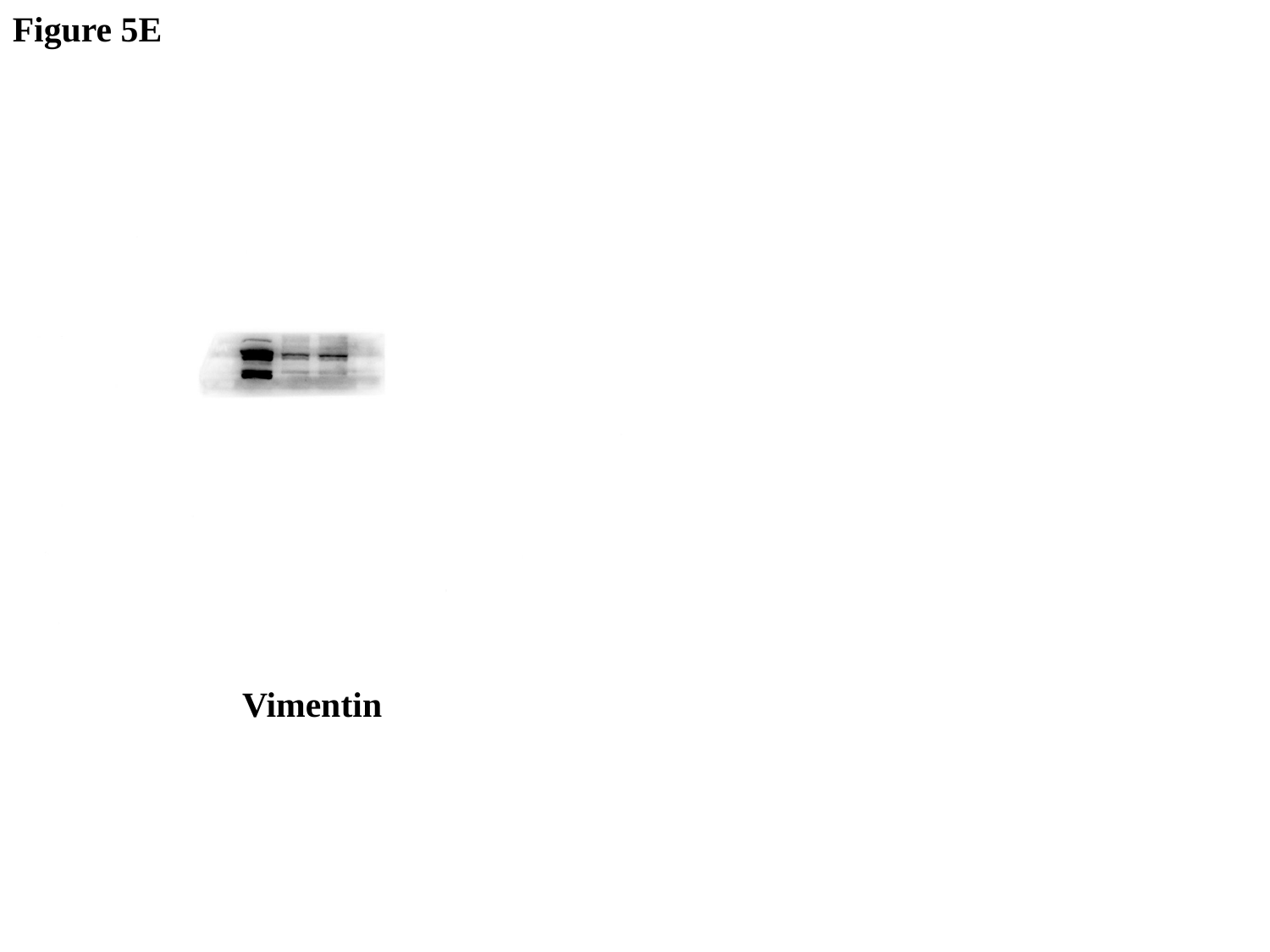

Figure 5E
Vimentin

## Slide 47
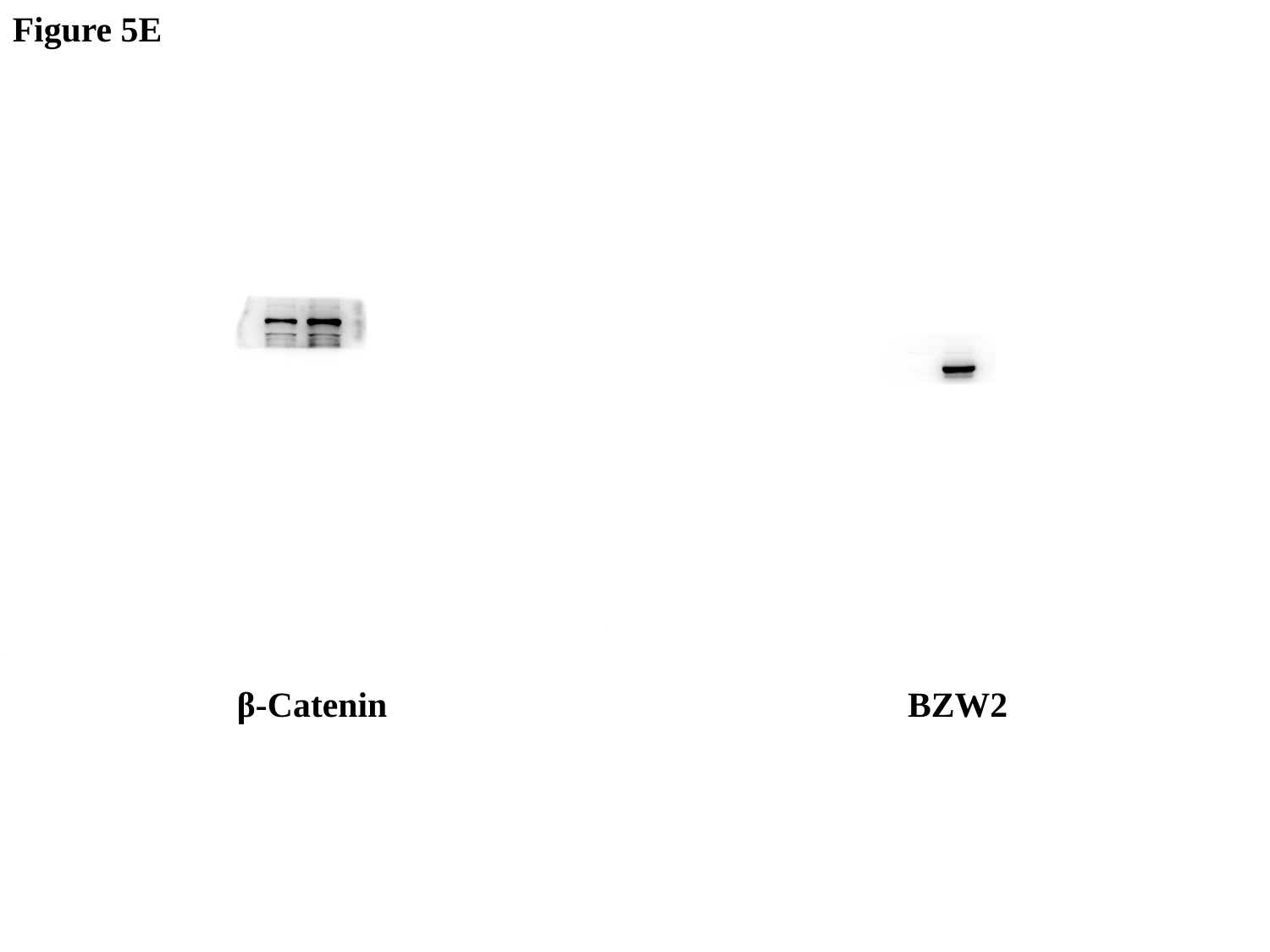

Figure 5E
BZW2
β-Catenin

## Slide 48
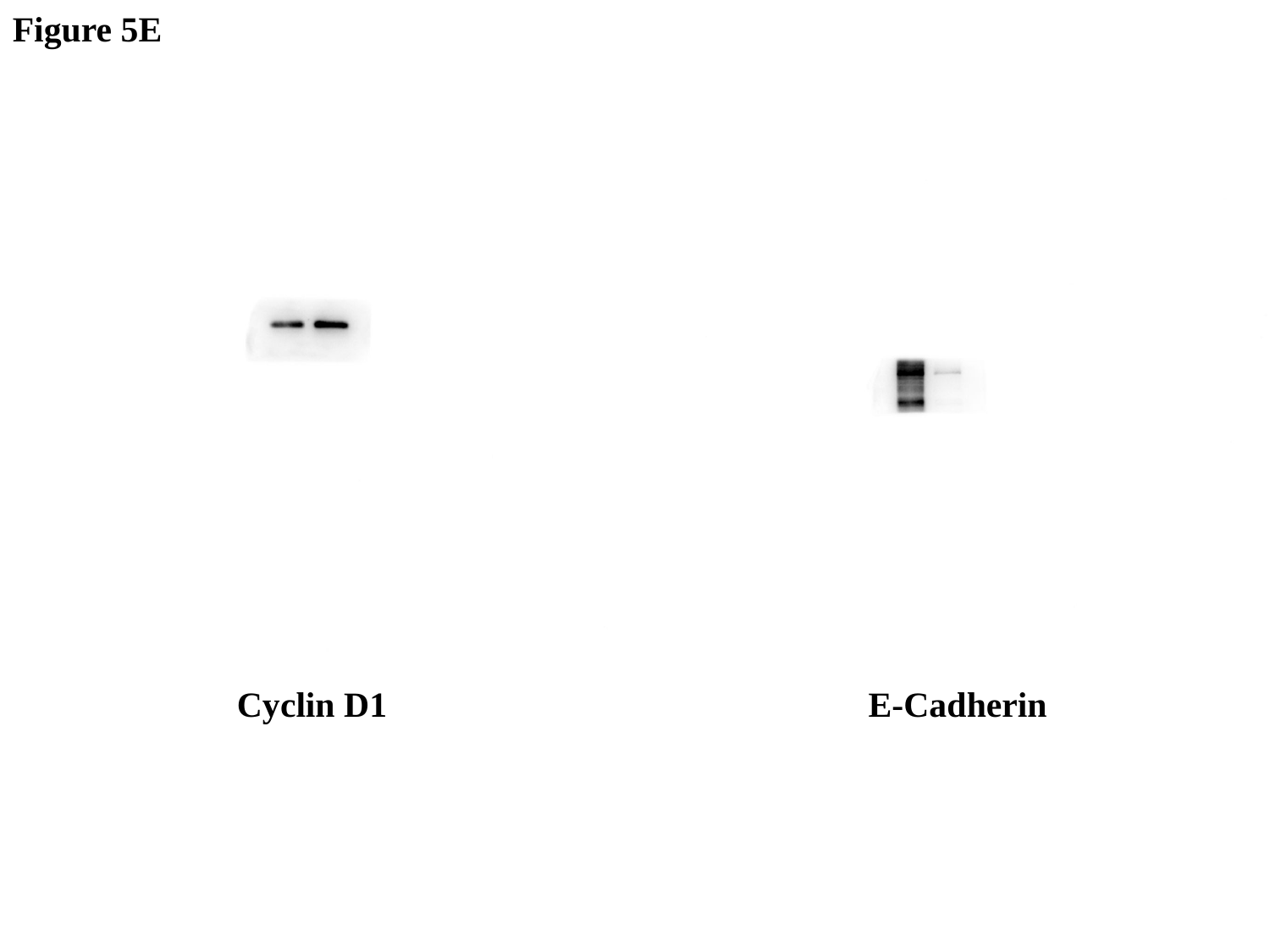

Figure 5E
E-Cadherin
Cyclin D1

## Slide 49
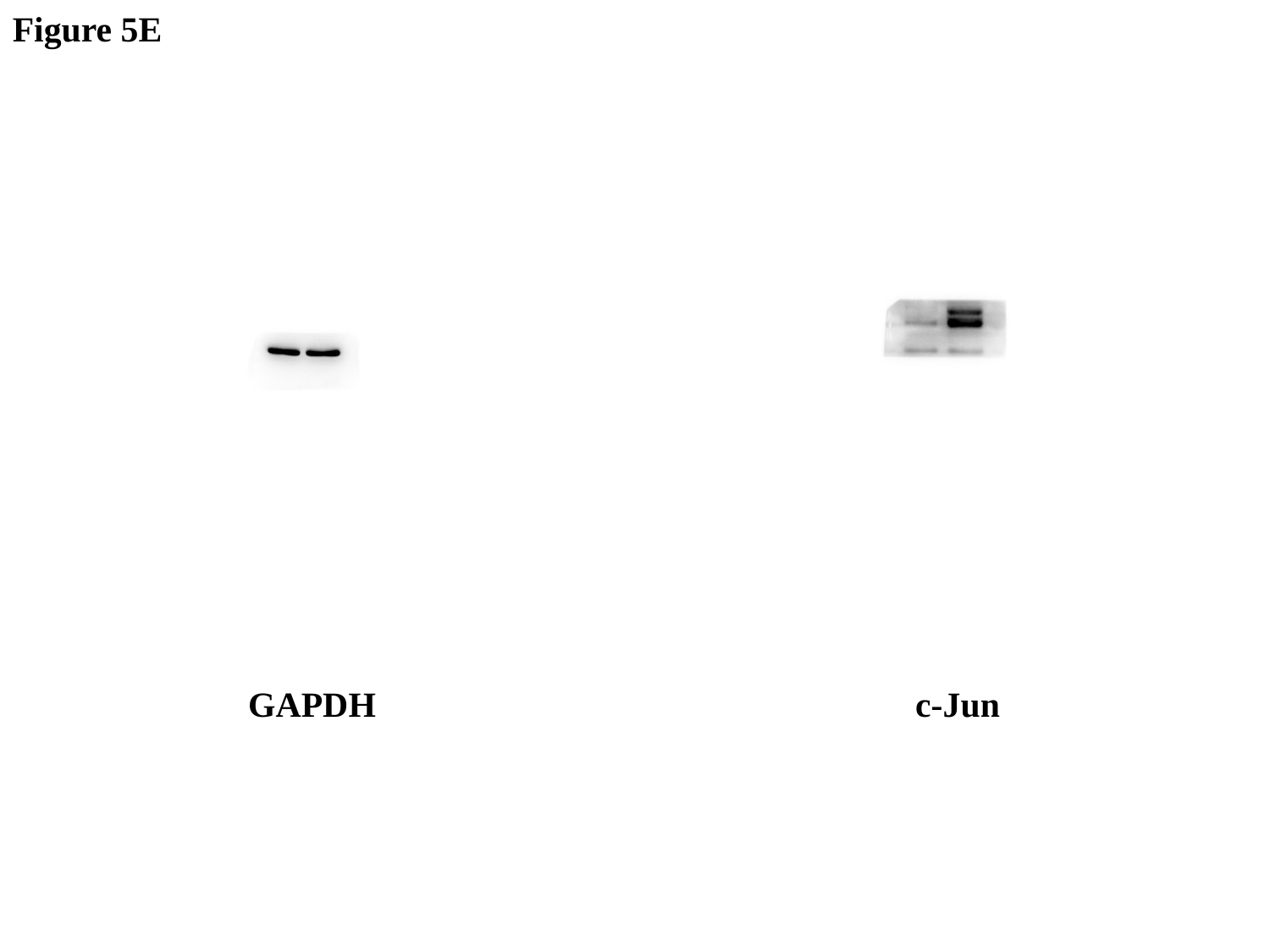

Figure 5E
c-Jun
GAPDH

## Slide 50
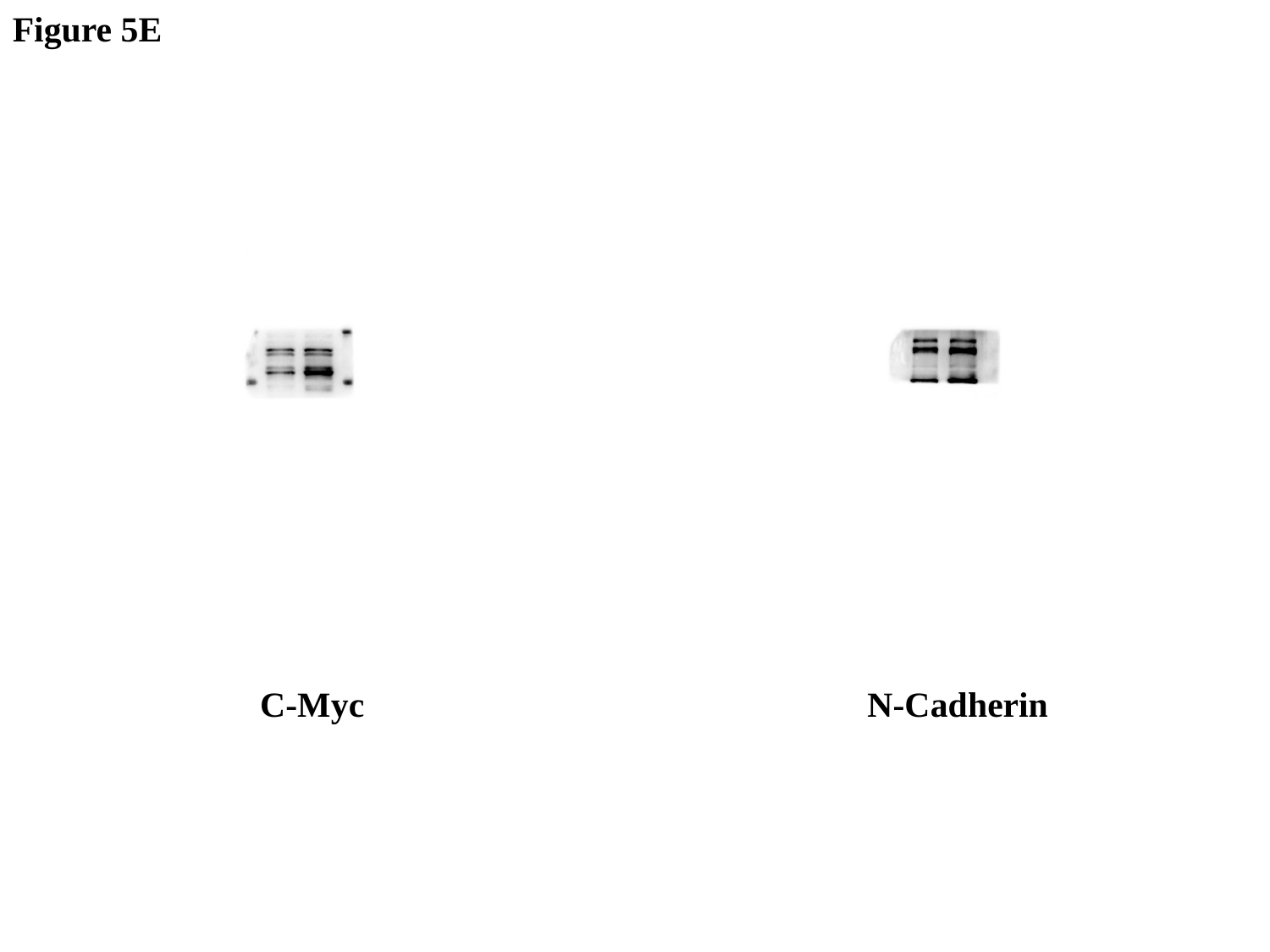

Figure 5E
N-Cadherin
C-Myc

## Slide 51
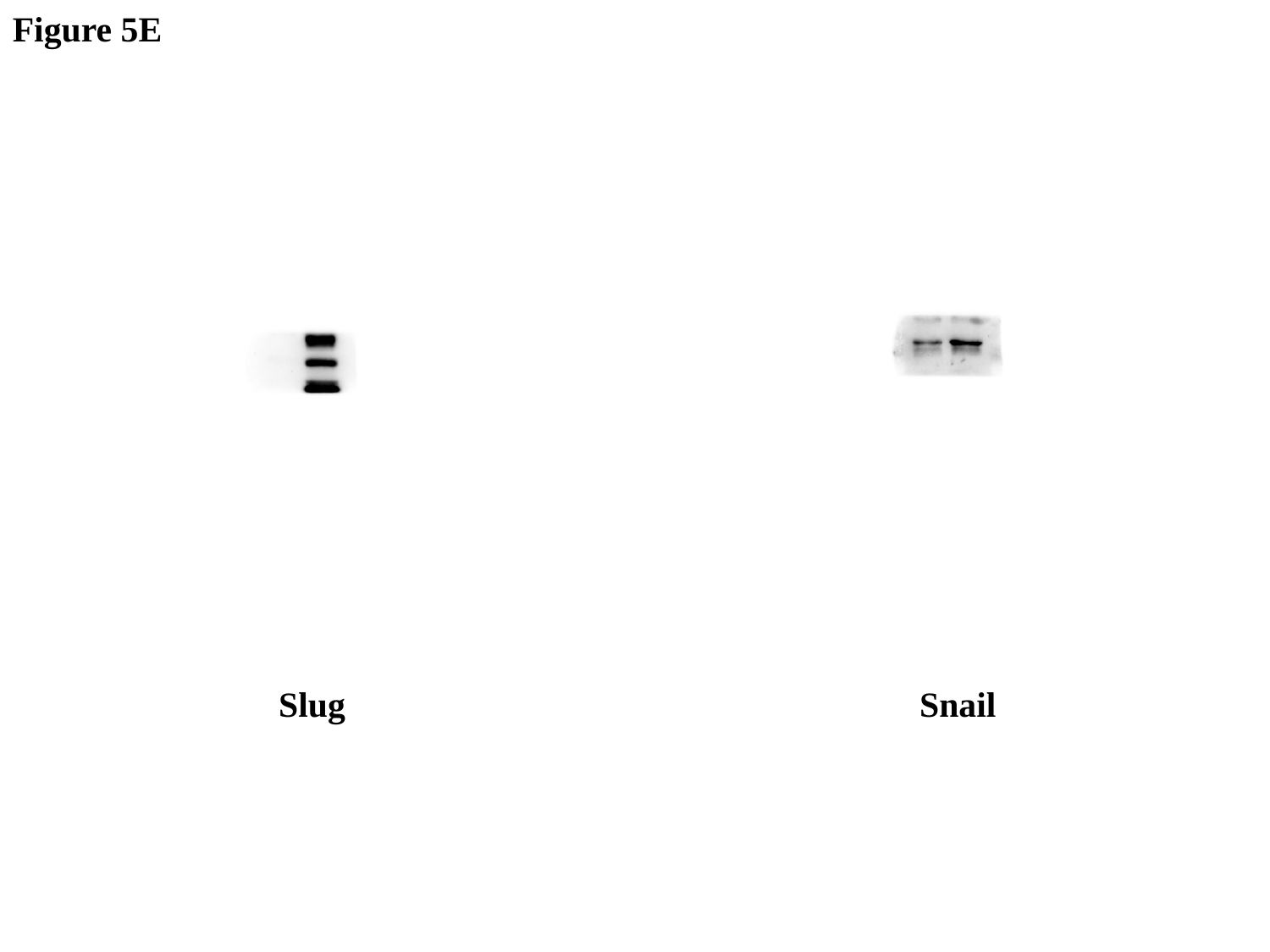

Figure 5E
Snail
Slug

## Slide 52
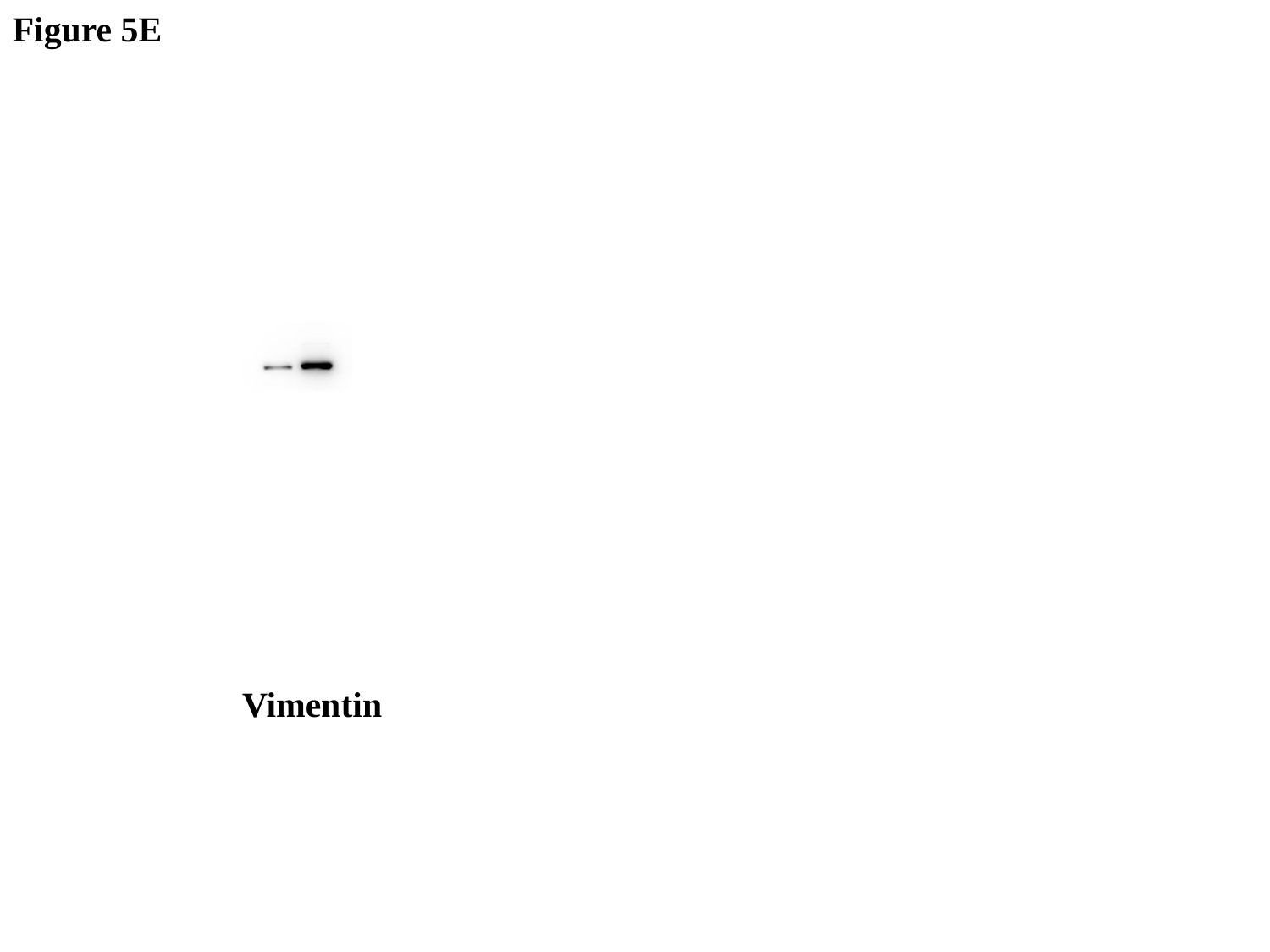

Figure 5E
Vimentin

## Slide 53
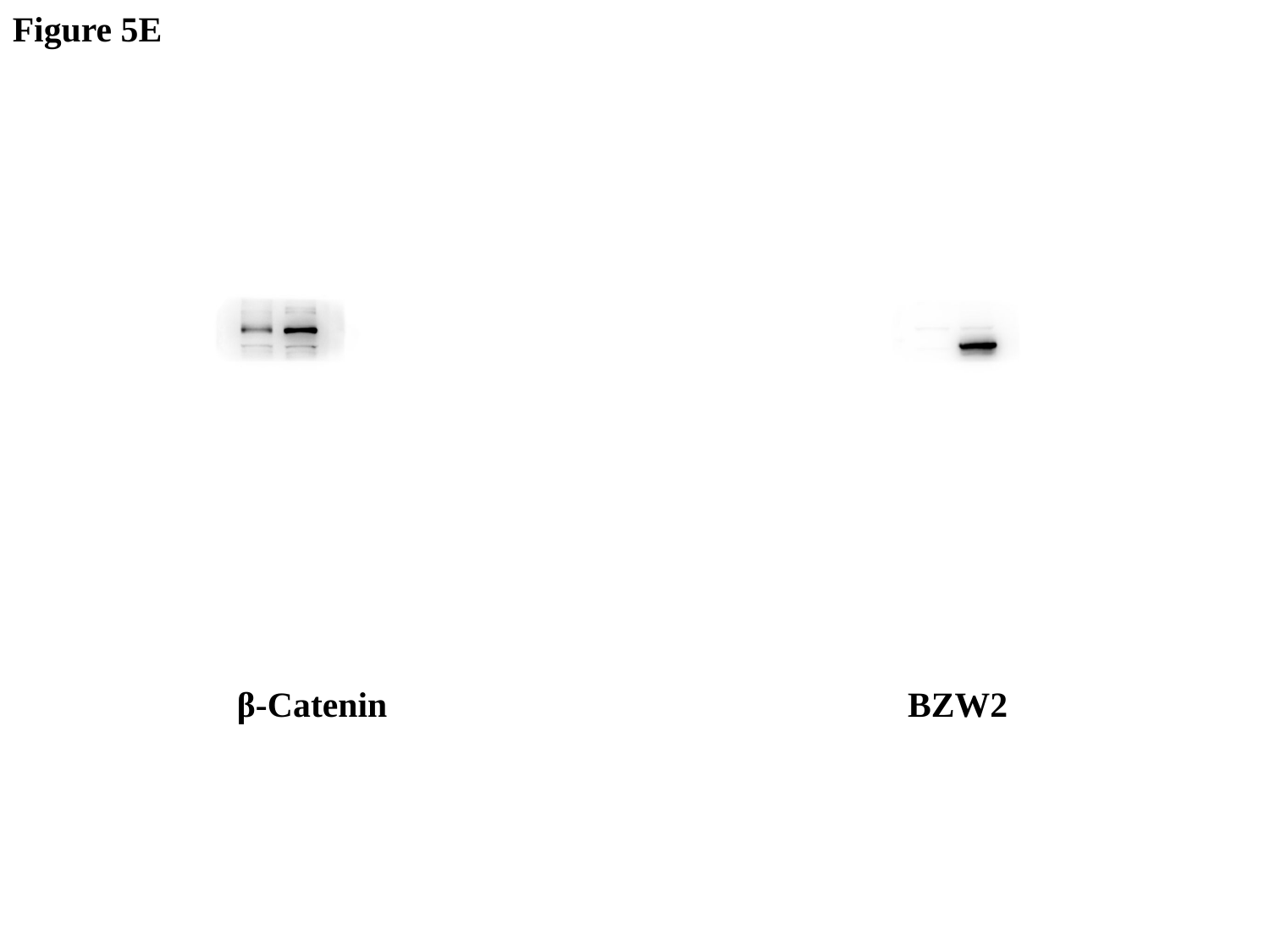

Figure 5E
BZW2
β-Catenin

## Slide 54
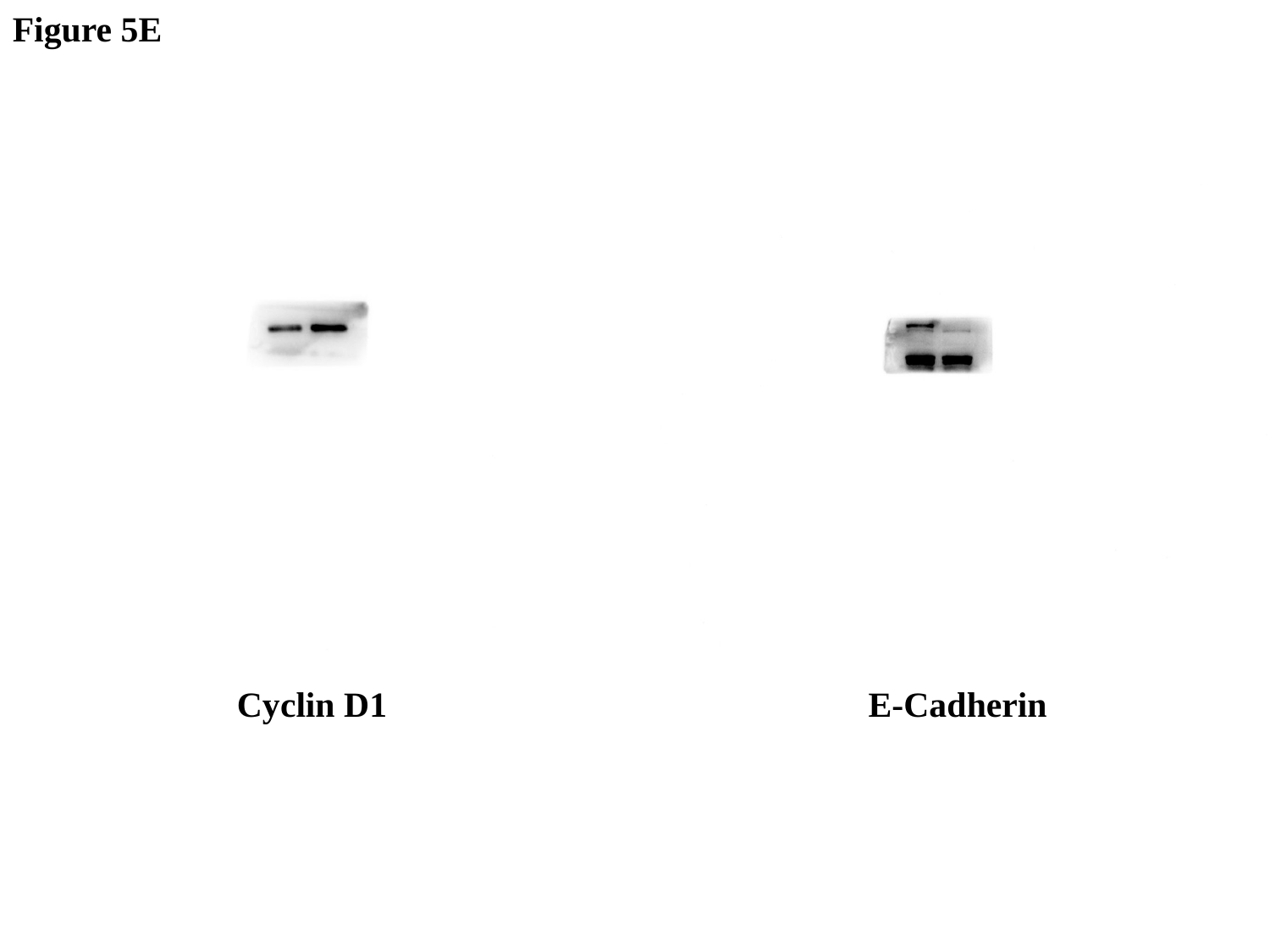

Figure 5E
E-Cadherin
Cyclin D1

## Slide 55
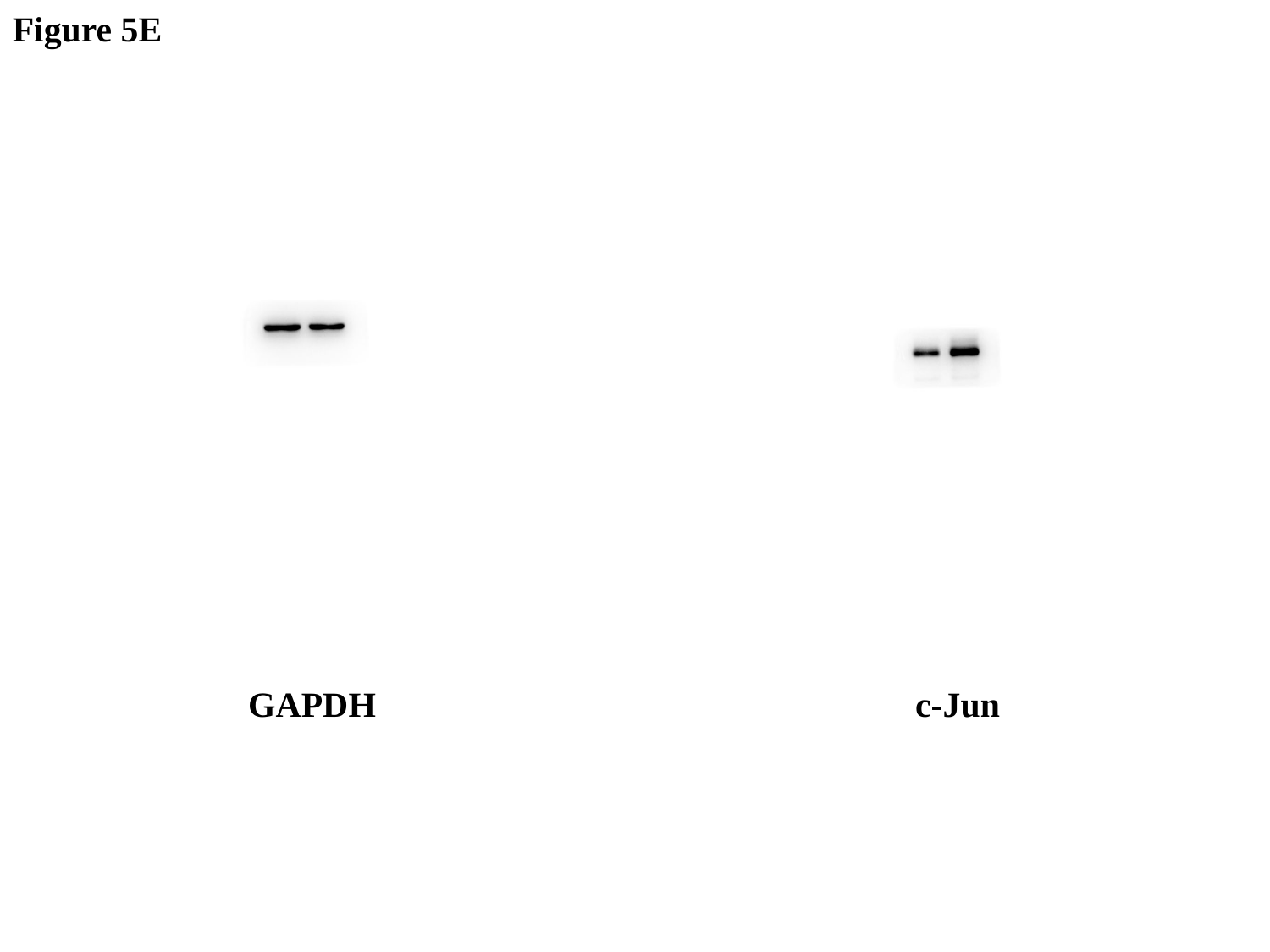

Figure 5E
c-Jun
GAPDH

## Slide 56
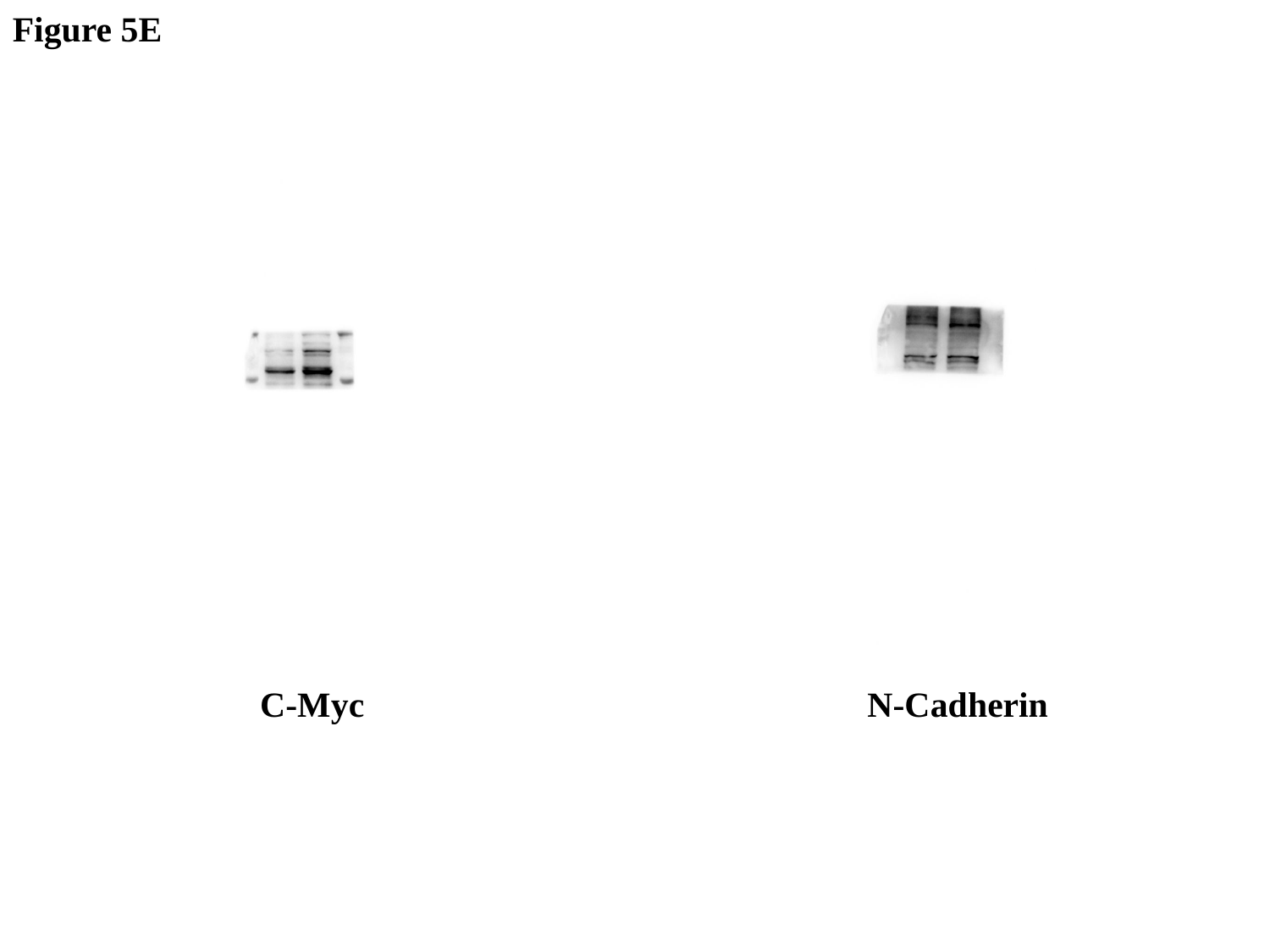

Figure 5E
N-Cadherin
C-Myc

## Slide 57
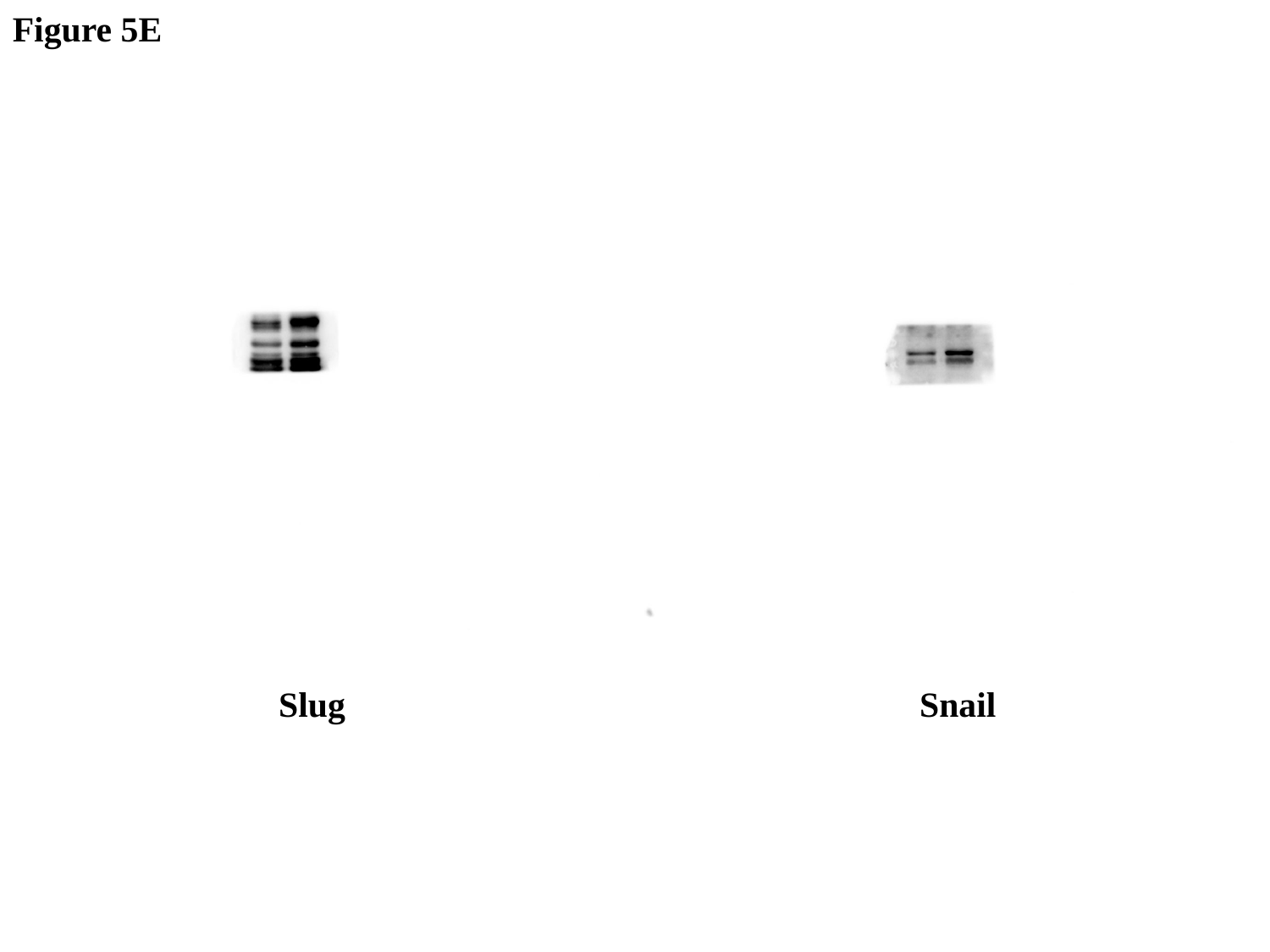

Figure 5E
Snail
Slug

## Slide 58
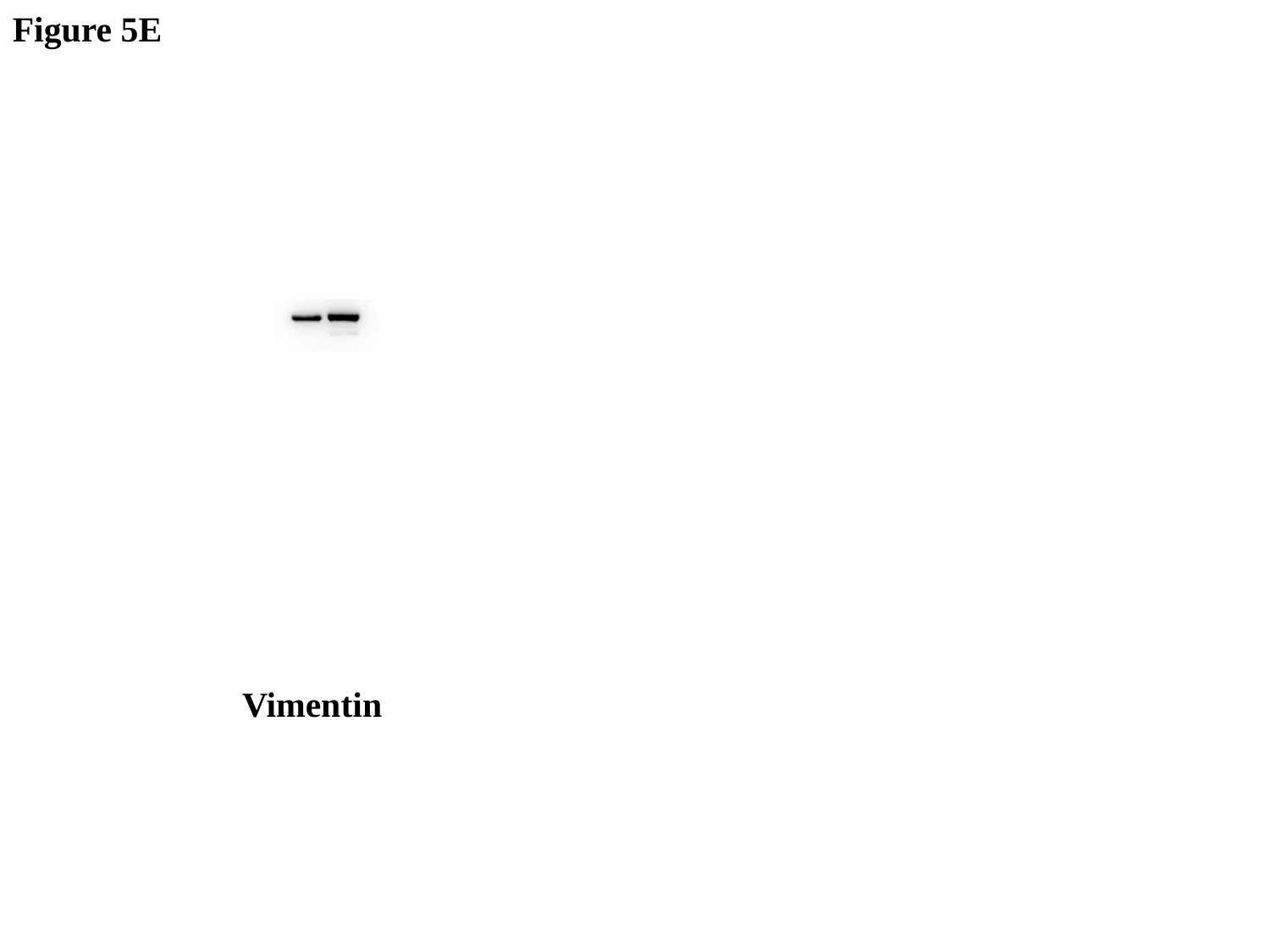

Figure 5E
Vimentin

## Slide 59
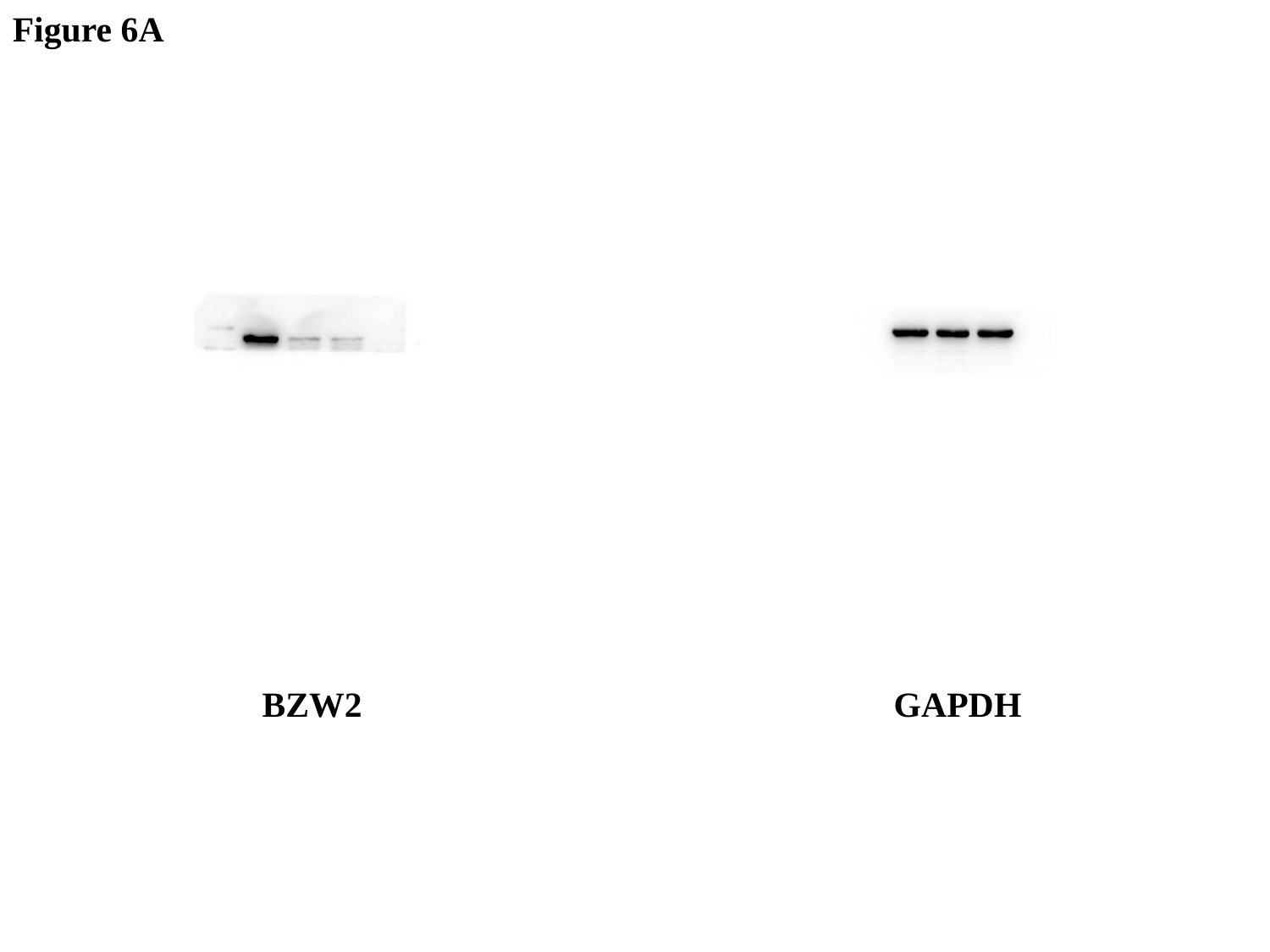

Figure 6A
GAPDH
BZW2

## Slide 60
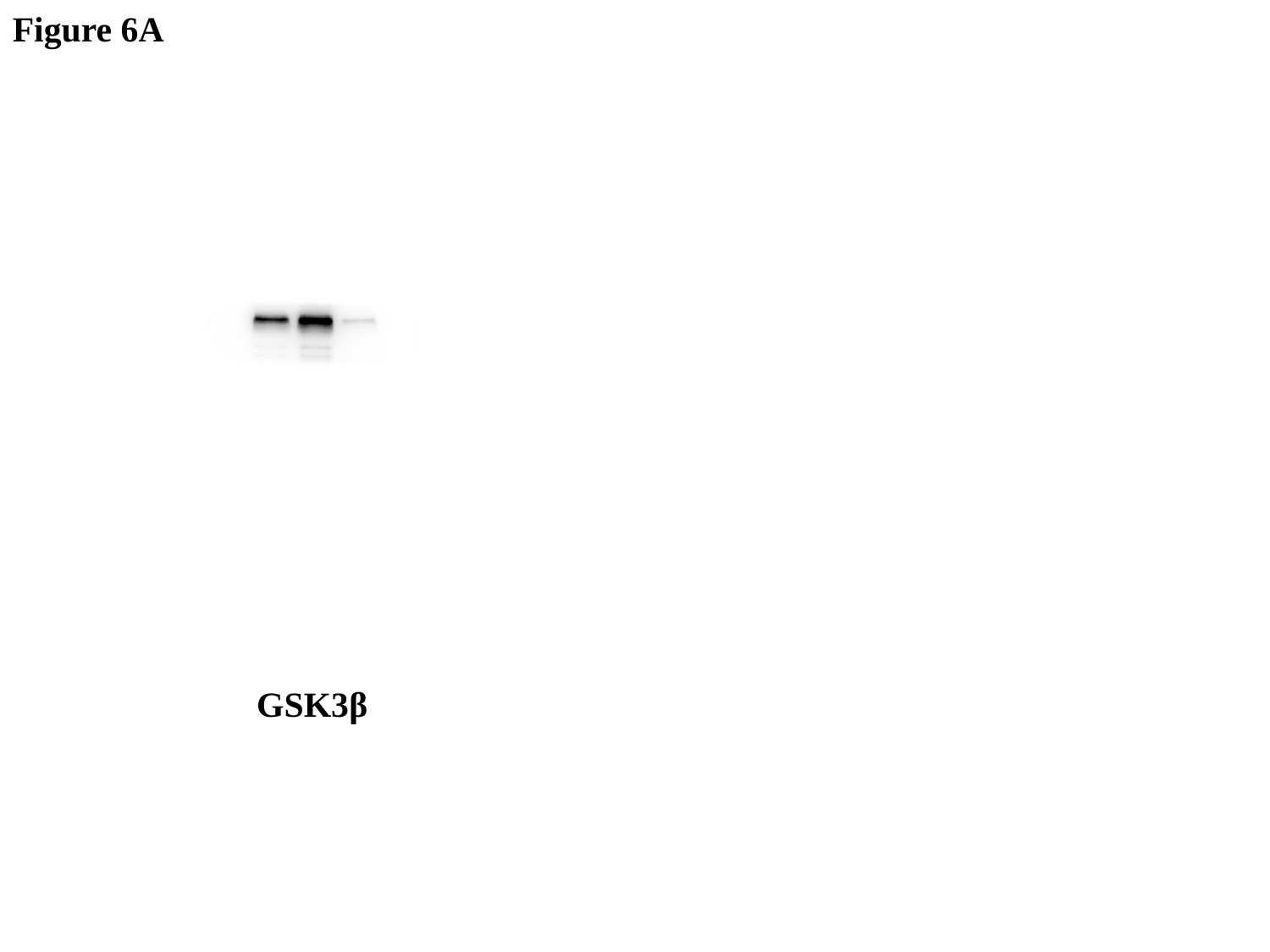

Figure 6A
GSK3β

## Slide 61
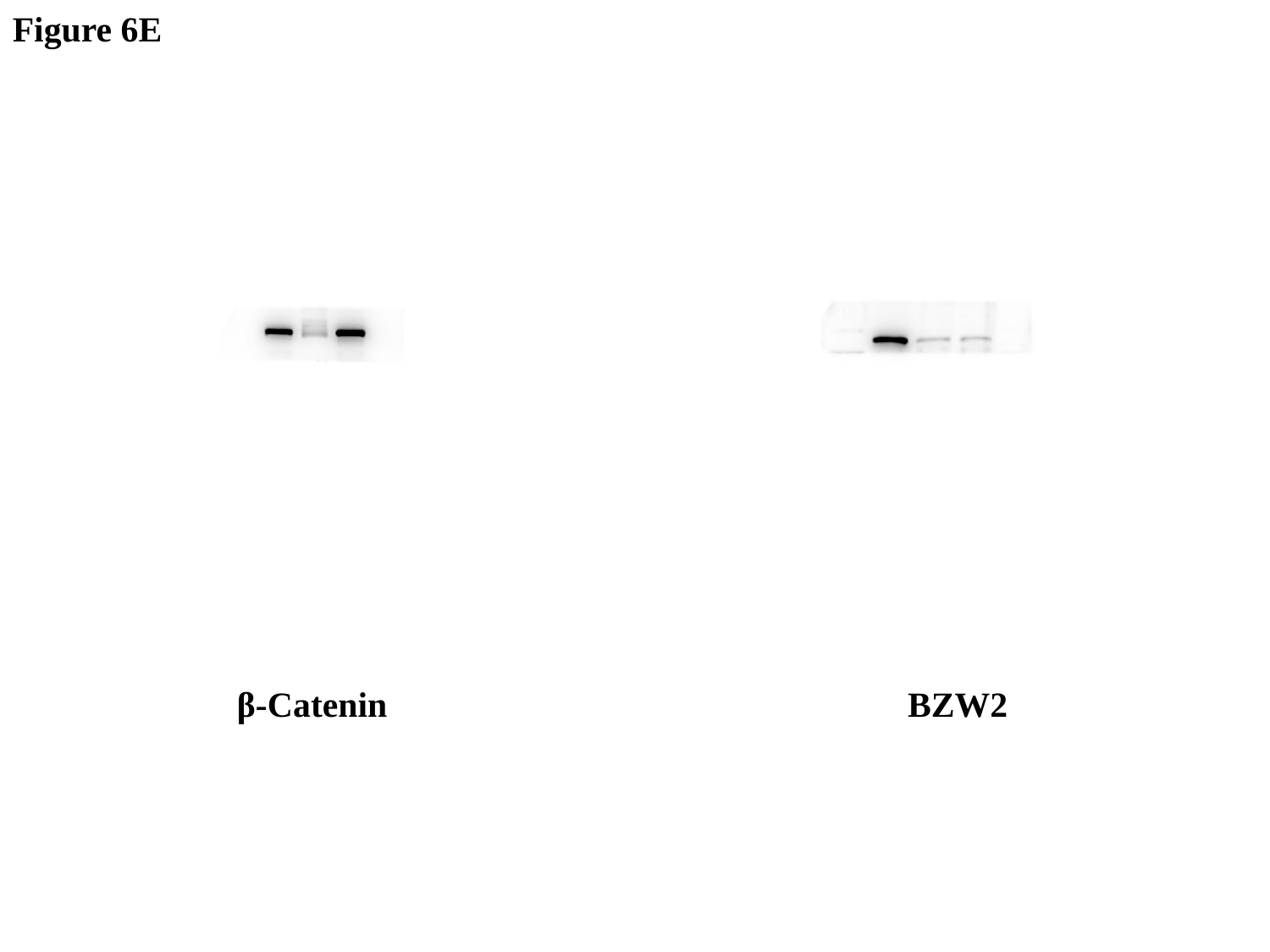

Figure 6E
BZW2
β-Catenin

## Slide 62
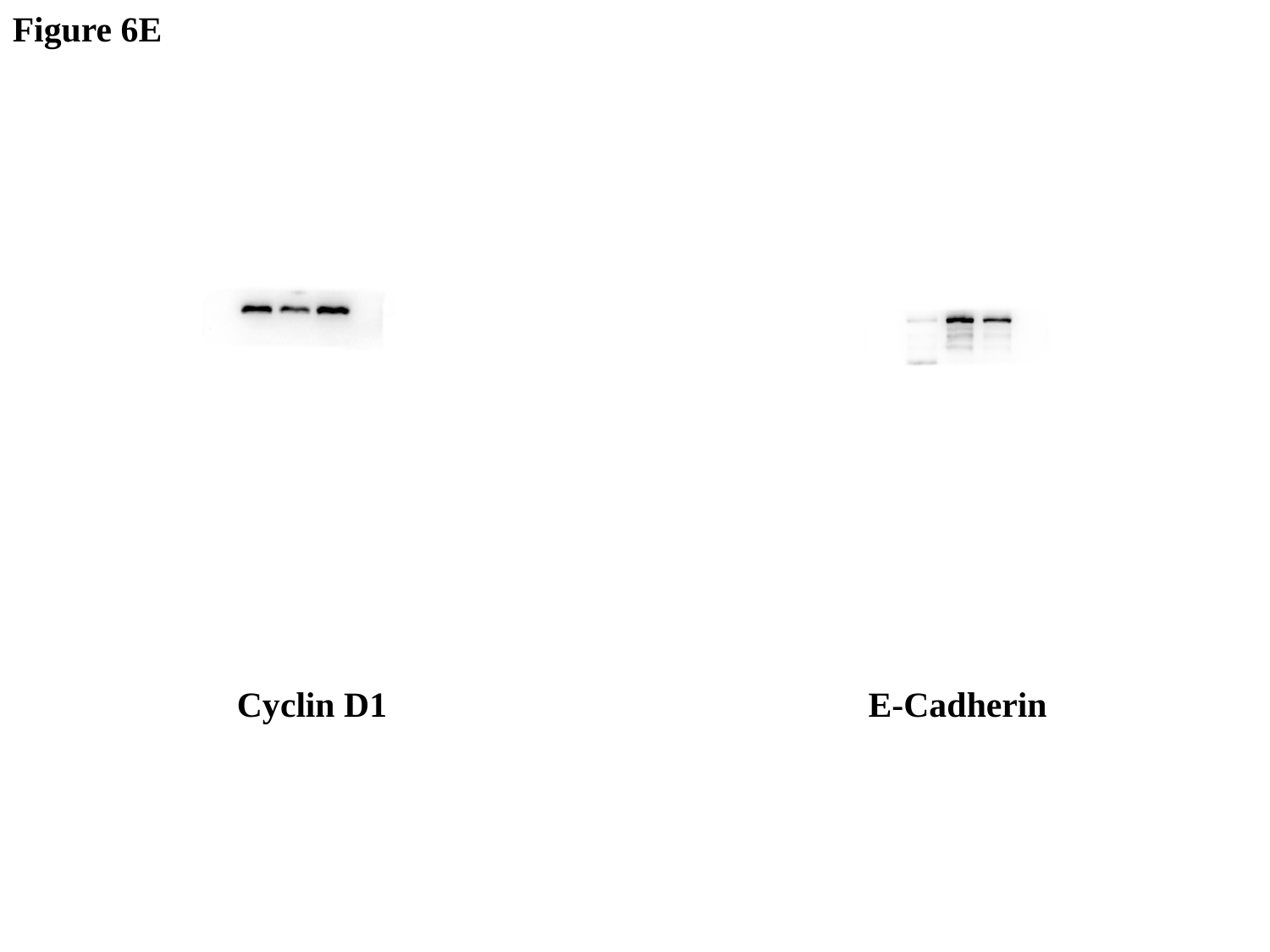

Figure 6E
E-Cadherin
Cyclin D1

## Slide 63
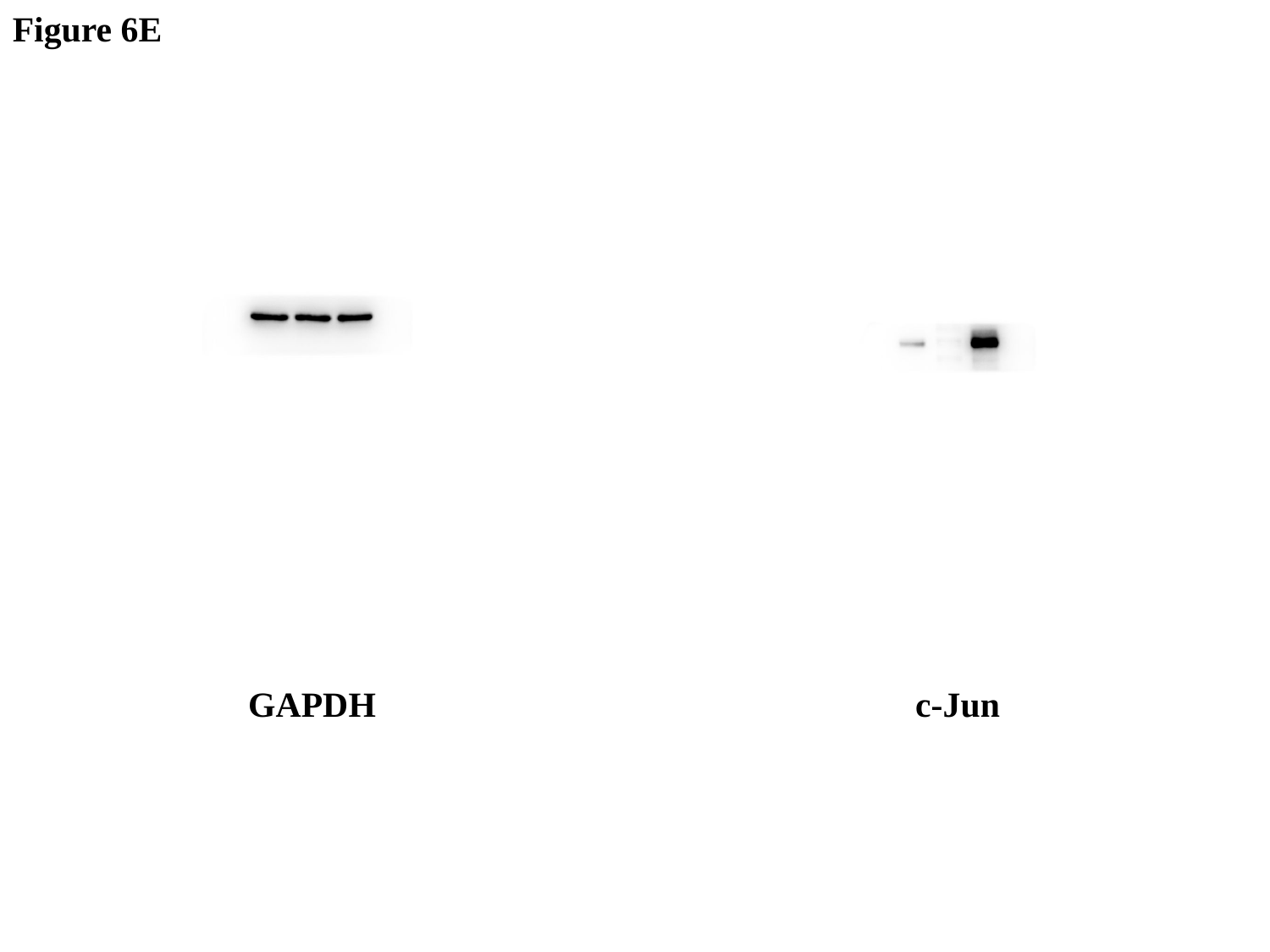

Figure 6E
c-Jun
GAPDH

## Slide 64
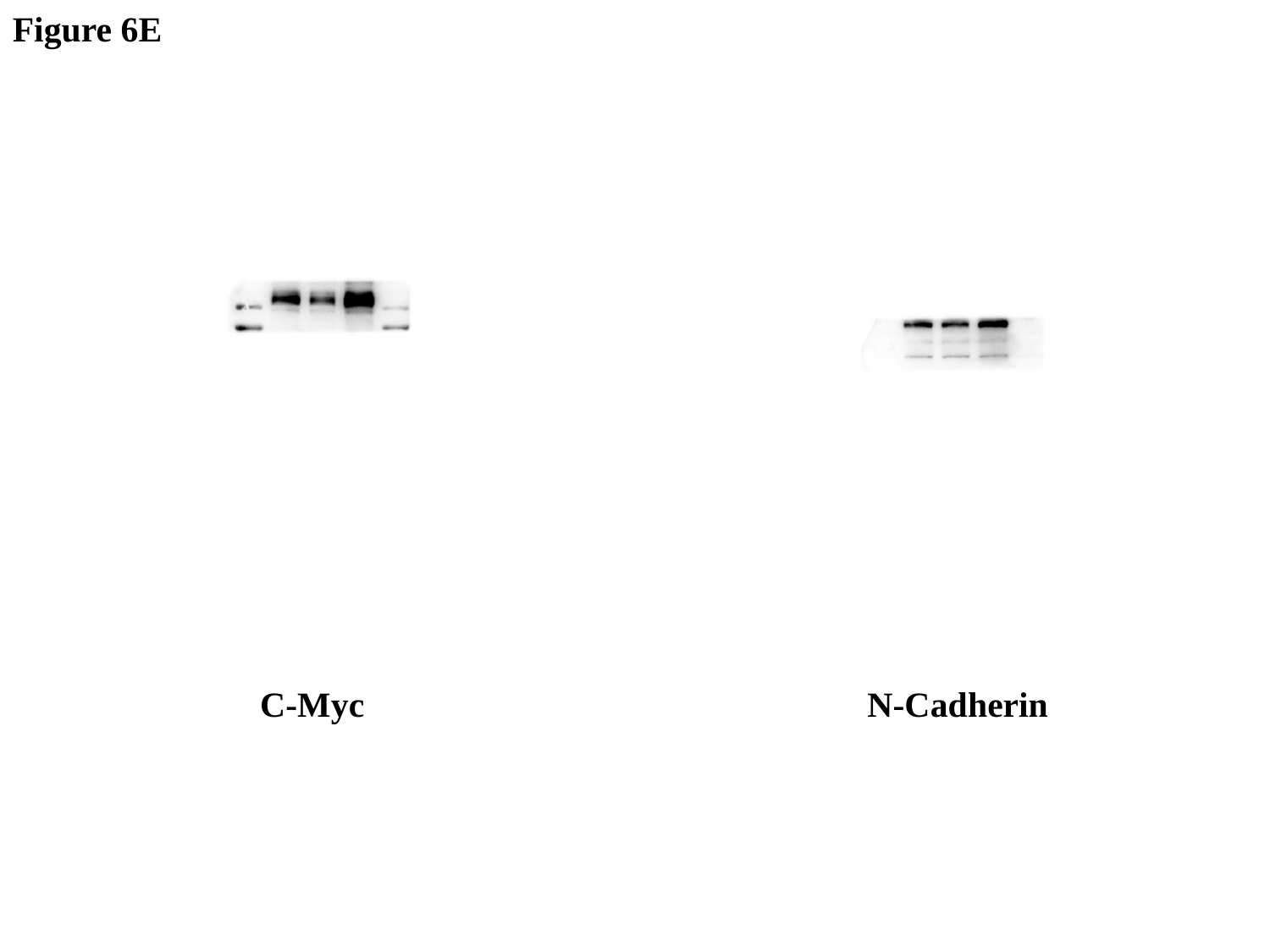

Figure 6E
N-Cadherin
C-Myc

## Slide 65
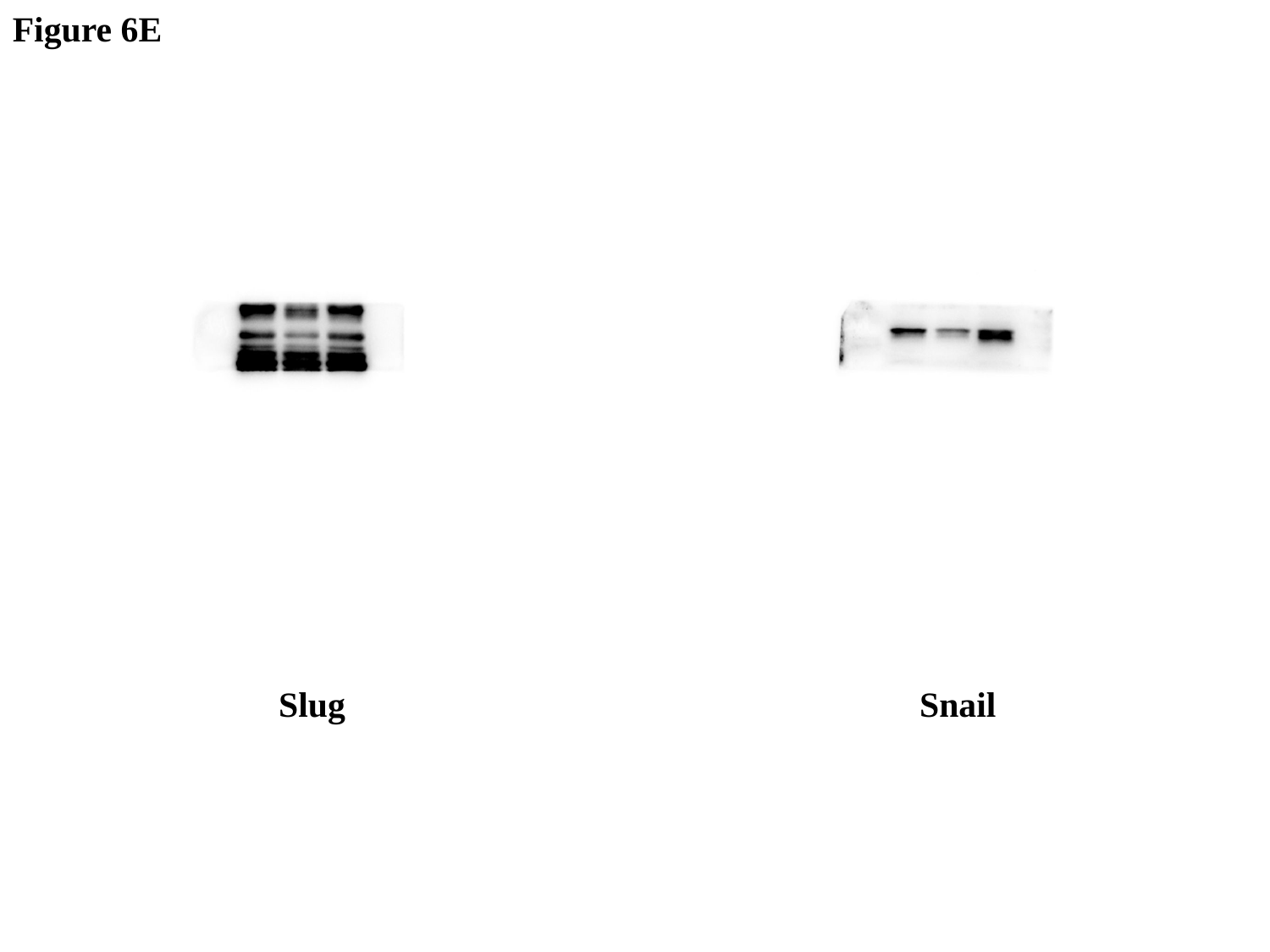

Figure 6E
Snail
Slug

## Slide 66
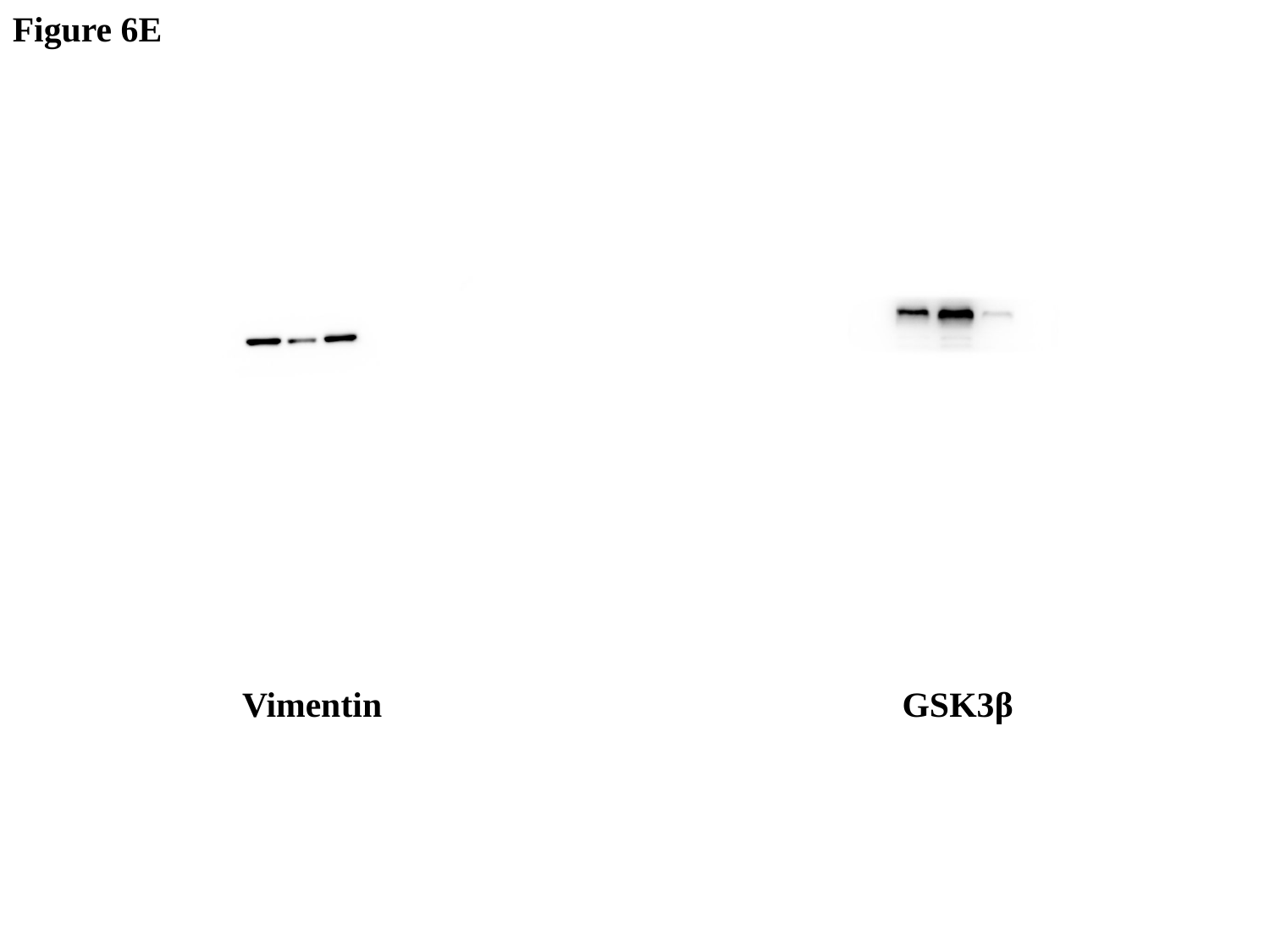

Figure 6E
GSK3β
Vimentin

## Slide 67
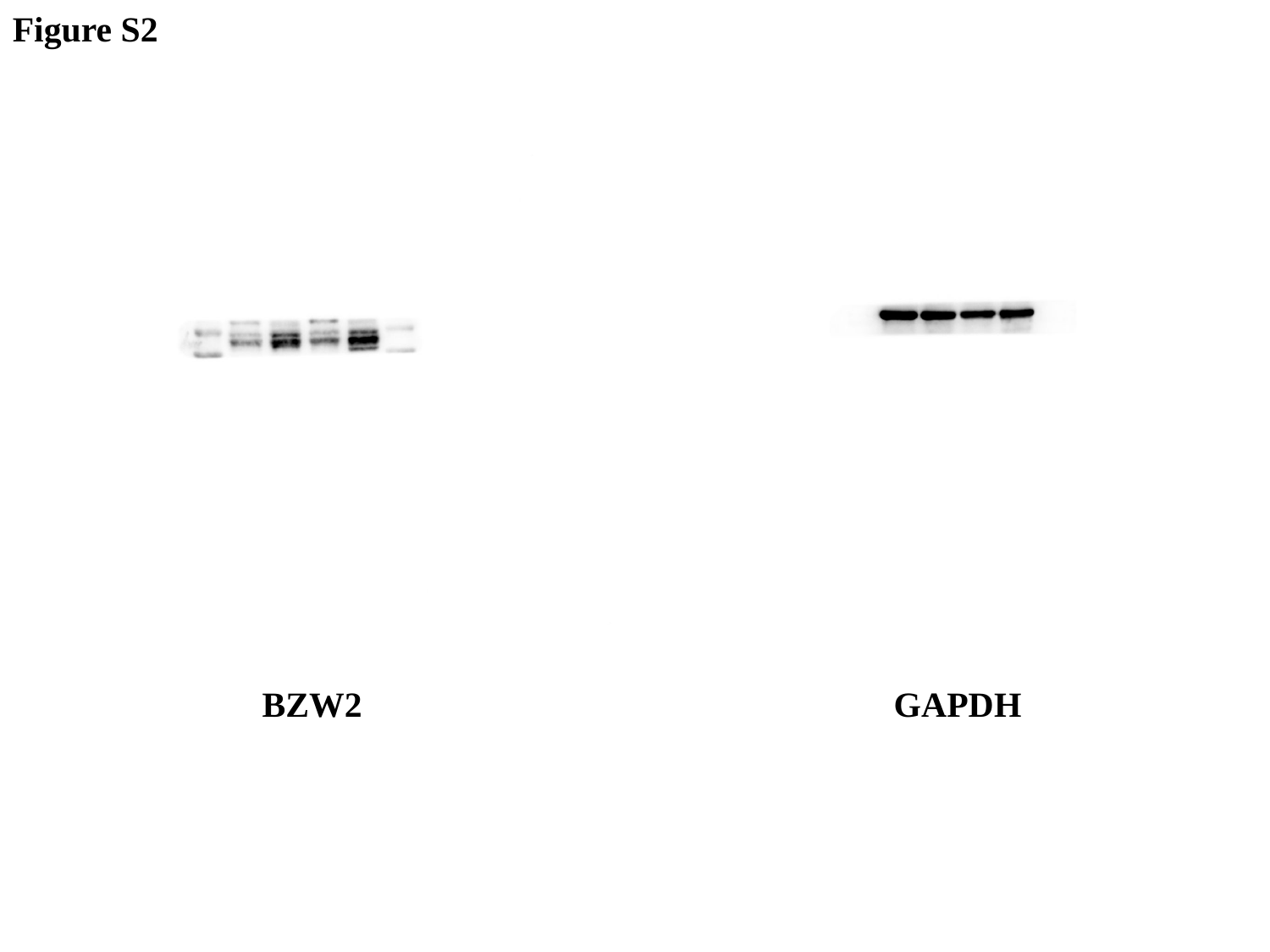

Figure S2
GAPDH
BZW2

## Slide 68
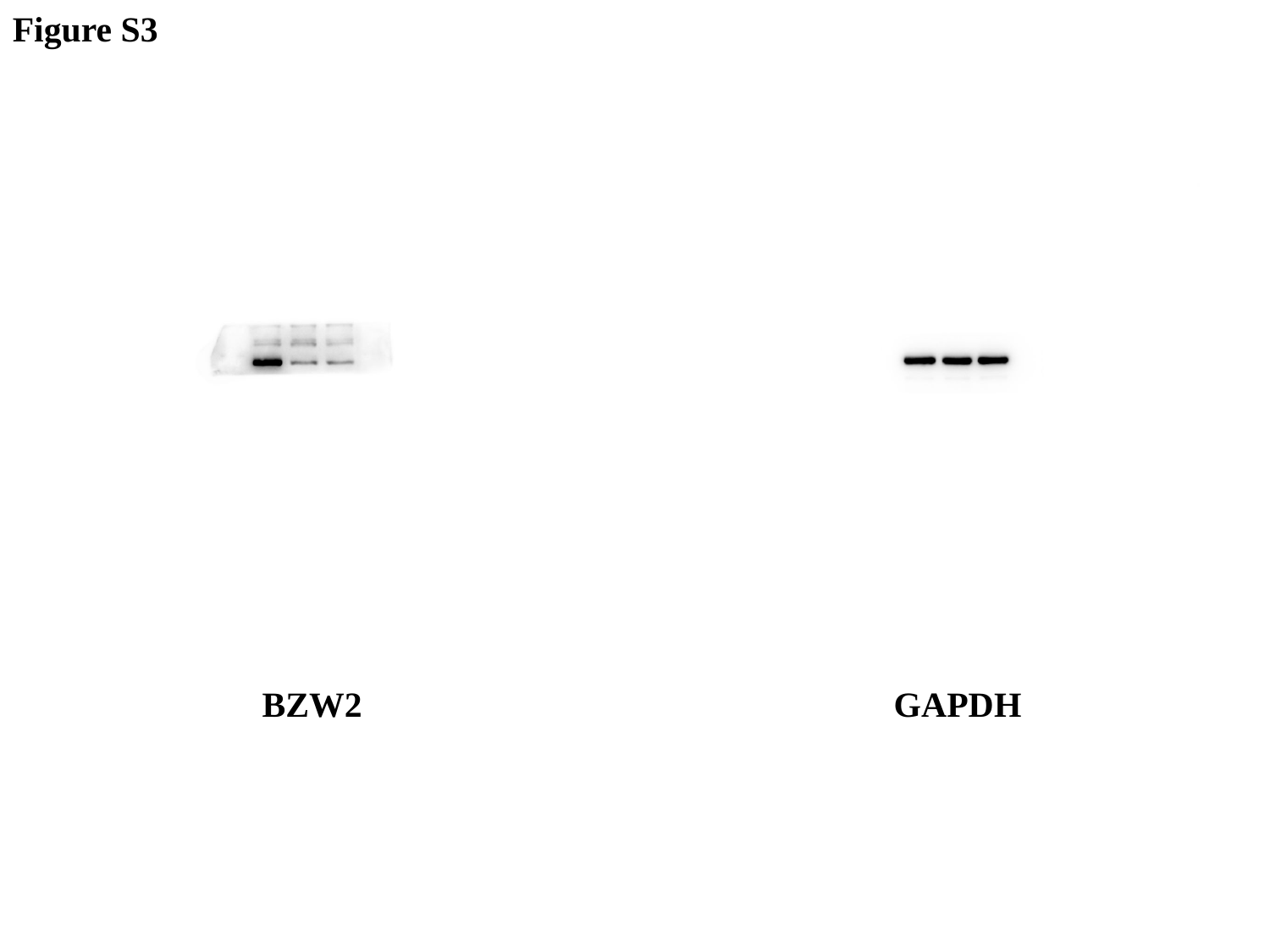

Figure S3
GAPDH
BZW2

## Slide 69
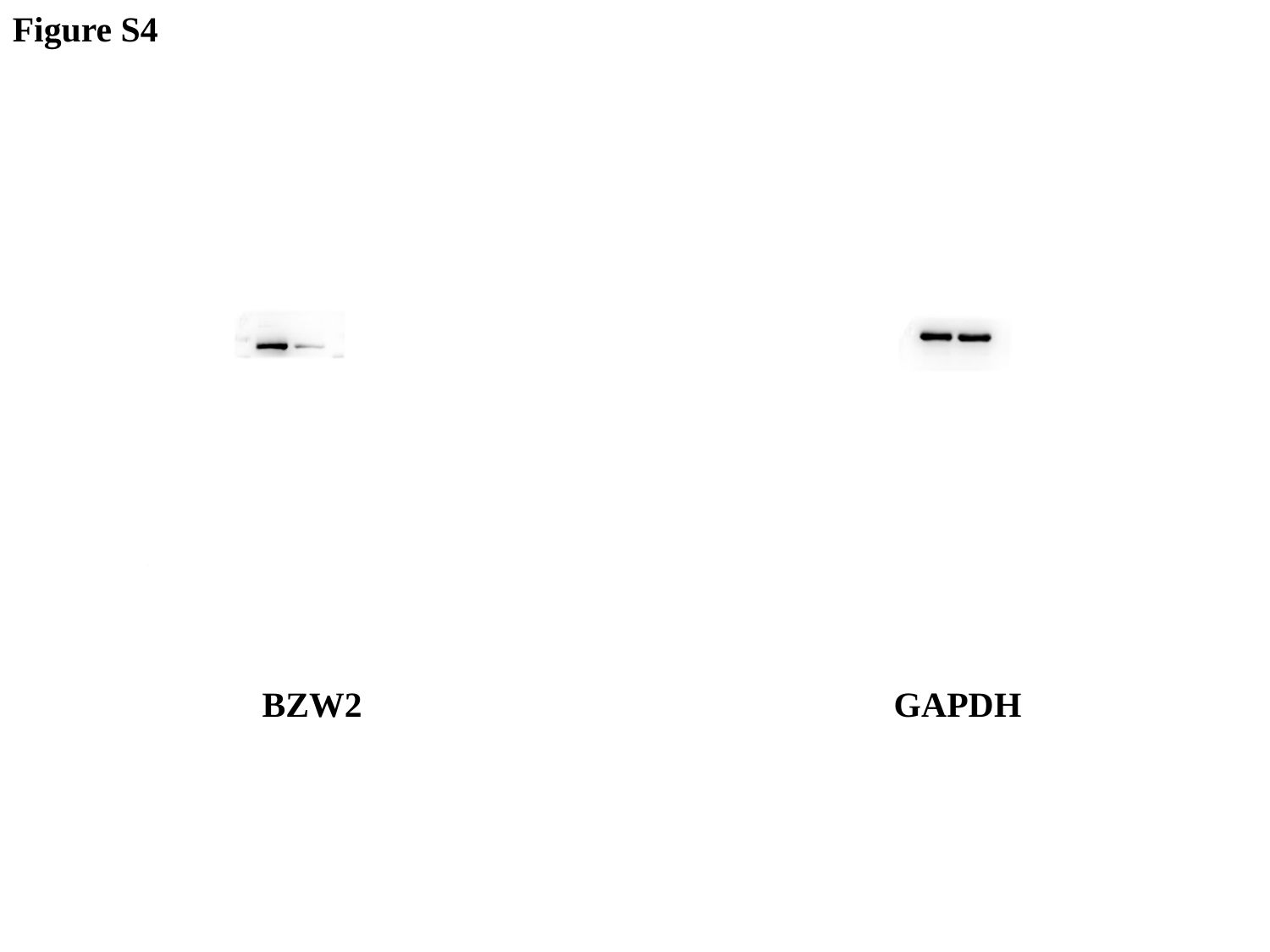

Figure S4
GAPDH
BZW2

## Slide 70
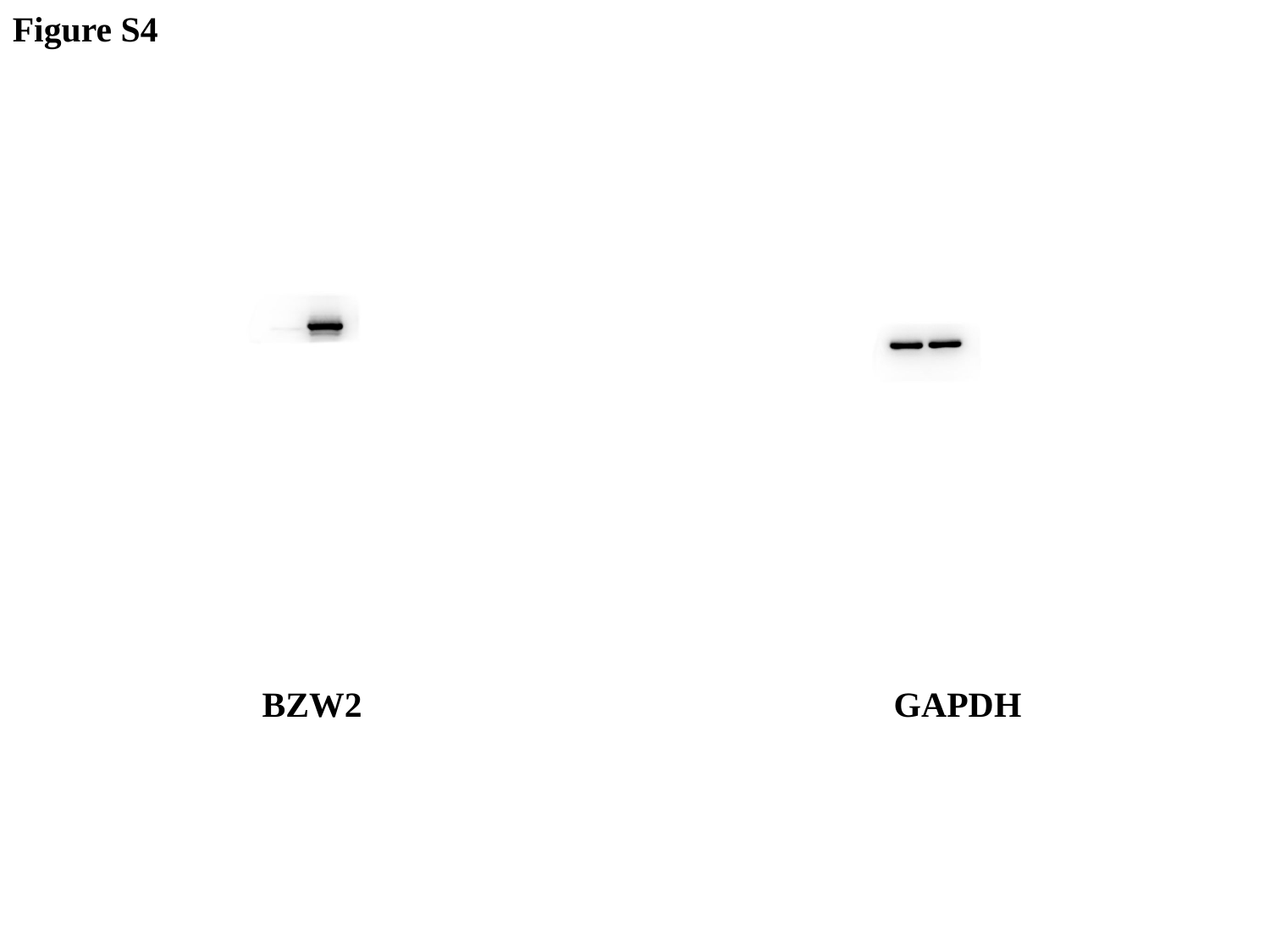

Figure S4
GAPDH
BZW2
